# Modulating Chalcogen Bonding and Halogen Bonding Sigma‐Hole Donor Atom Potency and Selectivity for Halide Anion Recognition

**DOI:** 10.1002/anie.202108591

**Published:** 2021-08-31

**Authors:** Andrew Docker, Charles H. Guthrie, Heike Kuhn, Paul D. Beer

**Affiliations:** ^1^ Department of Chemistry University of Oxford Chemistry Research Laboratory Mansfield Road Oxford OX1 3TA UK

**Keywords:** anion receptors, chalcogen bonding, halogen bonding, Hammett plot analysis, linear free energy relationship

## Abstract

A series of acyclic anion receptors containing chalcogen bond (ChB) and halogen bond (XB) donors integrated into a neutral 3,5‐bis‐triazole pyridine scaffold are described, in which systematic variation of the electronic‐withdrawing nature of the aryl substituents reveal a dramatic modulation in sigma‐hole donor atom potency for anion recognition. Incorporation of strongly electron‐withdrawing perfluorophenyl units appended to the triazole heterocycle telluro‐ or iodo‐ donor atoms, or directly linked to the tellurium donor atom dramatically enhances the anion binding potency of the sigma‐hole receptors, most notably for the ChB and XB receptors displaying over thirty‐fold and eight‐fold increase in chloride anion affinity, respectively, relative to unfluorinated analogues. Linear free energy relationships for a series of ChB based receptors reveal the halide anion recognition behaviour of the tellurium donor is highly sensitive to local electronic environments. This is especially the case for those directly appended to the Te centre (**3⋅ChB**), where a remarkable enhancement of strength of binding and selectivity for the lighter halides is observed as the electron‐withdrawing ability of the Te‐bonded aryl group increases, highlighting the exciting opportunity to fine‐tune anion affinity and selectivity in ChB‐based receptor systems.

## Graphical abstract

Chalcogen (ChB) and halogen bonding (XB) phenyl and aryl functionalised anion receptors exhibit a dramatic modulation in sigma‐hole donor atom potency for anion recognition. Variation of electron‐withdrawing/‐donating properties of triazole appended‐ or directly Te‐bound‐aryl‐substituents facilitated linear free energy relationship (LFER) Hammett plot analysis revealing electronic modulation of halide anion selectivity in sigma‐hole anion receptor design.

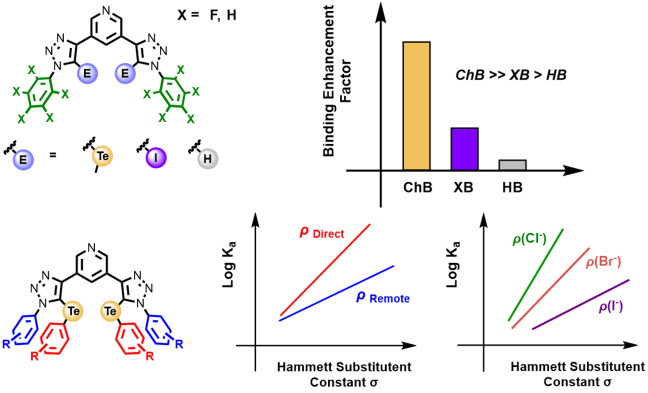

## Introduction

In the context of anion recognition, during the last few decades, anion receptor design has been dominated by the employment of hydrogen bonding (HB).[^1^, ^2^] Indeed, the variety and wealth of HB receptors that utilise a diverse range of HB donor types, have contributed enormously to the current understanding of HB‐anion interactions. In more recent years, sigma‐hole based interactions including halogen bonding (XB) and chalcogen bonding (ChB) are emerging as promising additions to the supramolecular host‐anion guest tool‐box,[^3^, ^4^, ^5^, ^6^, ^7^, ^8^, ^9^, ^10^, ^11^] as well as for self‐assembly and catalysis.[^12^, ^13^, ^14^, ^15^, ^16^, ^17^, ^18^, ^19^, ^20^, ^21^] However, elucidating the influence of key structural and electronic factors for modulating and importantly raising the potency, and molecular engineering selectivity in sigma‐hole based anion recognition remains largely underdeveloped.[^12^, ^20^, ^22^]

**Figure 1:**
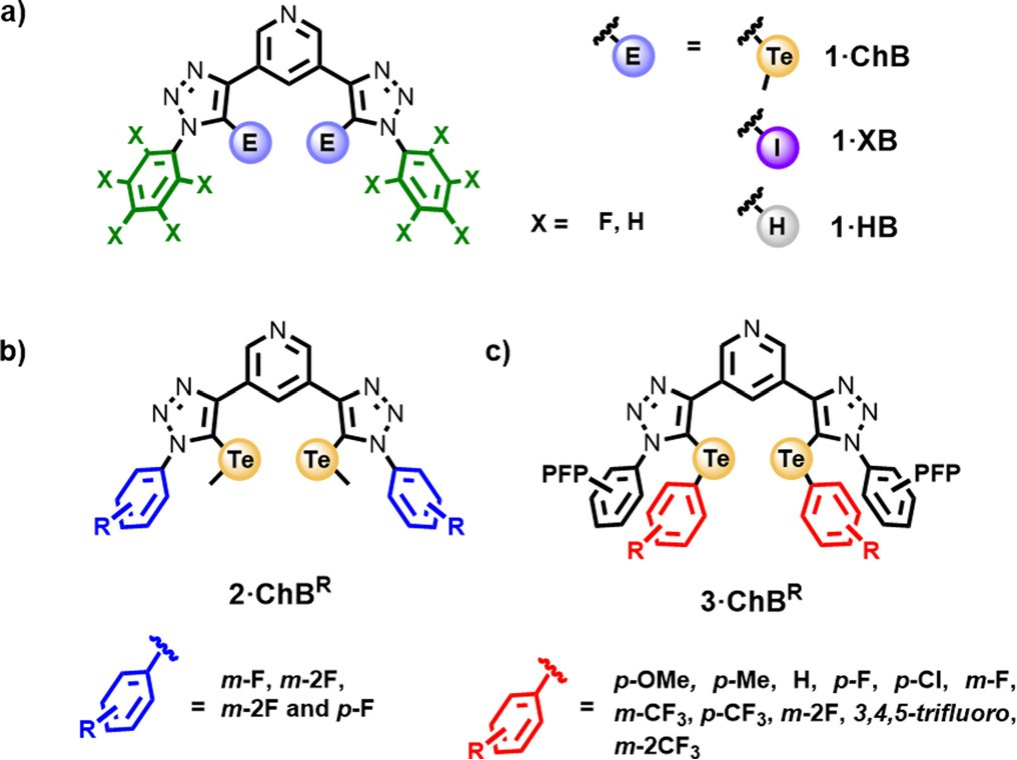
a) Perfluorophenyl and phenyl appended receptor series **1⋅ChB**, **1⋅XB** and **1⋅HB** b) Aryl triazole functionalised of telluromethyl **2⋅ChB** receptor series. c) Te**‐**bound aryl bis**‐**triazole **3⋅ChB** receptor series.

We have recently demonstrated the potency of XB and ChB interactions for ion‐pair recognition is highly sensitive to the local electronic environment of the sigma hole donor atom.[^23^, ^24^] Specifically, co‐bound sodium cation crown ether binding in XB and ChB heteroditopic receptor systems dramatically switches on the recognition of sigma hole donor halide anion interactions. Motivated by these observations, we sought to further investigate the mechanism of sigma hole through‐bond polarisation. To this end we report herein, the synthesis, anion binding studies and linear free energy relationship analysis of a library of structurally homologous ChB, XB and HB acyclic receptor systems. The first generation receptors investigated, **1⋅ChB**, **1⋅XB** and **1⋅HB**, comprise of a 3,5‐bistriazole pyridine motif covalently appended with either phenyl or highly electron deficient perfluorophenyl (PFP) substituents (Figure 1 a). Extensive ^1^H NMR halide anion titration studies reveal the integration of PFP groups dramatically enhance ChB, and to a lesser extent XB, anion binding potency, whereas HB analogues exhibited no measureable effect. Systematically varying the electron‐withdrawing/‐donating characteristics of aryl substituents appended to the triazole heterocycle **2⋅ChB** (Figure 1 b) or directly bonded to the tellurium donor atom **3⋅ChB** (Figure 1 c) facilitated the construction of Hammett plots, establishing linear free energy relationships (LFER), which crucially highlight ChB halide anion affinity and selectivity is highly sensitive to electronic through‐bond modulation of chalcogen bond donor potency.

## Results and Discussion

### Synthesis

**Figure 2:**
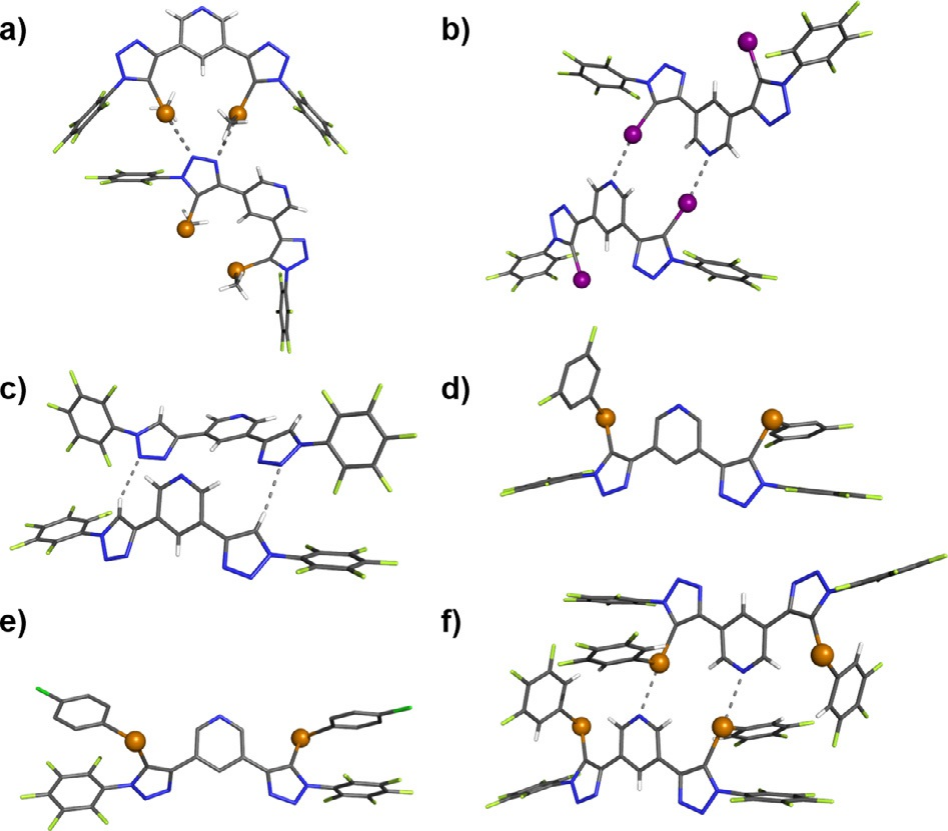
X**‐**ray crystallographic structures of a) **1⋅ChB^PFP^**, b) **1⋅XB^PFP^** and c) **1⋅ChB^PFP^** d) **3⋅ChB^pCl^** e) **3⋅ChB^2F^** f) **3⋅ChB^3F^** showing the formation of ChB/XB/HB interactions in the solid state, represented by dashed lines. Grey=carbon, blue=nitrogen, white=hydrogen, green=fluorine, dark green=chlorine, orange=tellurium, purple=iodine.^[29]^

**Scheme 1:**
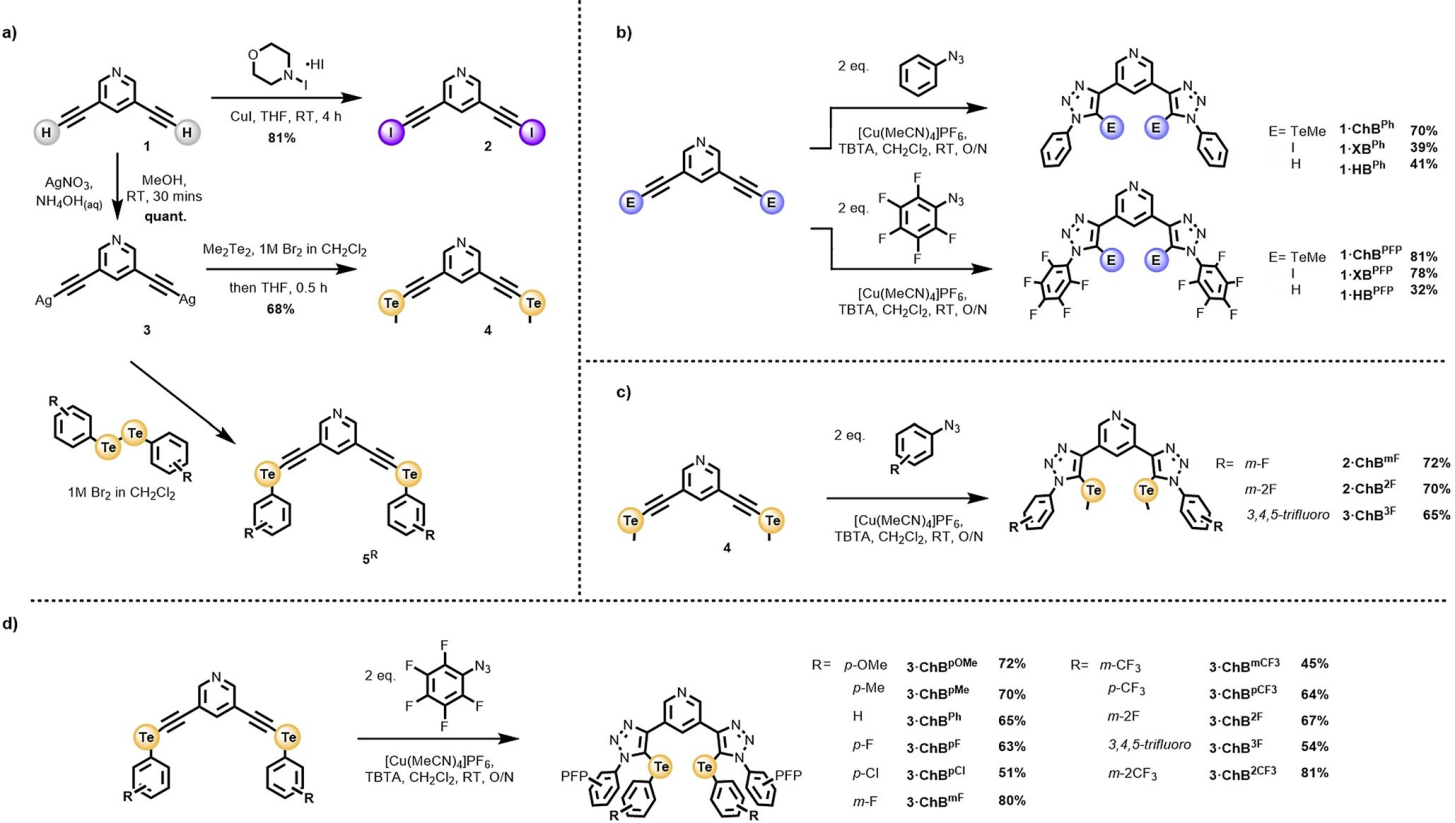
a) Synthesis of the requisite functionalised alkyne derivatives. CuAAC mediated synthesis of the b) **1⋅ChB**, **1⋅XB** and **1⋅HB** series c) **2⋅ChB** series d) **3⋅ChB** series.

The target ChB, XB and HB receptors were prepared via CuAAC methodology (Scheme 1). The requisite iodo‐ and tellurium‐functionalised alkyne precursors, **2** and **4** were synthesized according to Scheme 1, from 3,5‐diethynyl pyridine **1**. Treatment of the proto‐alkyne with *N*‐iodomorpholine hydroiodide in the presence of catalytic copper(I) iodide gave the bis‐iodoalkyne **2** in 81 % yield. Reaction of **1** with freshly pulverised AgNO_3_ in NH_4_OH_(aq)_/MeOH mixtures facilitated precipitation and isolation of bis‐Ag^I^‐acetylide **3**.[^25^, ^26^] Synthesis of the bis‐telluro alkynes was achieved by treatment of the appropriate dialkyl‐ or diaryl‐ ditelluride with a 1 M Br_2_ CH_2_Cl_2_ solution to afford the organo tellurium bromide which was reacted immediately with a THF suspension of **3**, affording the series of bis‐telluro functionalized alkynes **4** and **5^R^** after rapid chromatographic purification. In general the synthesis of all target ChB, XB and HB based receptors was achieved by a CuAAC reaction between the appropriately functionalised alkyne and aryl‐azide, in the presence of catalytic [Cu(MeCN)_4_]PF_6_ and the rate accelerating ligand TBTA in dichloromethane. Subsequent aqueous work‐up procedures of the reaction mixtures and purification of the crude material by column chromatography gave the receptors in yields in the range 41–81 %. The 20 novel anion receptors were characterised by ^1^H NMR, ^13^C NMR, and high resolution ESI‐MS. Crystals of **1⋅XB^PFP^**, **1⋅ChB^PFP^**, **1⋅HB^PFP^**, **3⋅ChB^pCl^**, **3⋅ChB^2F^** and **3⋅ChB^3F^** suitable for X‐ray structural analysis were grown from slow evaporation of chloroform solutions (Figure 2). The structures show the σ‐hole donors participating in intermolecular N⋅⋅⋅I XB or N⋅⋅⋅Te ChB interactions, respectively whilst the **1⋅HB^PFP^** forms linear chains in the solid state directed by triazole C−H⋅⋅⋅N interactions and **1⋅HB^PFP^**.

### Anion Binding Studies of 1⋅ChB, 1⋅XB and 1⋅HB Receptor Series

Preliminary ^1^H NMR spectroscopic titration experiments in [D_6_]acetone were performed to investigate the anion binding properties of the novel **1⋅ChB**, **1⋅XB** and **1⋅HB** receptor series.

**Figure 3:**
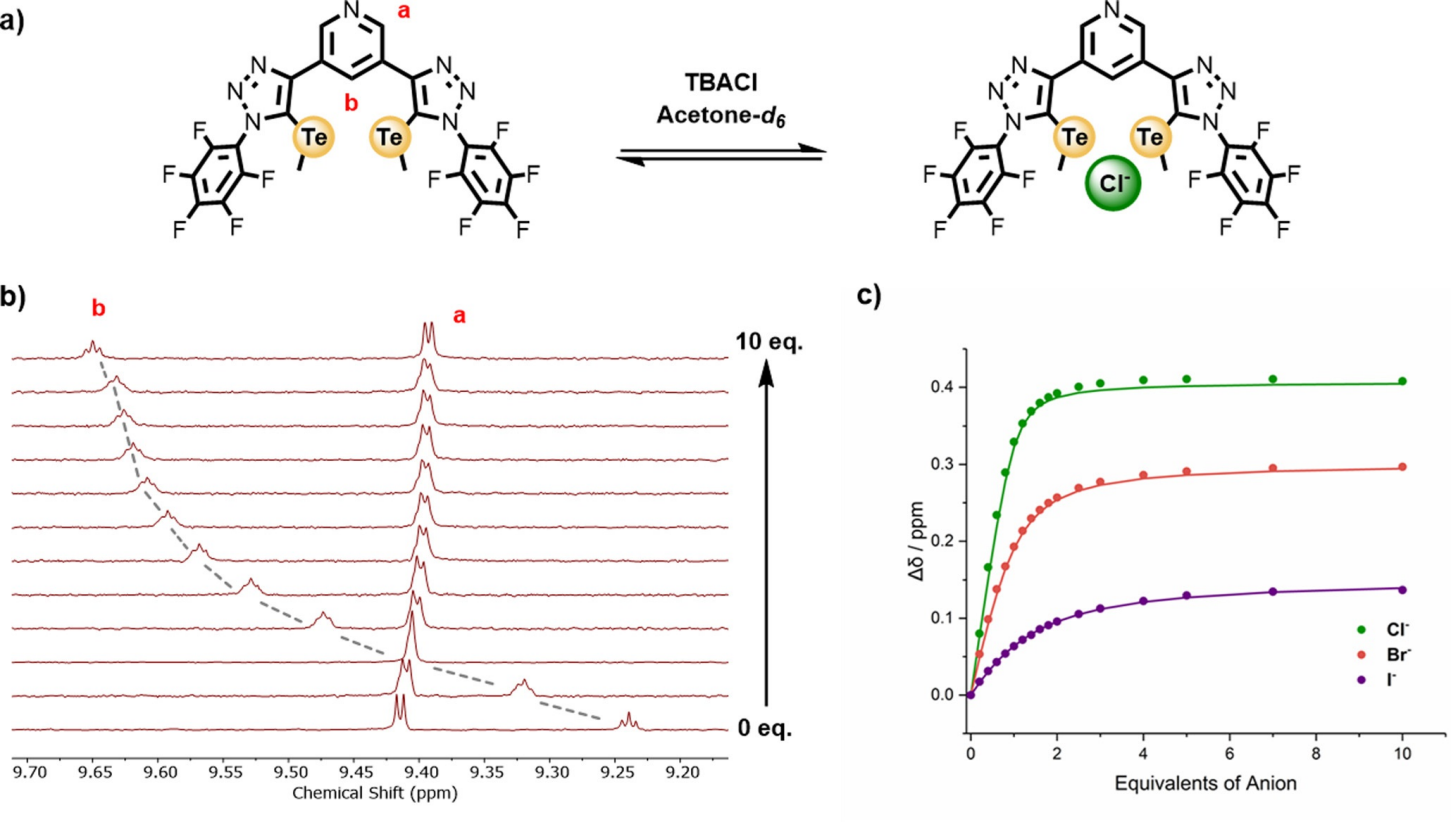
a) Chloride anion binding equilibrium for **1⋅ChB^PFP^**. b) Stacked ^1^H NMR titration spectra for **1⋅ChB^PFP^** with increasing equivalents of TBACl ([D_6_]acetone, 500 MHz, 298 K). c) Anion binding titration isotherm for **1⋅ChB^PFP^** with chloride, bromide and iodide.

Aliquots of halides as their tetrabutylammonium (TBA) salts were titrated against solutions of the six receptors, with significant perturbations in various proton resonances commonly observed. Typically, with the XB and ChB receptors the external pyridyl signal underwent an upfield shift on addition of the TBA halide salts, whereas the internal pyridyl proton universally shifted downfield. Concomitant downfield perturbations in the chemical shift of the triazole C−H and TeMe protons for the HB and ChB receptors, respectively, were also observed upon addition of anion titrant, indicating that the binding event occurs in the bis‐triazole cleft (Figure 3 a and b). Isotherm binding titration data, obtained by monitoring the chemical shift of the respective receptor's internal pyridyl proton b local to the anion binding cavity as a function of anion concentration (Figure 3 c), was analysed using Bindfit^[27]^ to determine 1:1 stoichiometric host:guest halide association constants displayed in Table 1. Halide anion binding affinities correlate with charge density and basicity of the halide ion: Cl^−^ > Br^−^ > I^−^.

**Table 1:** Halide anion association constants and calculated binding enhancement factors for phenyl and pefluorophenyl appended ChB, XB and HB acyclic anion receptor systems.

	Anion association constants *K*_a_ [M^−1^]^[a]^						Binding enhancement factors *α*=(*K*_a_^PFP^/*K*_a_^Ph^)		
Anion^[b]^	**1⋅ChB^PFP^** ^[c]^	**1⋅ChB^Ph^** ^[a]^	**1⋅XB^PFP^** ^[d]^	**1⋅XB^Ph^** ^[d]^	**1⋅HB^PFP^** ^[c]^	**1⋅HB^Ph^** ^[c]^	**ChB**	**XB**	**HB**
Cl^−^	18 500^[e]^	590	2920	416	386	394	32±4	7.0±0.2	0.86±0.06
Br^−^	4620	304	4600	1060	116	191	15±0.5	4.4±0.3	0.76±0.03
I^−^	1230	234	5230	1450	37	49	5.2±0.4	3.6±0.3	0.86±0.05

[a] Association constants determined by Bindfit analysis of internal pyridine proton signal, errors <5 % unless otherwise specified. [b] Anions added as their tetrabutylammonium salts. [c] Conducted in [D_6_]acetone. [d] Conducted in 2.5 % D_2_O‐[D_6_]acetone (v/v). [e] Error <13 %.

**Figure 4:**
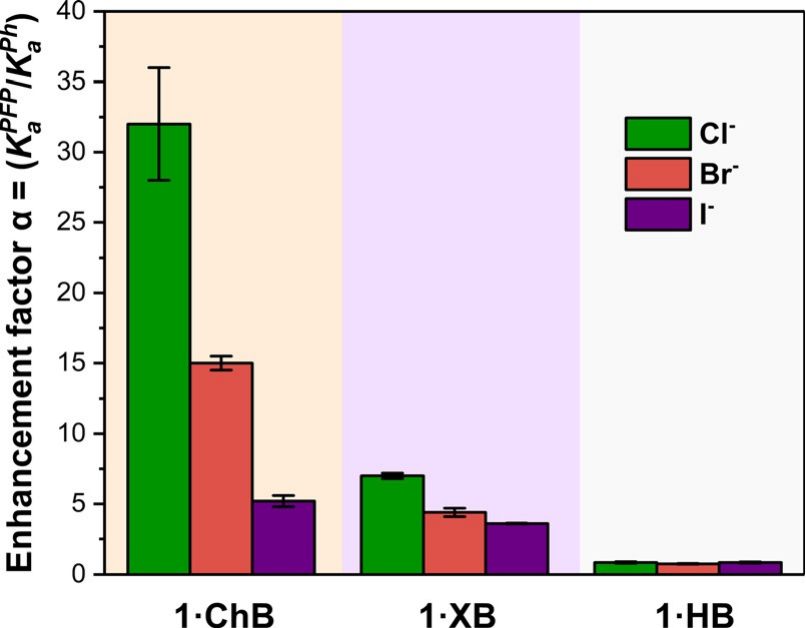
Plot showing the halide anion enhancement factors for the **1⋅ChB**, **1⋅XB** and **1⋅HB** receptor series.

As anticipated, the XB receptors display the greatest halide affinities, followed by the ChB hosts: XB > ChB > HB. Notably, the incorporation of perfluorophenyl substituents in receptors **1⋅XB^PFP^** and **1⋅ChB^PFP^** produces a dramatic enhancement in halide association constant magnitudes relative to their unperfluorinated receptor analogues. Indeed, the remarkable halide anion affinities of perfluorophenyl‐substituted **1⋅XB^PFP^** (>10^5^ M^−1^) precluded accurate quantification of its halide association constants, necessitating the more competitive solvent mixture of 2.5 % D_2_O/[D_6_]acetone (v/v) for their evaluation which resulted in a Hofmeister bias for anion afffinity; I^−^ > Br^−^ > Cl^−^.^[28]^ The incorporation of spacer perfluorophenyl groups to the receptor produces a dramatic enhancement in its halide affinities relative to **1⋅XB^Ph^**.^[27]^ Inspection of Table 1 also reveals a significant enhancement in halide affinity for **1⋅ChB^PFP^** relative to **1⋅ChB^Ph^**, illustrating the influence of the perfluorophenyl electron‐withdrawing groups on the halide affinities of both XB and ChB sigma‐hole type receptors. This is in stark contrast to that observed with the HB receptors, which surprisingly appear unaffected by the incorporation of electron‐withdrawing perfluorophenyl groups in their receptor design. To quantitatively evaluate the effects of perfluorophenyl group incorporation in these receptor systems, halide anion binding enhancement factors (*α*=*K*_a_^PFP^/*K*_a_^Ph^) were calculated, facilitating comparison of the relative augmentation of association constants. Importantly, a survey of the binding enhancement factors (*α*) in Table 1 demonstrates that for all halides the ChB receptor exhibits the largest α value relative to XB and HB analogues, as graphically illustrated in Figure 4.

In particular, α values calculated for chloride reveal over a thirty‐fold enhancement for ChB, whereas XB demonstrates an eight‐fold enhancement. The pronounced increase of anion association constant magnitude by the perfluorophenyl substituent may be rationalised by the increased polarisability of the heavier ChB and XB donor atoms in the C−X bond (X=TeMe, I), relative to the proto‐triazole HB donor. Interestingly, the HB analogue's halide affinities appear surprisingly unperturbed upon incorporation of the highly electron‐withdrawing aryl substituents (*α*≈1), suggesting an apparent insensitivity of the HB donors to electronic substitution. Whilst somewhat surprising and contradictory to the well established doctrine of correlating HB donor acidity with anion binding potency, the observed trend most likely is a consequence of solvation effects and competitive HB formation between the acidic triazole C−H donors and acetone solvent molecules, which is anticipated to be a more pronounced effect in the perfluorinated receptor.

### Anion Binding Studies of 2⋅ChB^R^ Receptor Series

**Figure 5:**
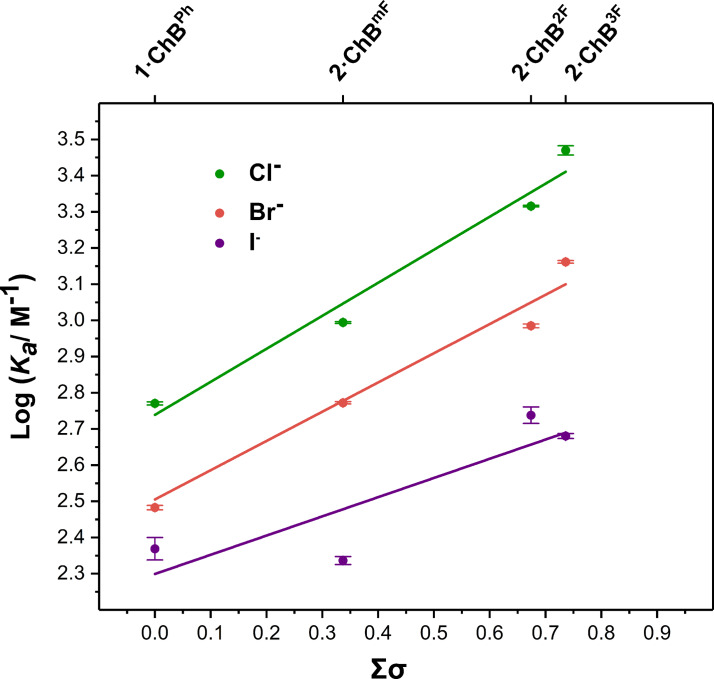
Hammett plot for the **2⋅ChB^R^** receptor series.

The halide anion affinities of the complementary **2⋅ChB** receptor series were determined via ^1^H NMR spectroscopic titration experiments. As expected, increasing the extent of fluorine substitution on the triazole linked aryl substituent results in a concomitant increase in halide association constant (Table 2). Furthermore, the additive nature of Hammett substituent constants (*σ*) facilitated the construction of LFER plots (Figure 5) in which the effect of the increasing electron withdrawing ability of aryl substituents on halide association constant values is quantified, as represented by the *ρ* value, which serves as a measure of the sensitivity of the ChB‐anion interaction to the changing electronic properties of an aryl substituent. Summarised in Table 2, the *ρ*>0 value for all halides indicates that anion binding potency increases as the sum of the electron withdrawing substituent constants (Σ*σ*) increases. The sensitivity of this through‐bond inductive polarisation appears to be most pronounced for the lighter halides, Cl^−^ and Br^−^ with *ρ*=0.91 and 0.81 respectively. Analogous plots constructed with *K*_a_ values for iodide, revealed a much less pronounced correlation with Σ*σ*, possibly due to steric effects implicated in binding the larger halide.

**Table 2:** Halide anion association constants and calculated ρ values from Hammett plot analysis of the **2⋅ChB** receptor series.

	Anion association constants *K*_a_ [M^−1^]^[a]^ for **2⋅ChB^R^** series				
Anion^[b]^	**mF**	**2F**	**3F**	*ρ* ^[c]^	*r* ^[d]^
Cl^−^	987^[e]^	2070	2950^[e]^	0.91±0.11	0.99
Br^−^	592	966	145	0.81±0.11	0.98
I^−^	217	547	497	0.53±0.21	0.87

[a] Association constants determined by Bindfit analysis of internal pyridine proton signal, errors ± ≤7 % conducted in [D_6_]acetone [b] Anions added as their tetrabutylammonium salts. [c] *ρ* refers to the value from the Hammett equation, errors indicated in parenthesis. [d] Pearson's correlation coefficient.

### Anion Binding Studies of 3⋅ChB^R^ Receptor Series

In order to assess the effect of replacing the tellurium methyl group with a more sterically demanding aromatic substituent, preliminary ^1^H NMR spectroscopic titration experiments in [D_6_]acetone were performed on the **3⋅ChB** series, which, in general, revealed significant perturbations observed in the receptors’ aryl and pyridyl proton resonances, with the largest chemical shift perturbations seen with the more electron‐deficient aryl substituent systems, a representative example is shown in Figure S108.

**Figure 6:**
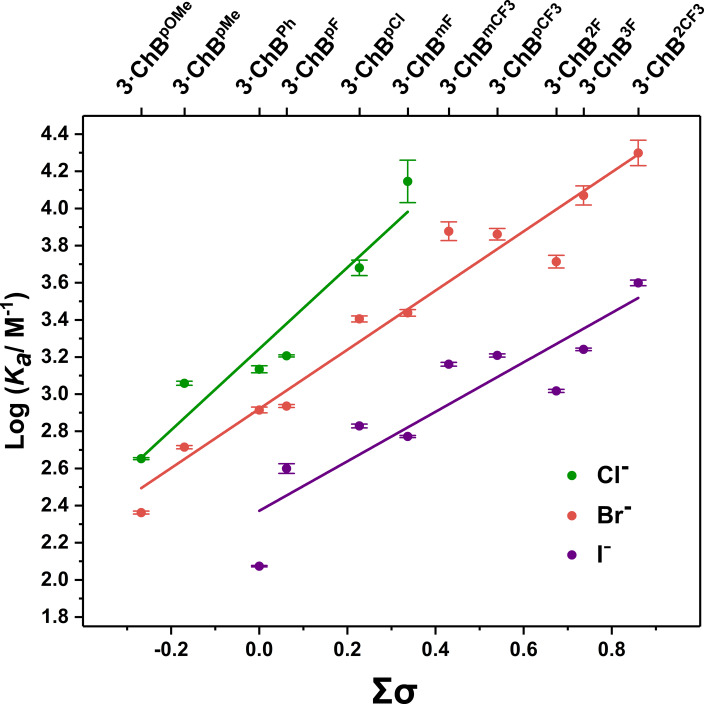
Hammett plot for the **3⋅ChB^R^** receptor series.

Analysis of the anion binding isotherms using the Bindfit programme determined 1:1 stoichiometric association constants summarised in Table 3. Interestingly, comparison of the anion binding properties of **3⋅ChB^Ph^** relative to methyl functionalised analogue, **1⋅ChB^Ph^**, reveal the incorporation of the phenyl group directly linked to the Te donor significantly attenuates the halide affinity, presumably as a consequence of the sterically encumbering aryl group. The anion affinities of the ChB receptors decrease in the series Cl^−^ > Br^−^ > I^−^, which correlates with the decreasing basicity of the halide guests. More importantly, on traversing Table 3 from left to right, a large enhancement in the association constant magnitudes are observed as the electron‐withdrawing ability of the aryl substituents increase. Furthermore, the para‐methyl and para‐methoxy receptors **3⋅ChB^pMe^** and **3⋅ChB^pMeO^** displayed no measurable chemical shift perturbation upon the addition of 10 equivalents of iodide, whereas the remarkable halide affinity (*K*_a_>10^5^ M^−1^) of highly electron‐deficient receptors, such as **3⋅ChB^3F^** and **3⋅ChB^3F^**, precluded accurate quantification of their chloride association constants. LFER plots of halide *K*_a_ values versus Hammett aryl substituent constants (*σ*) (Figure 6) reveal in all cases that receptor‐halide binding affinity correlates well with the aryl substituent *σ*‐parameters.

**Table 3:** Halide anion association constants for the **3⋅ChB^R^** receptor systems.

	Anion association constants *K*_a_ [M^−1^] for **3⋅ChB^R^** series^[a]^												
Anion^[b]^	**pOMe**	**pMe**	**Ph** ^[c]^	**pF**	**pCl**	**mF**	**mCF3**	**pCF3**	**2F** ^[c]^	**3F** ^[c]^	**2CF3**	*ρ* ^[g]^	*r* ^[h]^
Cl^−^	450	1140	1360	1610	4790	14 000^[f]^	>10^5[d]^	>10^5[d]^	>10^5[d]^	>10^5[d]^	>10^5[d]^	2.20±0.29	0.97
Br^−^	230	518	822	863	2540	2740	7540	7270	5180	11 800^[f]^	19 900^[f]^	1.59±0.13	0.97
I^−^	NB^[e]^	NB^[e]^	119	398	674	592	1450	1620	1040	1750	3980	1.28±0.23	0.90

[a] Association constants determined by Bindfit analysis of Te‐aryl chemical shift perturbations unless otherwise specified, errors ≤10 % unless otherwise specified, conducted in [D_6_]acetone. [b] Anions added as their tetrabutylammonium salts. [c] Determined by monitoring of internal pyridine proton signal. [d] Too large to be accurately quantified by^1^H NMR titration. [e] No binding. [f] Error ≤26 %. [g] *ρ* refers to the value from the Hammett equation [h] Pearson's correlation coefficient.

Several trends can be observed from the LFER analysis. As expected from the relative association constant values, the positive gradients indicate that incorporation of more potent electron‐withdrawing aryl substituents in the receptor design universally increases halide anion binding affinities. Importantly, the *ρ* values summarised in Table 3, demonstrate the significant influence of electron‐withdrawing and donating groups on ChB anion binding strength. Moreover, relative to the *ρ* values obtained by variation of the triazole‐appended aryl substituent (*ρ*(Cl^−^)=0.91) in the **2⋅ChB** series, modulating the electronic donating/withdrawing nature of the tellurium directly appended aryl substituent (**3⋅ChB**) has a considerably larger influence on anion binding potency (*ρ*(Cl^−^)=2.20). Intuitively, this is consistent with conventional wisdom regarding the distance dependence of inductive effects. However, it is noteworthy that comparison of the most electron withdrawing and the most electron donating substituents, has the effect of increasing the chloride association constant value by 4 orders of magnitude. Most importantly, the trend *ρ*(Cl^−^) > *ρ*(Br^−^) > *ρ*(I^−^) translates to an enhancement of selectivity for the lighter halides as the electron‐withdrawing ability of the Te‐bonded aryl group increases. This pronounced polarisation‐induced halide anion discrimination presents a unique opportunity for a non‐covalent interaction‐based approach to engineering selectivity in ChB anion receptors.

## Conclusion

In summary, a library of acyclic anion receptors containing chalcogen bond (ChB) and halogen bond (XB) donors integrated into a neutral 3,5‐bis‐triazole pyridine scaffold appended with various phenyl and electron‐withdrawing fluoroaryl substitutents have been prepared for halide anion recognition investigation. In the first receptor series **1⋅ChB**, **1⋅XB** and **1⋅HB**, the replacement of the triazole‐phenyl substituents with perfluorinated analogues elicits dramatic halide anion affinity enhancements for the sigma‐hole based, ChB and XB receptors, demonstrating an impressive 32‐fold and 8‐fold increase in chloride anion affinity, respectively. Importantly, systematic variation of the electron‐withdrawing potency of aryl‐triazole and Te‐bound‐aryl groups facilitated the construction of Hammett plots, establishing linear free energy relationships between Σ*σ* and determined anion *K*_a_ values. Determined *ρ* values of the **2⋅ChB** and **3⋅ChB** receptor series (*ρ*(Cl^−^)=0.91 and 2.20 respectively), demonstrate that the tellurium ChB donor is highly sensitive to modulation of local electronic environments, which is most pronounced for those directly appended to the Te centre. In the case of **3⋅ChB** the observed trend *ρ*(Cl^−^) > *ρ*(Br^−^) > *ρ*(I^−^) translates to a remarkable enhancement of selectivity for the lighter halides as the electron‐withdrawing ability of the Te‐bonded aryl group increases. Most importantly, these results serve to highlight that through considered selection of electron‐withdrawing aryl substituents covalently linked to the ChB donor atom, exquisite control over both anion binding potency and selectivity is achievable, providing an exciting opportunity for tuning non‐covalent interactions in anion receptor design.

## Conflict of interest

The authors declare no conflict of interest.

## Supporting information

As a service to our authors and readers, this journal provides supporting information supplied by the authors. Such materials are peer reviewed and may be re‐organized for online delivery, but are not copy‐edited or typeset. Technical support issues arising from supporting information (other than missing files) should be addressed to the authors.

Supporting Information
