# Supplementary material for: Modulating Chalcogen Bonding and Halogen Bonding Sigma‐Hole Donor Atom Potency and Selectivity for Halide Anion Recognition
Source: Angew Chem Int Ed Engl. 2021 Aug 31;60(40):21973–8. doi: 10.1002/anie.202108591 (PMC8518858; doi:10.1002/anie.202108591)
Supplement: Supplementary file 1 — Supporting Information [file ANIE-60-21973-s001.pdf]

Angewandte Chemie
Eine Zeitschrift der Gesellschaft Deutscher Chemiker

Supporting Information

Modulating Chalcogen Bonding and Halogen Bonding Sigma-Hole Donor Atom Potency and Selectivity for Halide Anion Recognition

*Andrew Docker, Charles H. Guthrie, Heike Kuhn, and Paul D. Beer**

anie_202108591_sm_miscellaneous_information.pdf

Table of Contents

Materials and methods 2
Synthetic Procedures and Characterisation 3
^{1}H NMR Titration Experiments 83
Crystal Structure Determination 109
Representative examples and summarised data 118
References 124

Materials and methods

All solvents and reagents were purchased from commercial suppliers and used as received unless otherwise stated. Dry solvents were obtained by purging with nitrogen and then passing through an MBraun MPSP-800 column. H_2O was de-ionized and micro filtered using a Milli-Q ® Millipore machine. Column chromatography was carried out on Merck® silica gel 60 under a positive pressure of nitrogen. Routine NMR spectra were recorded on either a Varian Mercury 300, a Bruker AVIII 400 or a Bruker AVIII 500 spectrometer with ^{1}H NMR titrations recorded on a Bruker AVIII 500 spectrometer. TBA salts were stored in a vacuum desiccator containing phosphorus pentoxide prior to use. Where mixtures of solvents were used, ratios are reported by volume. Chemical shifts are quoted in parts per million relative to the residual solvent peak. Mass spectra were recorded on a Bruker µTOF spectrometer. Triethylamine was distilled from and stored over potassium hydroxide. Tris[(1-benzyl-1H-1,2,3-triazol-4-yl)methyl]amine (TBTA). Phenyl azide, pentafluorophenyl azide 1-azido-3-fluorobenzene, 1-azido-3,5-difluorobenzene, 5-azido-1,2,3-trifluorobenzene were prepared according to literature procedures.[1] 3,5-diethynyl pyridine **1** and 3,5-diiodoethynyl pyridine **2**, were prepared according to previous literature reports.[2]

Synthetic Procedures and Characterisation

General Procedure 1

Freshly cleaned Mg turnings (10 mmol) were added a to dry round bottom flask to which was added a few iodine crystals under an atmosphere of nitrogen. The flask was heated until to the point at which purple iodine vapour appeared at which point heating was stopped. To this mixture was added anhydrous THF (20 ml) and left to stir for 10 minutes. After which time a few drops of the appropriate aryl bromide was added and the mixture sonicated and stirred until the colour of the I_2 was no longer visible. After which the remaining aryl bromide was added dropwise (total 10 mmol) to the reaction mixture and upon completion left to stir for 1 hour. The reaction flask was subsequently opened and Te powder (10 mmol) was added in one portion. The reaction mixture was sonicated and stirred until a green colour appeared after which time the mixture was left to stir for a further hour. The mixture was diluted with sat. $NH_4Cl_{(aq)}$ (ca. 10 ml) and subsequently diluted with CH_2Cl_2 (150 ml), the organic phase was collected dried over $MgSO_4$ and the solvent removed in vacuo. The crude material was purified by column chromatography eluting with CH_2Cl_2/hexane mixtures. The target ditelluride, was isolated as a red/brown solid.

General Procedure 2

5 mmol of bromobenzene is added to a stirring solution of NaHTe (prepared from 10 mmol of tellurium powder and 0.45g of sodium borohydride in 20 ml of DMF heating to 90 °C for 0.5 hr). The mixture is stirred for 7 hr at 90°C, after which time 20 ml of water is added. The mixture is stirred for 30 minutes in air. The mixture was then diluted with Et_2O (300 ml) and water (300 ml), the organic phase collected and the aqueous washed with Et_2O (300 ml), the collected organic phases are dried over $MgSO_4$ and concentrated in vacuo and was purified by column chromatography eluting with CH_2Cl_2/hexane mixtures. The target ditelluride, was isolated as a red/brown solid.

General Procedure 3

$[Cu(MeCN)_4]PF_6$ (0.2 equivalents) and TBTA (0.2 equivalents) were dissolved in the minimum amount anhydrous degassed CH_2Cl_2 (ca. 5 ml) and left to stir for 15 minutes. After which time the appropriate alkyne was added to the solution as a solid, followed by the appropriate aryl azide (2.2 equivalents). Once complete, as determined by TLC analysis, the reaction mixture was diluted with CH_2Cl_2 (ca. 150 ml) and washed with EDTA/$NH_4OH_{(aq)}$ solution (20 ml) and water (50 ml), the collected organic phase was dried over $MgSO_4$ and concentrated to dryness and purified by 'flash' silica gel column chromatography to afford the target products as solids.

General Procedure 4

3,5-diethynyl pyridine (0.30 mmol) was dissolved in the minimum amount of MeOH (ca. 10mL) to which was added freshly ground $AgNO_3$ (1.05 equivalents per alkyne) followed by one drop of conc. $NH_4OH_{(aq)}$ (ca. 0.1 mL) immediately inducing precipitation of the desired silver acetylide. The reaction mixture was left to stir for 30 minutes at room temperature, after which time the solvent was removed in vacuo. The silver acetylide was subsequently suspended in anhydrous THF (20 mL) under an atmosphere of nitrogen. In a separate flask the appropriate diorgano ditelluride R_2Te_2 (1 eq.) was dissolved in THF (5 ml) and cooled to 0°C, to the solution of the ditelluride 1 M of Br_2 in CH_2Cl_2 (1 eq.) was added dropwise, after which time the ditelluride solution darkened indicating the formation of RTeBr, which was added immediately to the suspension of the silver acetylide dropwise. The reaction mixture was left to stir for 30 minutes at room temperature in the absence of light, after which time it was filtered through celite and the reaction mixture was concentrated to dryness in vacuo. The crude telluromethyl/aryl alkyne was subjected to rapid column chromatography and used immediately for the following CuAAC reaction.

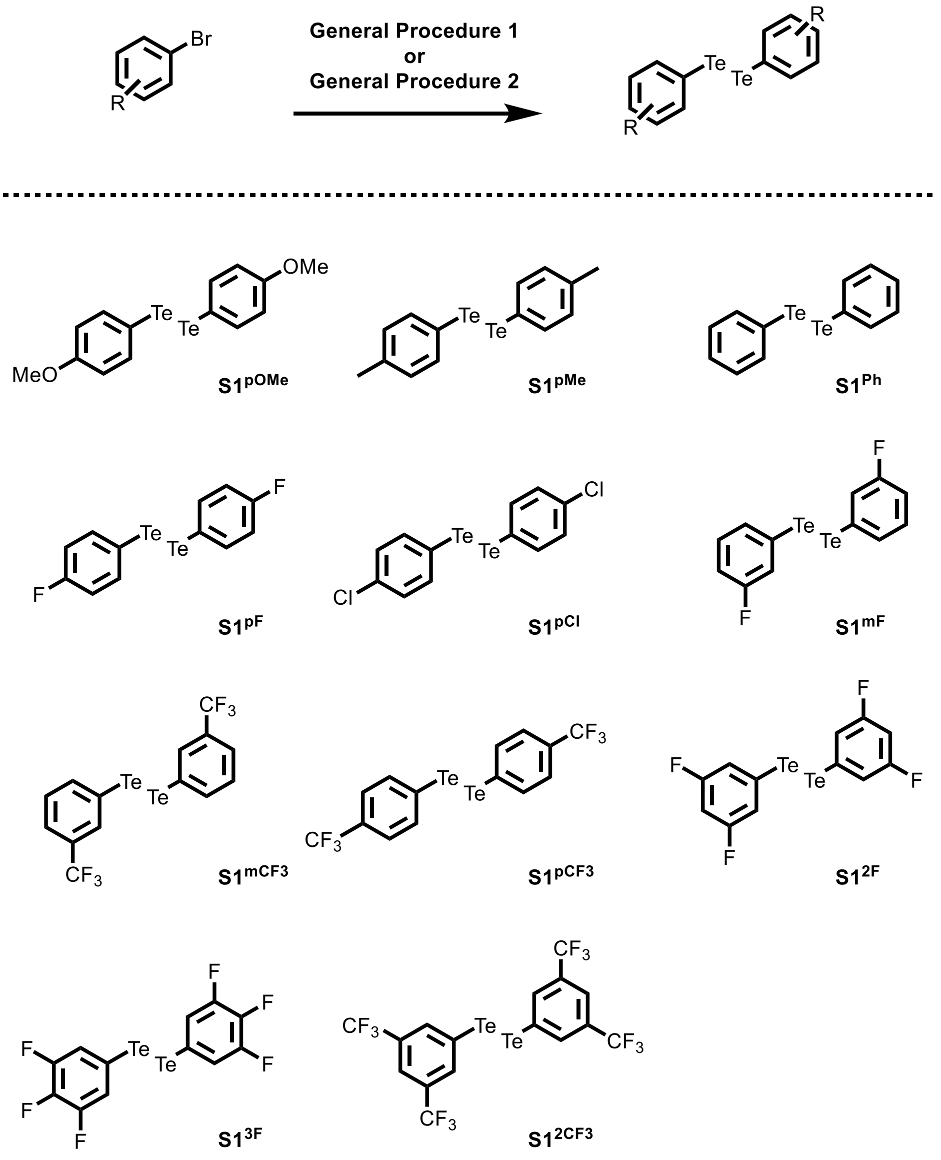

Scheme S1. Synthesis of $\mathbf{S1^{R}}$ ditelluride precursors.

$\mathbf{S1^{pOMe}}$, $\mathbf{S1^{pMe}}$, $\mathbf{S1^{Ph}}$, $\mathbf{S1^{pF}}$, $\mathbf{S1^{mF}}$, $\mathbf{S1^{mCF3}}$, $\mathbf{S1^{2F}}$, $\mathbf{S1^{3F}}$ and $\mathbf{S1^{2CF3}}$ were synthesised according to general procedure 1.[3]

$\mathbf{S1^{Cl}}$, and $\mathbf{S1^{pCF3}}$ were synthesised according to general procedure 2.[4]

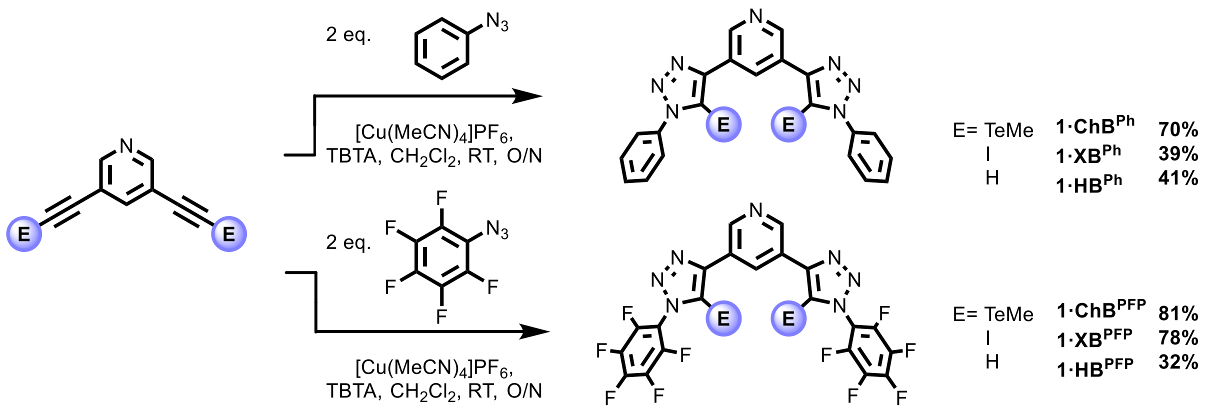

Scheme S2. Synthesis of the **1·ChB**, **1·XB** and **1·HB** receptor series.

1·XB^{Ph}, **1·HB^{Ph}**, **1·XB^{PFP}** and **1·HB^{PFP}** were synthesised according to general procedure 3.

1·ChB^{Ph} and **1·ChB^{PFP}** were synthesised according to general procedure 4.

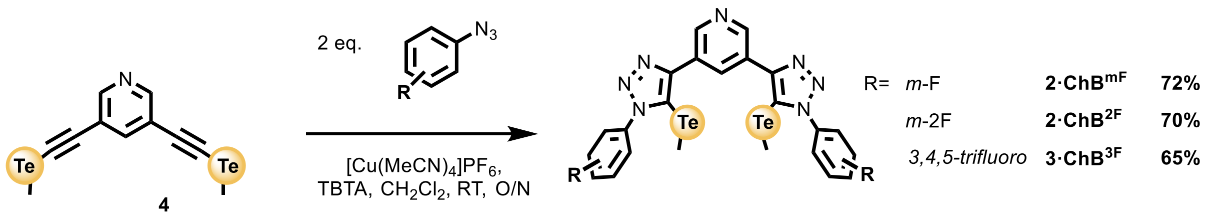

Scheme S3. Synthesis of the **2·ChB**$^{\mathbf{R}}$ receptor series.

2·ChB$^{\mathbf{mF}}$, **2·ChB**$^{\mathbf{2F}}$ and **2·ChB**$^{\mathbf{3F}}$ were synthesised according to general procedure 4.

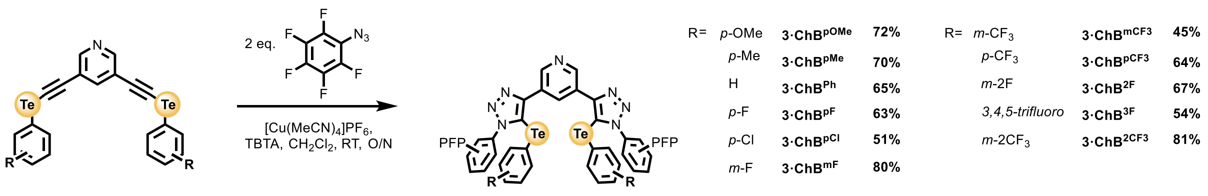

Scheme S4. Synthesis of the **3·ChB^{R}** receptor series.

3·ChB^{pOMe}, **3·ChB^{pMe}**, **3·ChB^{Ph}**, **3·ChB^{pF}**, **3·ChB^{pCl}**, **3·ChB^{mF}**, **3·ChB^{mCF3}**, **3·ChB^{pCF3}**, **3·ChB^{2F}**, **3·ChB^{3F}** and **3·ChB^{2CF3}** were synthesised according to general procedure 4.

1·XB^{Ph}

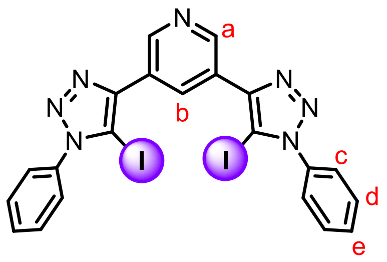

^{1}H NMR (400 MHz, $CDCl_3$) δ = 9.38 (s, 2H_a), 9.09 (s, 1H_b), 7.63 – 7.58 (m,10$H_{c,d,e}$).

^{13}C NMR (126 MHz, $CDCl_3$) δ = 148.55, 147.69, 136.92, 133.59, 130.63, 129.64, 126.64, 126.39, 78.78.

HRMS (ESI +ve) m/z: 617.9390, ($[M+H]^+$, $C_{21}H_{14}N_7I_2$ requires 617.9395).

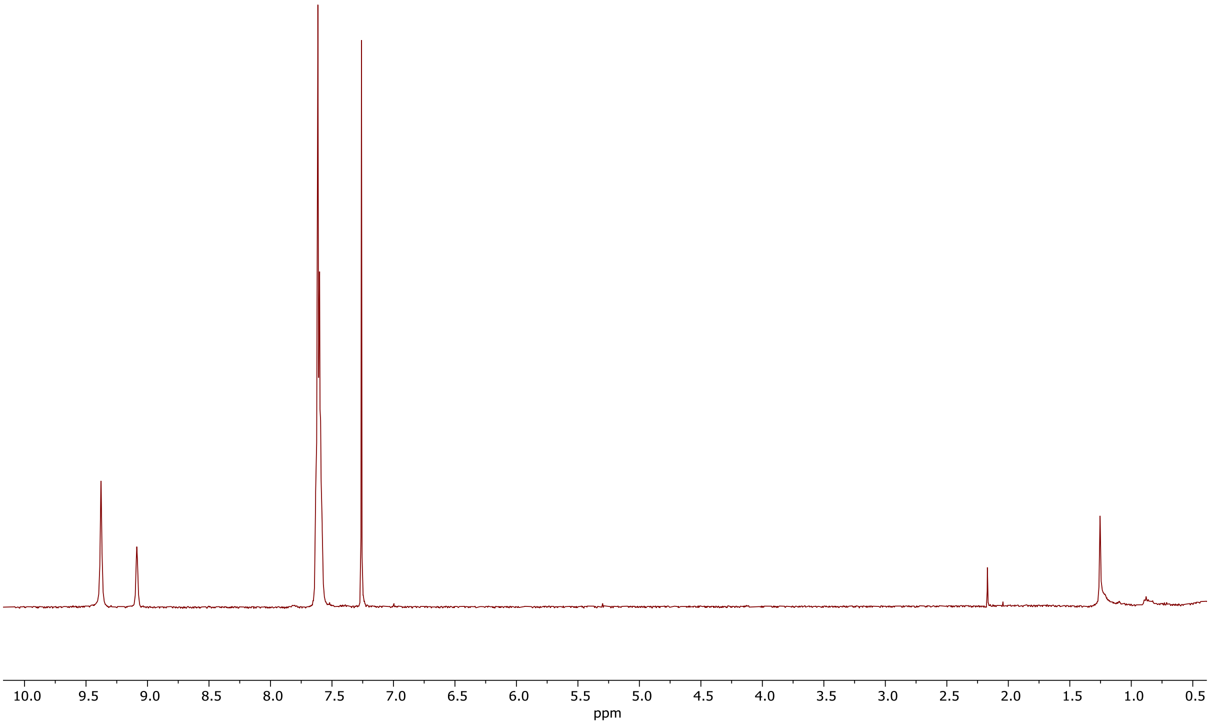

Figure S1. ^{1}H NMR spectrum of **1·XB^Ph** ($CDCl_3$, 400 MHz, 298 K).

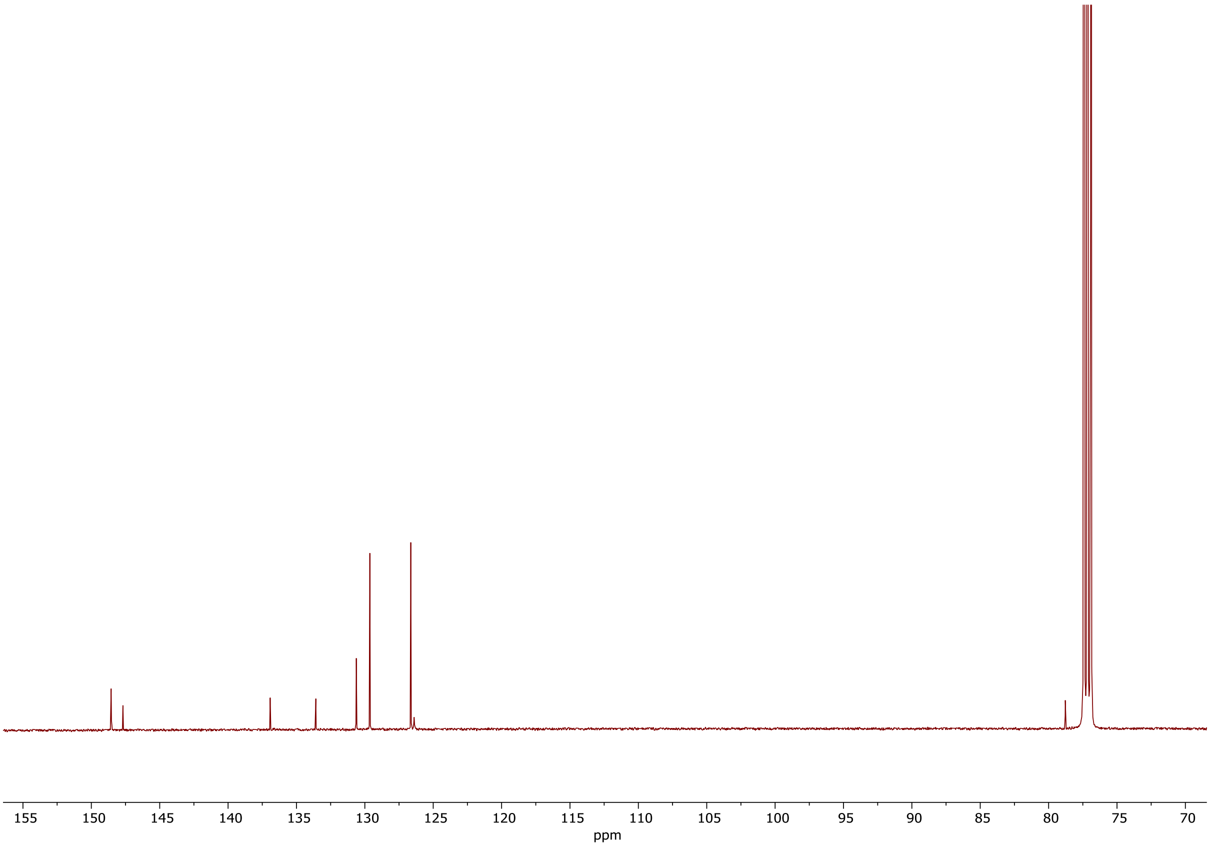

Figure S2. ^{13}C NMR spectrum of **1·XB^Ph** ($CDCl_3$, 126 MHz, 298 K).

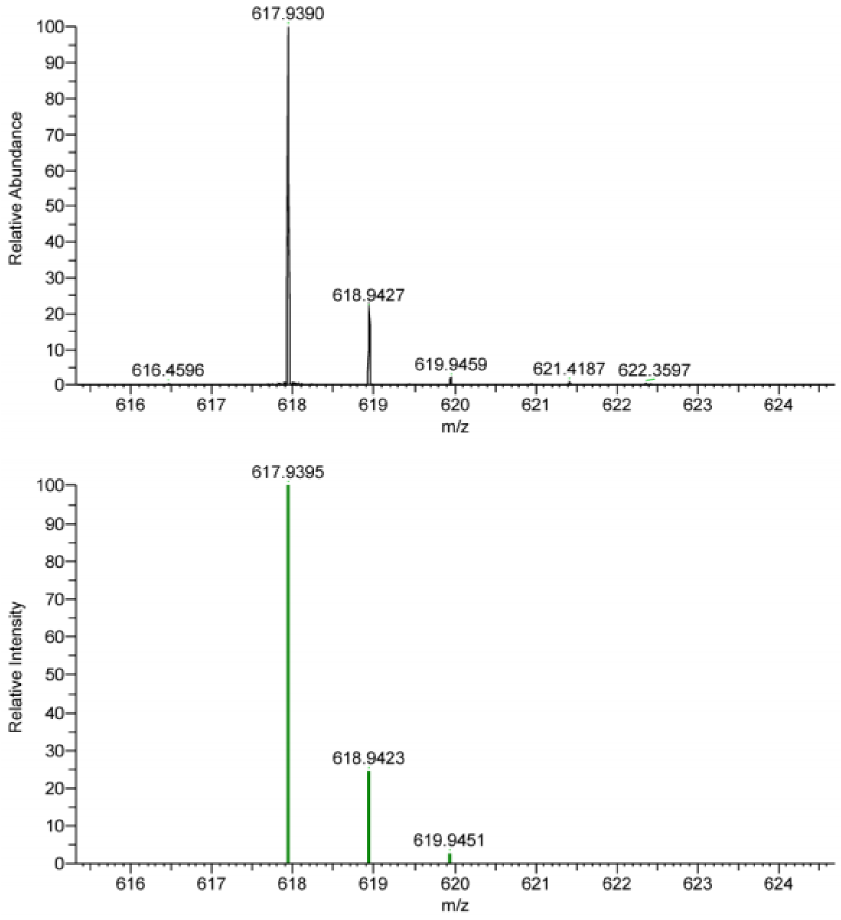

Figure S3. HRESI-MS of **1·XB^{Ph}**.

1·ChB^{Ph}

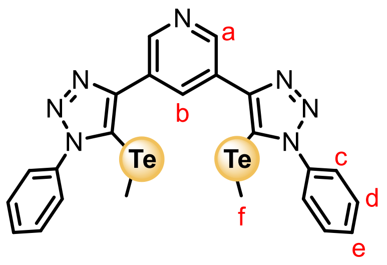

^{1}H NMR (400 MHz, $CDCl_3$) δ = 9.31 (d, *J* = 2.1 Hz, $2H_a$), 8.99 (t, *J* = 2.1 Hz, $1H_b$), 7.60 (s, $10H_{c,d,e}$), 1.85 (s, $6H_f$).

^{13}C NMR (126 MHz, $CDCl_3$) δ = 150.91, 148.79, 137.91, 134.86, 130.17, 129.48, 127.43, 126.40, 100.77, -12.80.

HRMS (ESI +ve) m/z: 649.9868, ($[M+H]^+$, $C_{23}H_{20}N_7Te_2$ requires 649.9870).

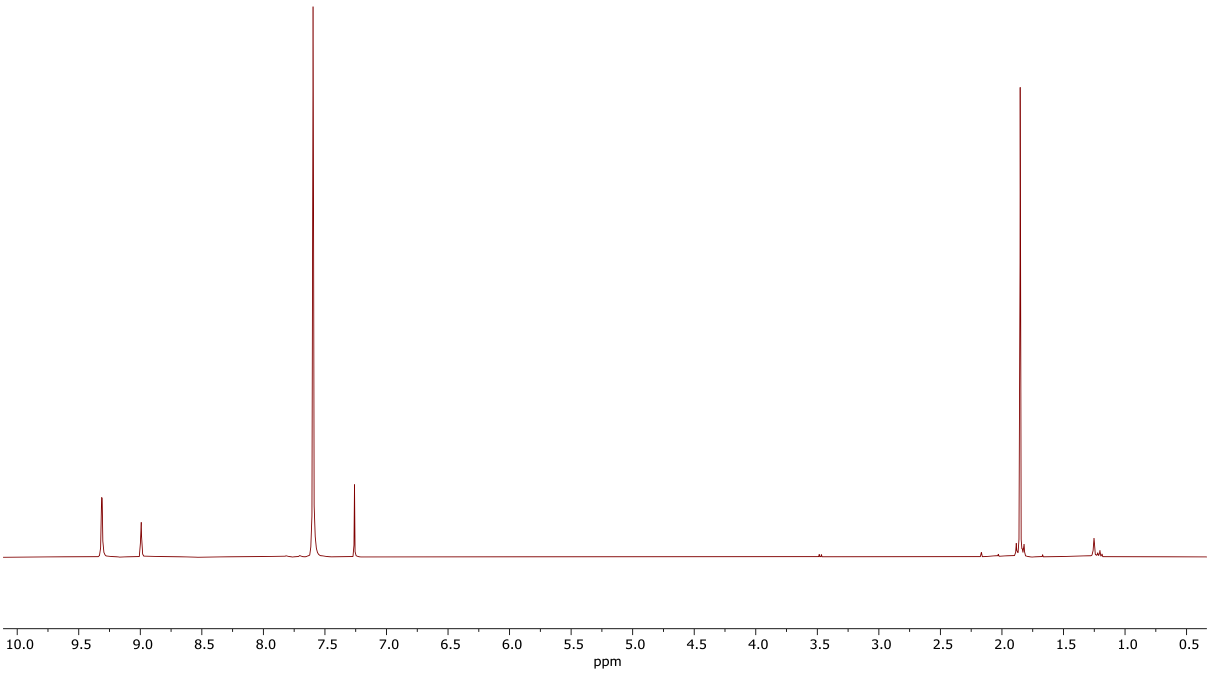

Figure S4. ^{1}H NMR spectrum of **1·ChB^{Ph}** ($CDCl_3$, 400 MHz, 298 K).

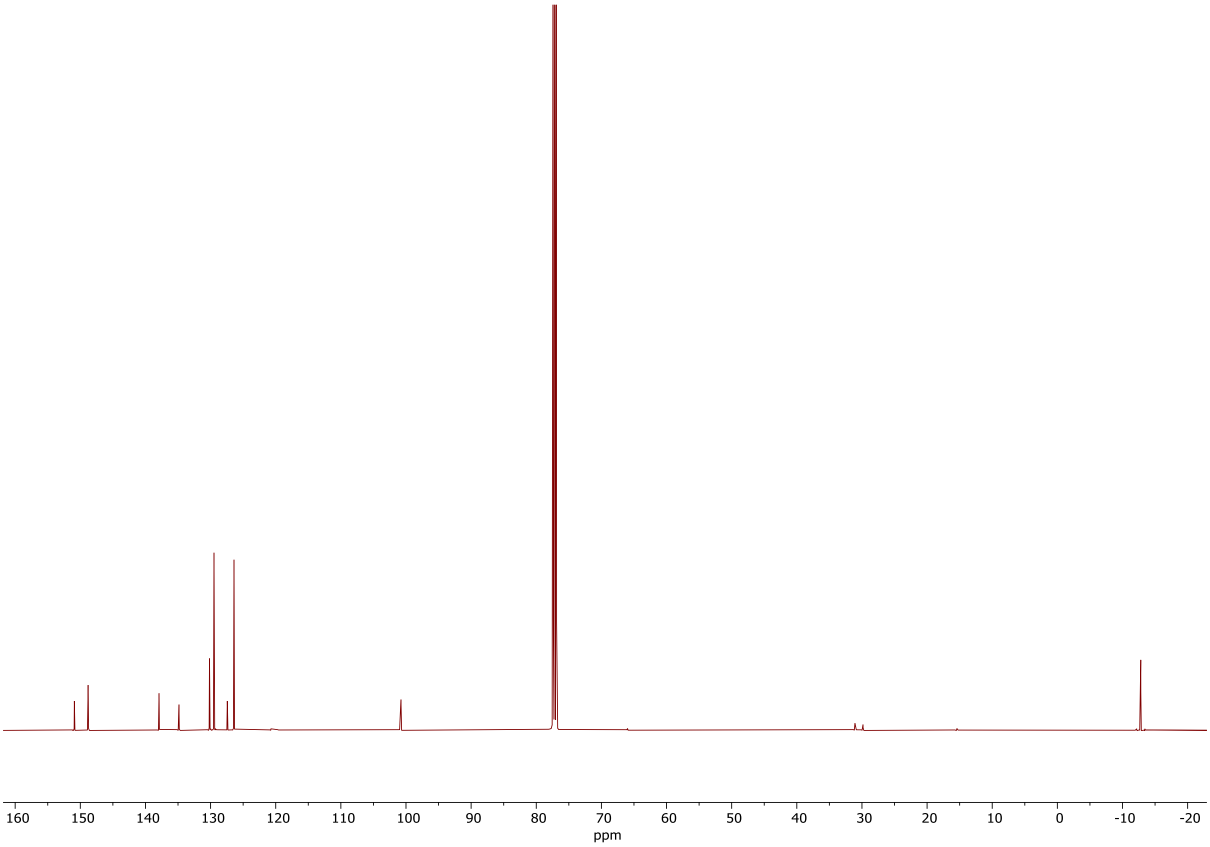

Figure S5. ^{13}C NMR spectrum of **1·ChB^{Ph}** ($CDCl_3$, 126 MHz, 298 K).

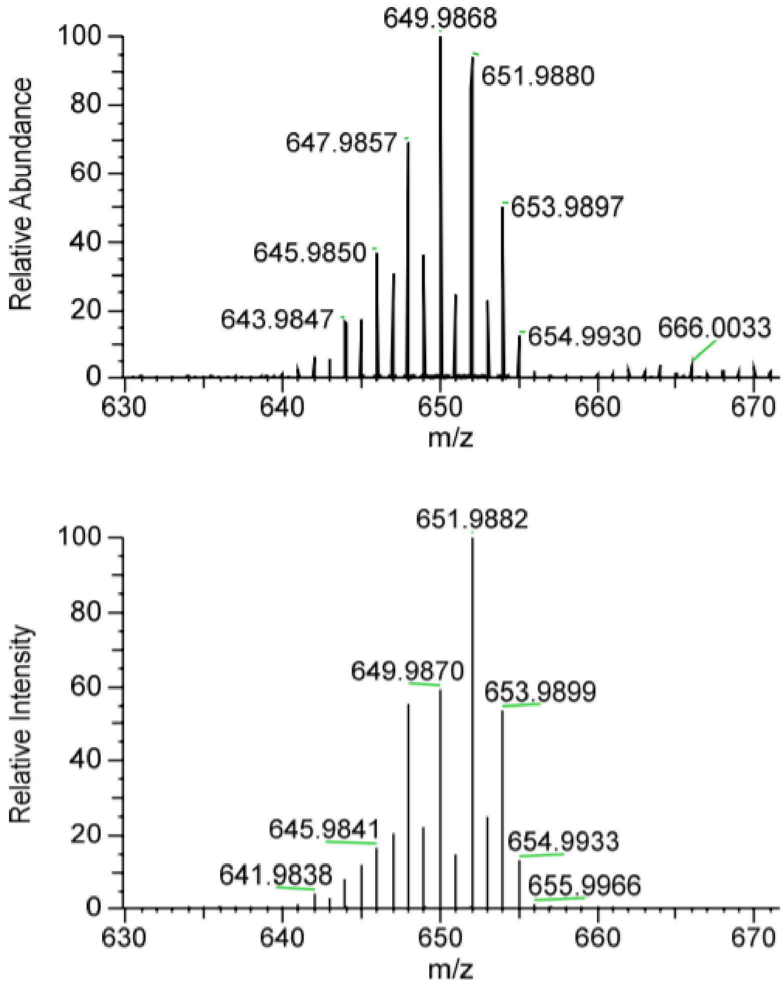

Figure S6. HRESI-MS of **1·ChB^{Ph}**.

1·HB^{Ph}

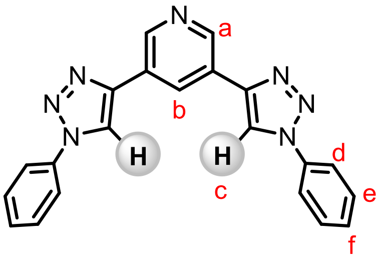

^{1}H NMR (400 MHz, $CDCl_3$) δ = 9.16 (d, *J* = 2.1 Hz, $2H_a$), 8.80 (t, *J* = 2.1 Hz, $1H_b$), 8.40 (s, $2H_c$), 7.84 (d, *J* = 7.5 Hz, $4H_d$), 7.60 (t, *J* = 7.6 Hz, $4H_e$), 7.51 (t, *J* = 7.3 Hz, $2H_f$).

^{13}C NMR (126 MHz, DMSO) δ = 146.07, 144.21, 136.53, 130.05, 129.01, 128.63, 126.58, 120.82, 120.15.

HRMS (ESI +ve) m/z: 366.1464, ($[M+H]^+$, $C_{21}H_{16}N_7$ requires 366.1462).

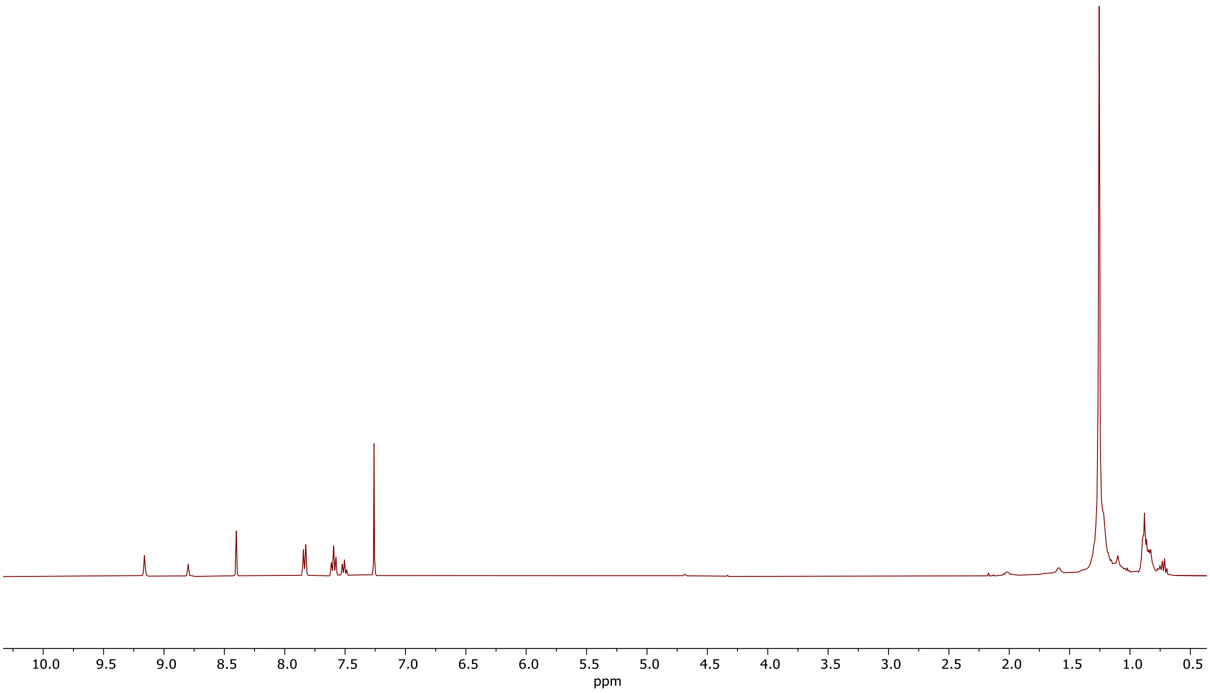

Figure S7. ^{1}H NMR spectrum of **1·HB^{Ph}** ($CDCl_3$, 400 MHz, 298 K).

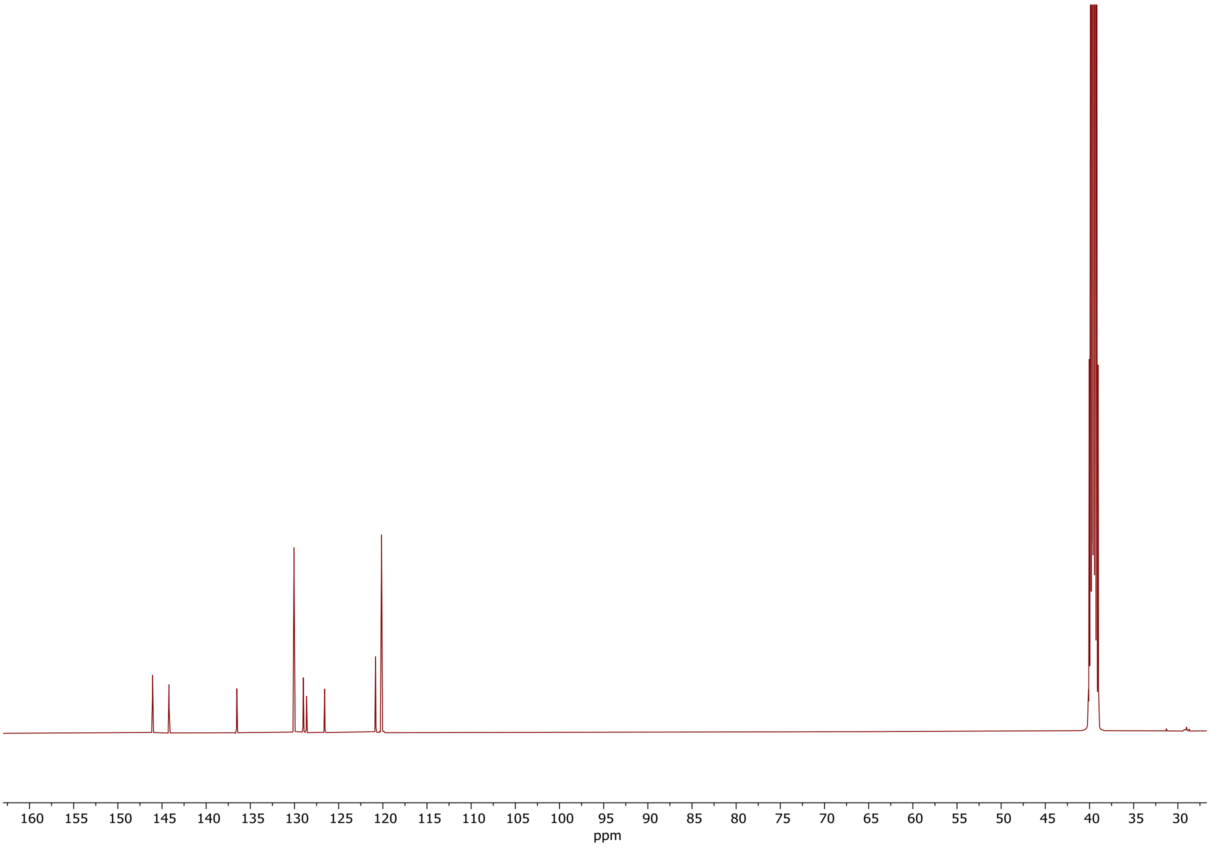

Figure S8. ^{13}C NMR spectrum of **1·HB^{Ph}** (DMSO, 126 MHz, 298 K).

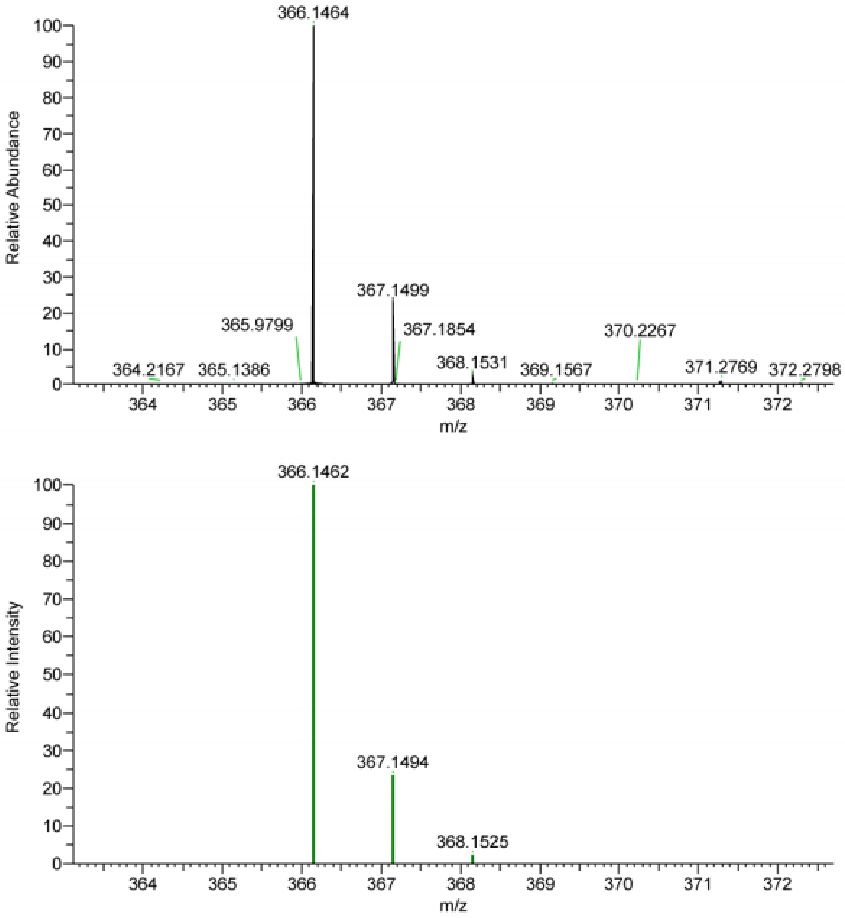

Figure S9. HRESI-MS of **1·HB^{Ph}**.

1·XB^{PFP}

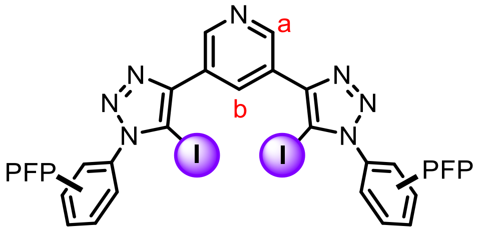

^{1}H NMR (400 MHz, $CDCl_3$) δ = 9.42 (s, $2H_a$), 9.11 (t, *J* = 2.1 Hz, $1H_b$).

^{13}C NMR (126 MHz, $CDCl_3$) δ = 148.48, 147.78, 143.77 (dm, *J* = 260 Hz), 138.20 (dm, *J*= 260 Hz), 133.12, 125.69, 112.24 (td, *J* = 14.6 Hz, *J* = 4.0 Hz,) 81.37. (Peak missing due to C-F coupling).

^{19}F NMR (376 MHz, $CDCl_3$) δ = -141.58 – -142.69 (m), -146.57 (tt, *J* = 21.4, 3.6 Hz), -158.00 – -159.39 (m).

HRMS (ESI +ve) m/z: 797.8443, ($[M+H]^+$, $C_{21}H_4N_7F_{10}I_2$ requires 797.8452).

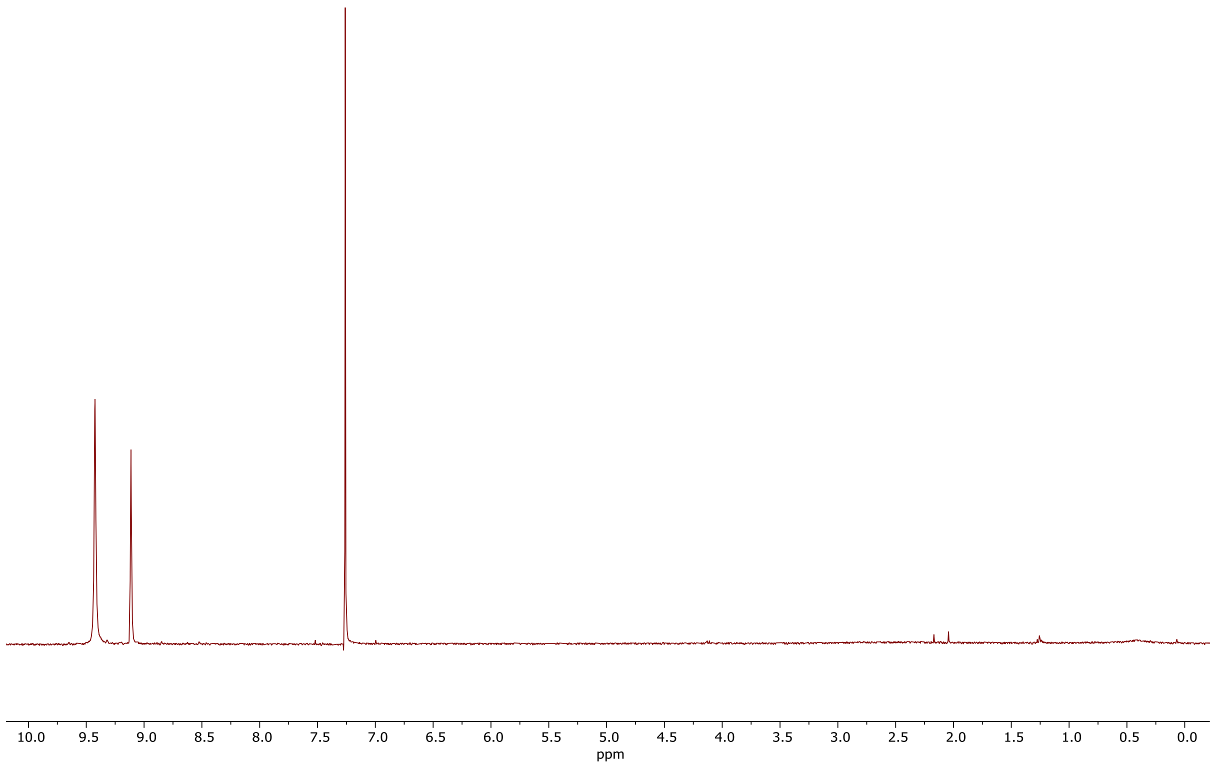

Figure S10. ^{1}H NMR spectrum of **1·XB^{PFP}** ($CDCl_3$, 400 MHz, 298 K).

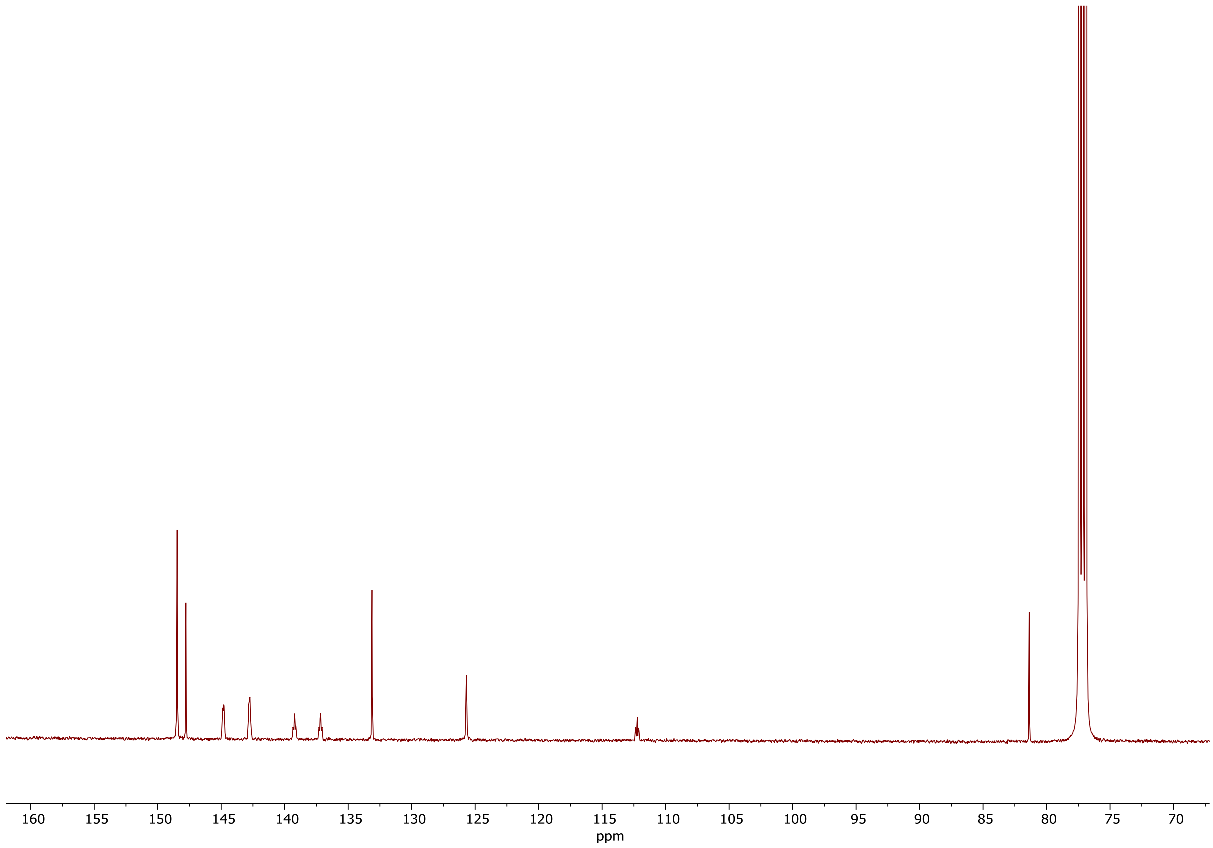

Figure S11. ^{13}C NMR spectrum of **1·XB^{PFP}** ($CDCl_3$, 400 MHz, 298 K).

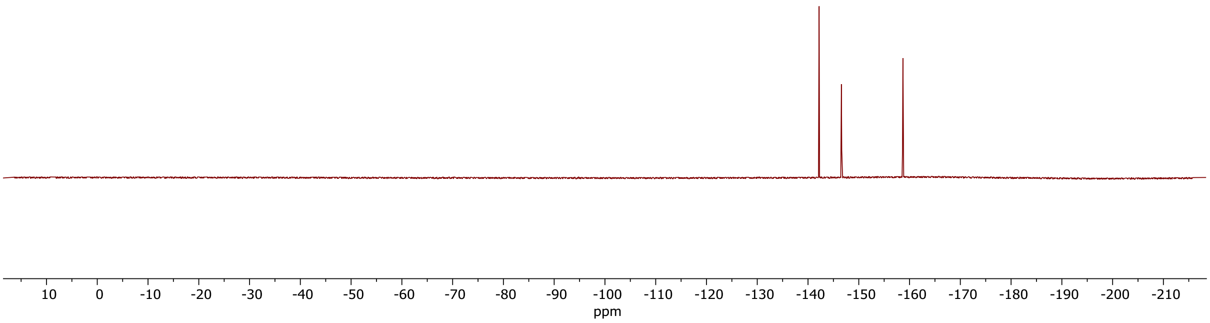

Figure S12. ^{19}F NMR spectrum of **1·XB^{PFP}** ($CDCl_3$, 400 MHz, 298 K).

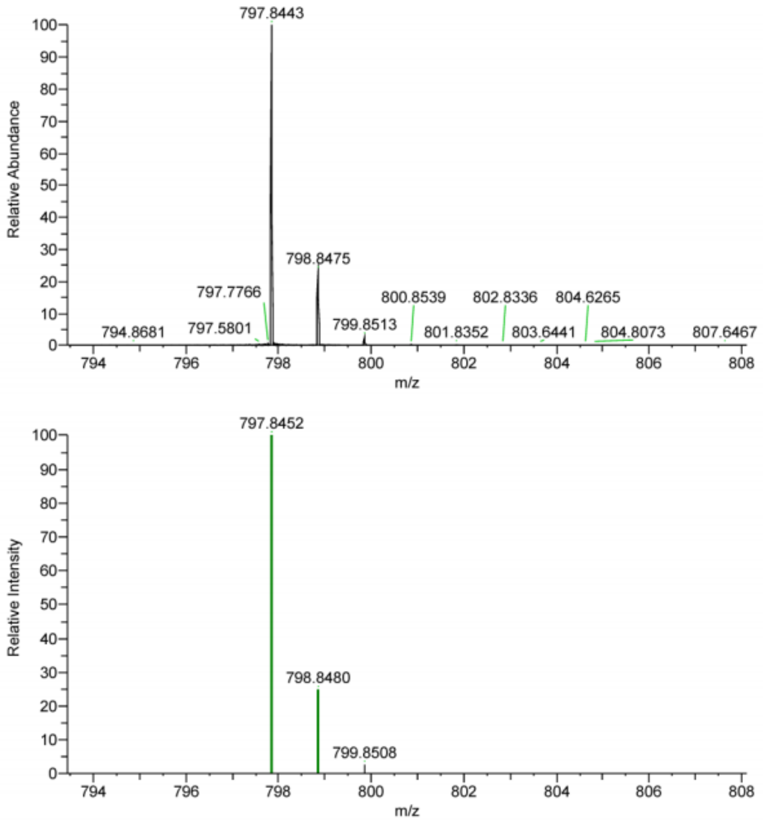

Figure S13. HRESI-MS of **1·XB**$^{\mathbf{PFP}}$.

1·ChB^{PFP}

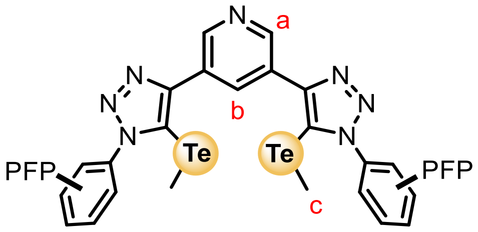

^{1}H NMR (400 MHz, $CDCl_3$) δ = 9.48 (d, J = 2.2 Hz, $2H_a$), 9.24 (s, $1H_b$), 2.06 (s, $6H_c$).

^{13}C NMR (126 MHz, DMSO) δ = 149.95, 148.06, 142.97 (dm, $^{1}J_{CF1or2or3}$ = 254 Hz), 137.68 (dm, $^{1}J_{CF1or2or3}$ = 256 Hz), 133.38, 126.55, 113.01 – 112.76 (m, 2C) 109.44, -11.37. (Peak missing due to C-F coupling).

^{19}F NMR (376 MHz, $CDCl_3$) δ = -143.00 – -143.18 (m), -147.83 (t, J = 21.3 Hz), -159.35 (tt, J = 21.0, 5.6 Hz).

HRMS (ESI +ve) m/z: 829.8922, ($[M+H]^+$, $C_{23}H_{10}N_7F_{10}Te_2$ requires 829.8721).

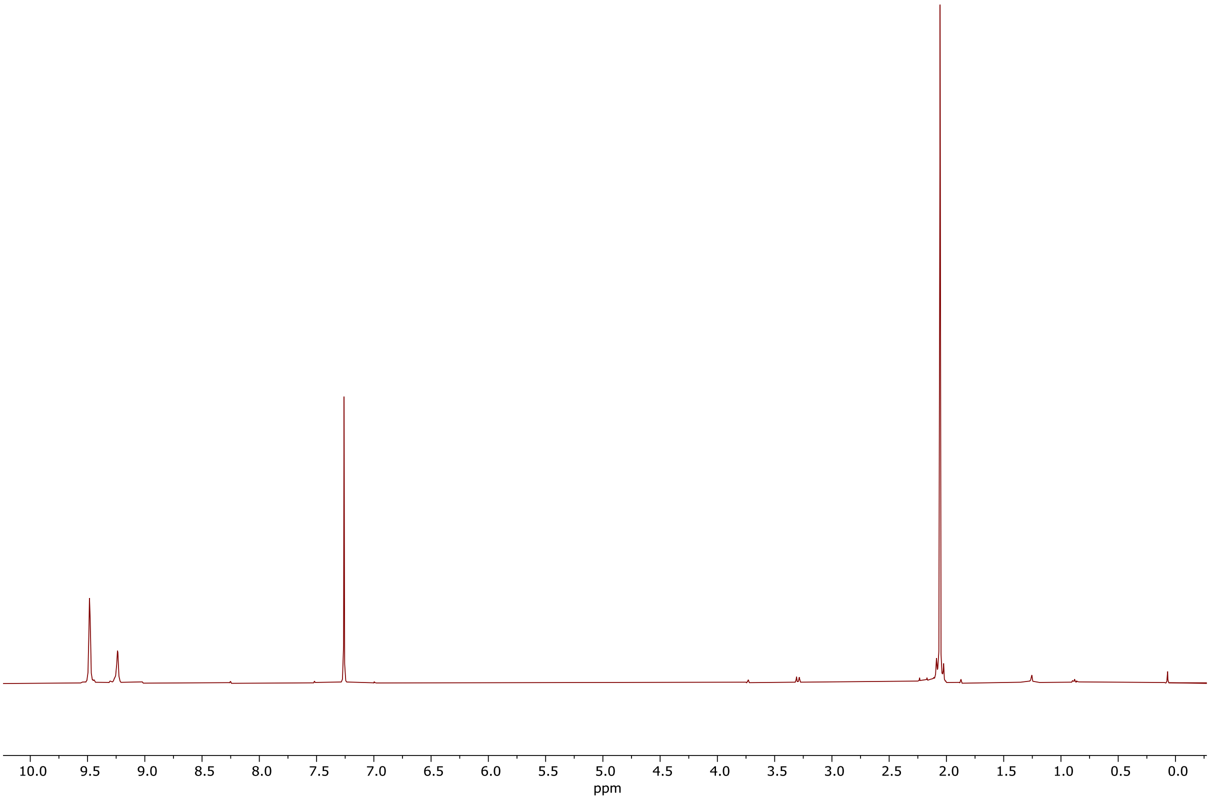

Figure S14. ^{1}H NMR spectrum of **1·ChB^{PFP}** ($CDCl_3$, 400 MHz, 298 K).

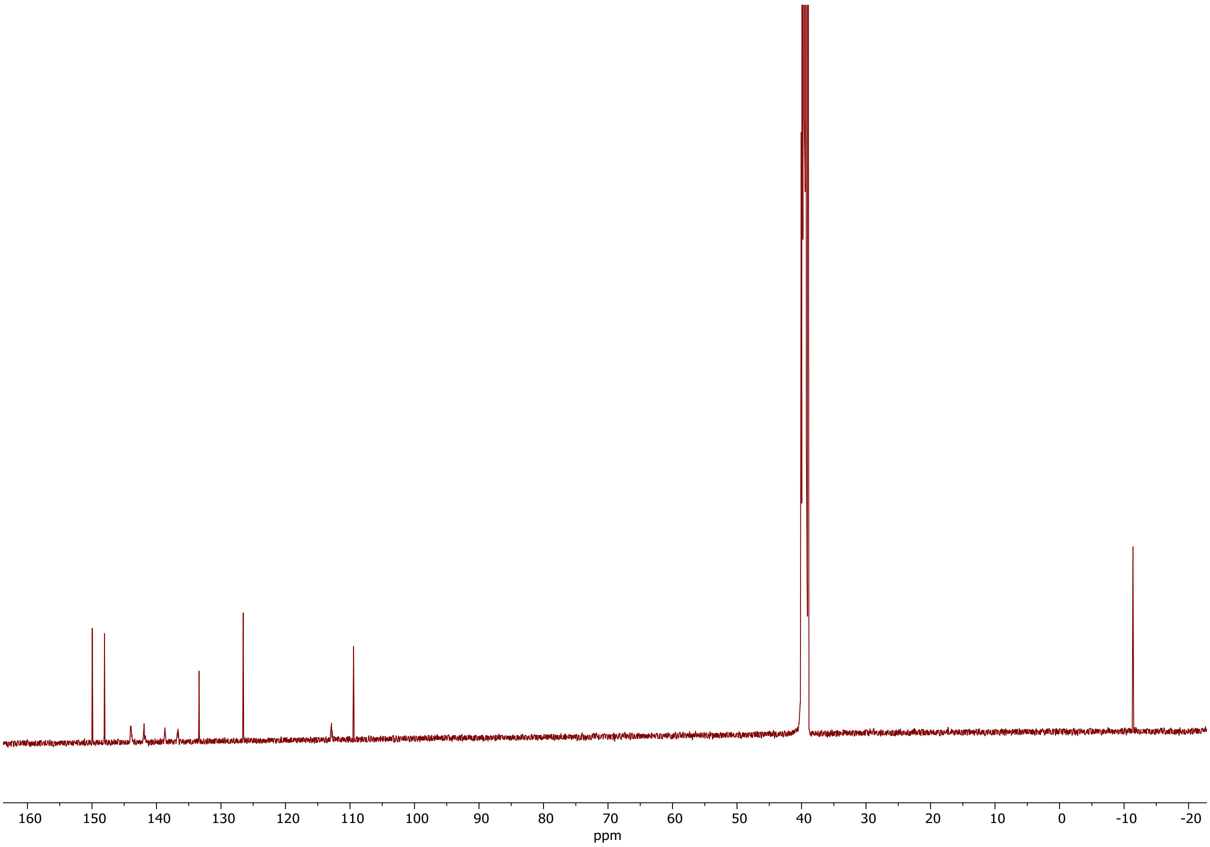

Figure S15. ^{13}C NMR spectrum of **1·ChB^{PFP}** ($CDCl_3$, 126 MHz, 298 K).

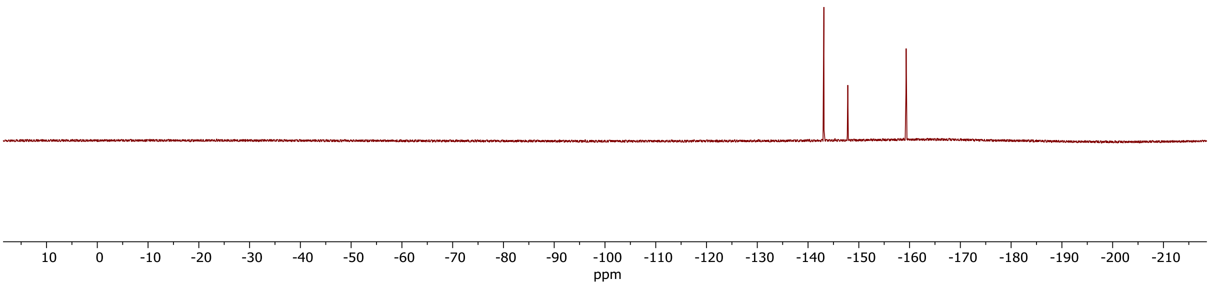

Figure S16. ^{19}F NMR spectrum of **1·ChB^{PFP}** ($CDCl_3$, 376 MHz, 298 K).

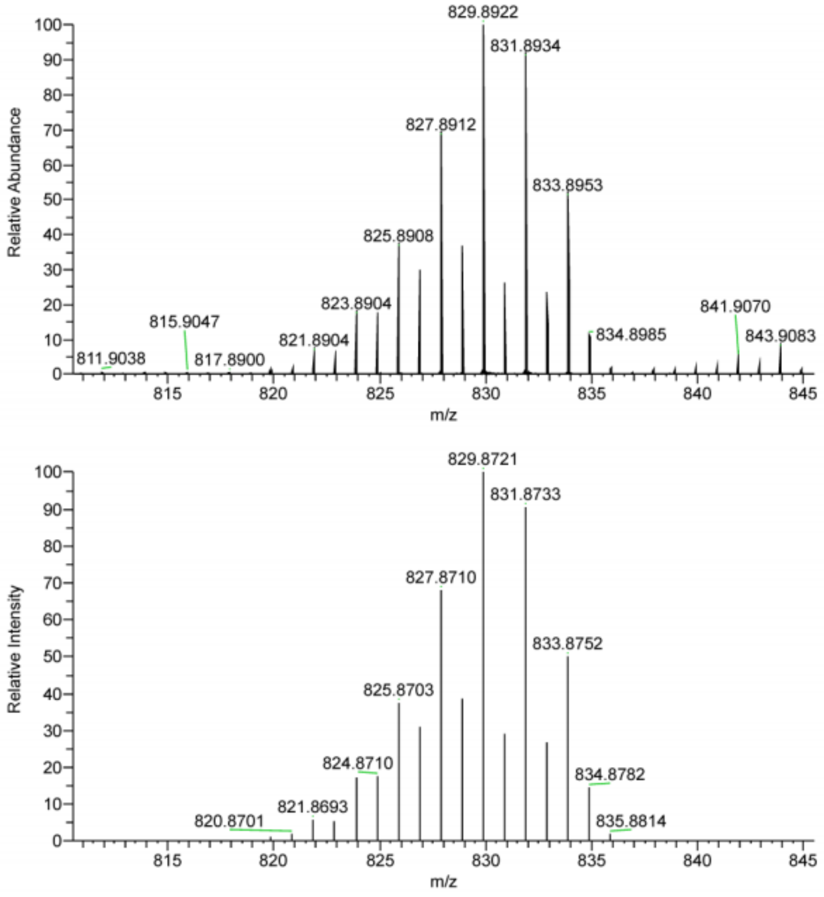

Figure S17. HRESI-MS of **1·ChB^{PFP}**.

1·HB^{PFP}

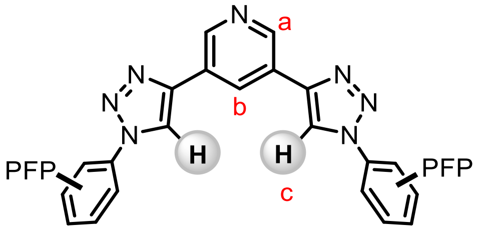

^{1}H NMR (400 MHz, $CDCl_3$) δ = 9.22 (s, $2H_a$), 8.87 (s, $1H_b$), 8.31 (s, $2H_c$).

^{13}C NMR (126 MHz, $CDCl_3$) δ = 147.23, 144.98, 142.64 (dm, *J* = 258 Hz) 138.33 (dm, *J* = 258 Hz), 130.69, 126.13, 122.89, 112.88 (td, *J* = 14.1 Hz, *J* = 4.1 Hz) (Peak missing due to C-F coupling).

^{19}F NMR (376 MHz, $CDCl_3$) δ = -145.17 – -145.32 (m), -148.89 – -149.05 (m), -158.70 – -158.91 (m).

HRMS (ESI +ve) m/z: 546.0517, ($[M+H]^+$, $C_{21}H_6N_7F_{10}$ requires 546.0520).

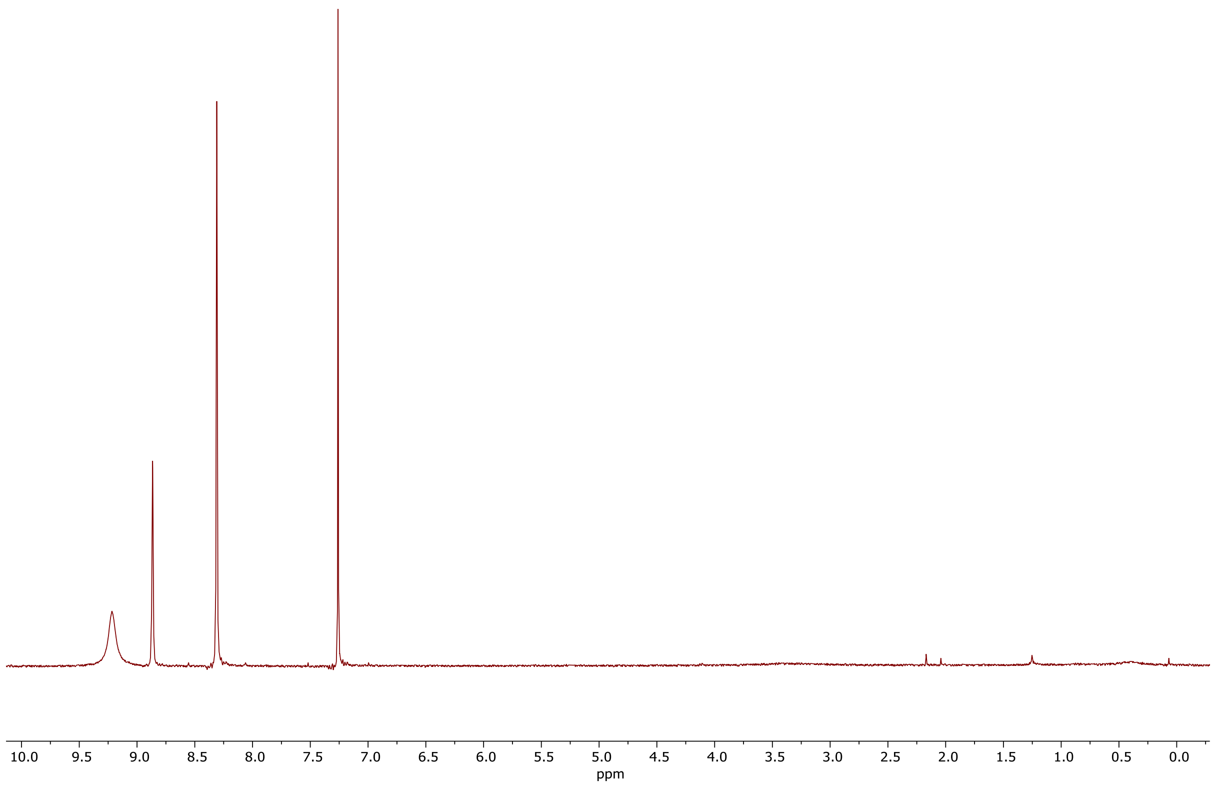

Figure S18. ^{1}H NMR spectrum of **1·HB^{PFP}** (CDCl$_3$, 400 MHz, 298 K).

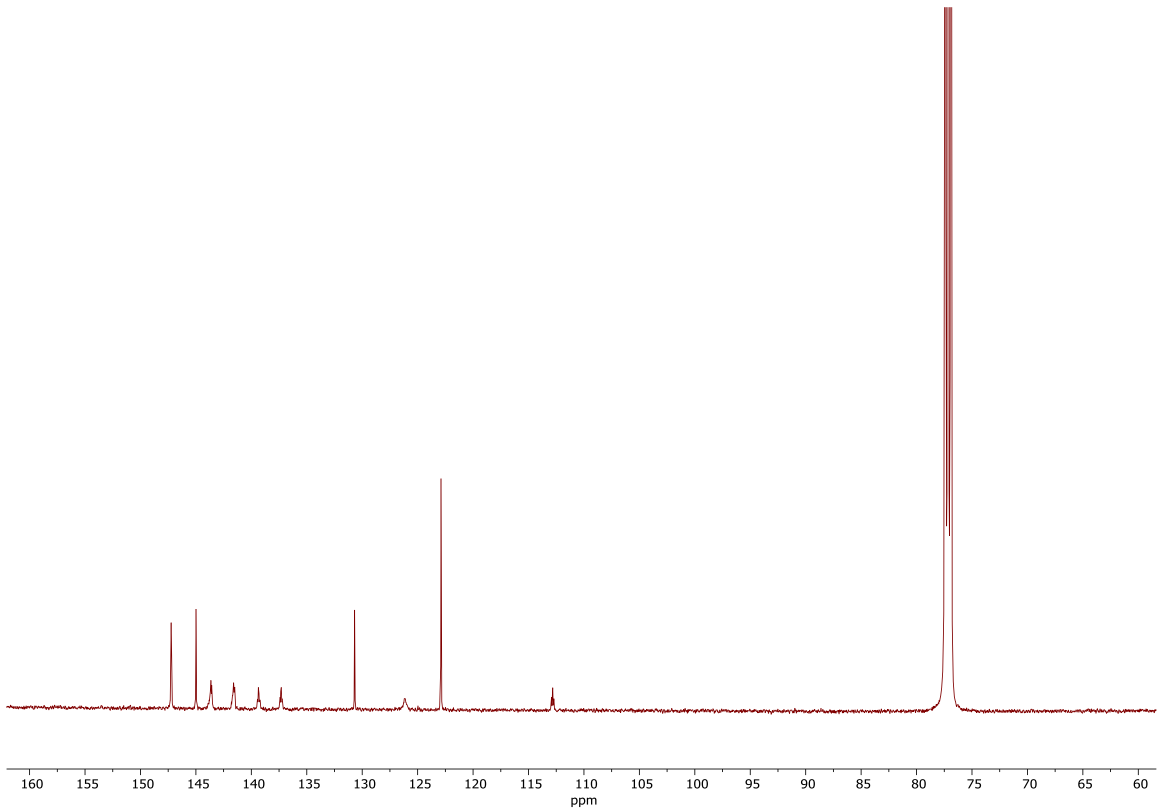

Figure S19. ^{13}C NMR spectrum of **1·HB^{PFP}** (CDCl$_3$, 126 MHz, 298 K).

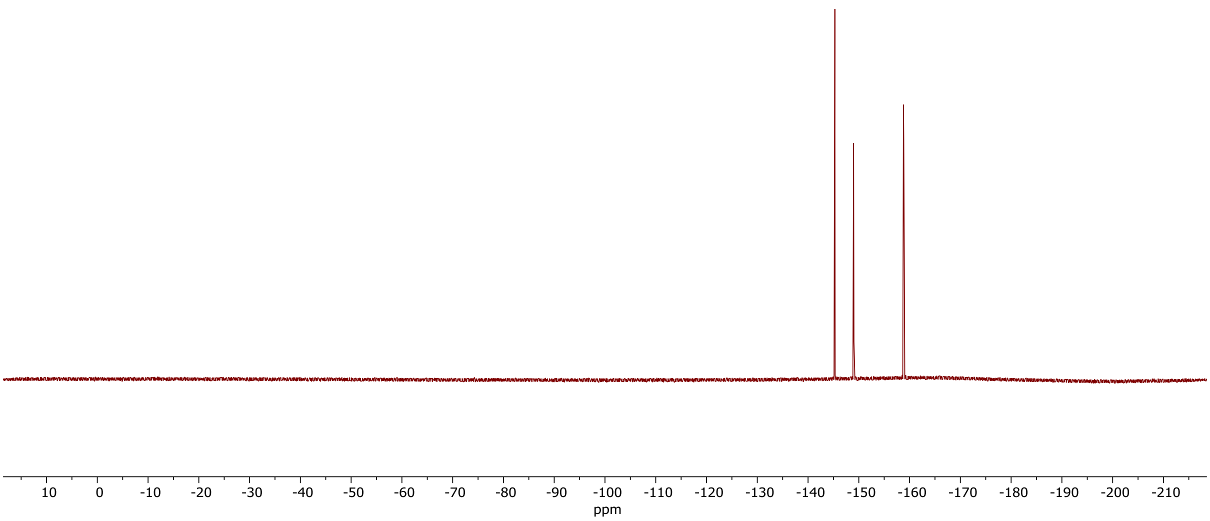

Figure S20. ^{19}F NMR spectrum of **1·HB^{PFP}** ($CDCl_3$, 376 MHz, 298 K).

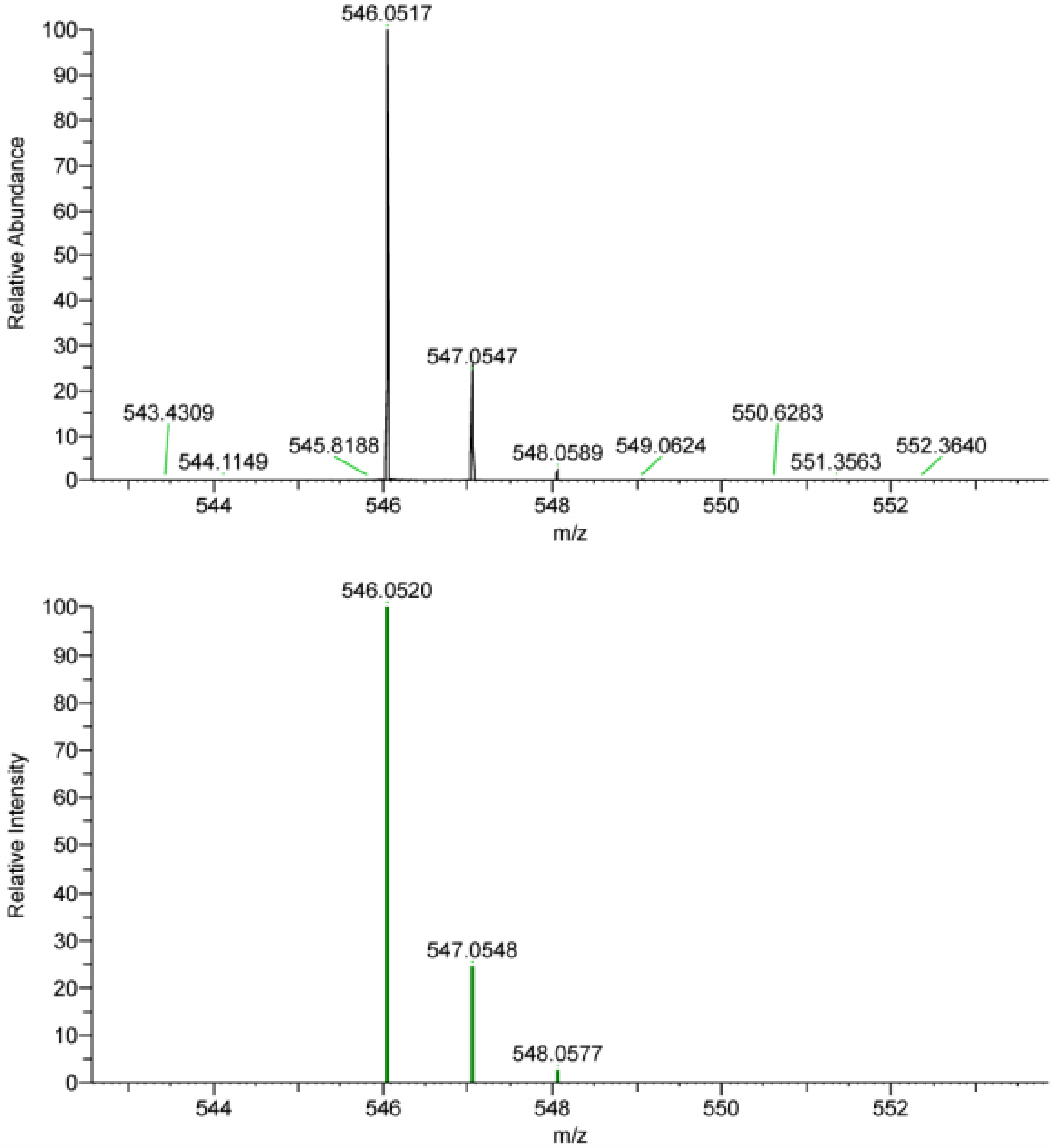

Figure S21. HRESI-MS of **1·HB**$^{\mathbf{PFP}}$.

2·ChB^{mF}

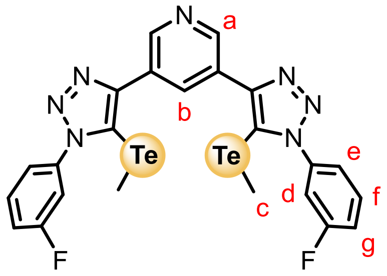

^{1}H NMR (400 MHz, DMSO) δ 9.24 (d, *J* = 2.2 Hz, 2H$_a$), 8.99 (t, *J* = 2.2 Hz, 1H$_b$), 7.81 – 7.35 (m, 8H$_{d,e,f,g}$), 1.78 (s, 6H$_c$).

^{13}C NMR (101 MHz, DMSO) δ 162.92, 160.48, 149.64,147.90, 138.98 (m), 131.05 (m), 127.28, 122.96, 116.97 (m), 114.30 (m), 105.90, -11.36

^{19}F NMR (377 MHz, DMSO) δ -106.50.

HRMS (ESI +ve) m/z: 685.9679, ([M+H]$^+$, $C_{23}H_{18}N_7F_2Te_2$ requires 685.9682).

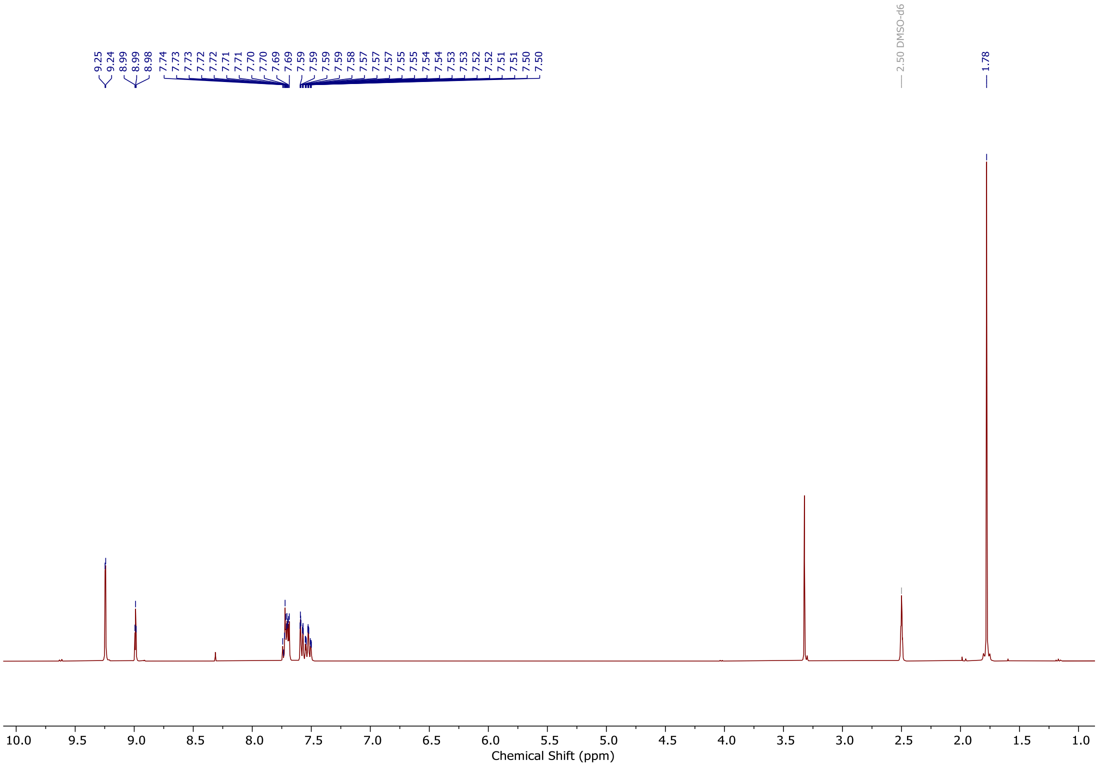

Figure S22. ^{1}H NMR spectrum of **2·ChB^{mF}** (DMSO, 400 MHz, 298 K).

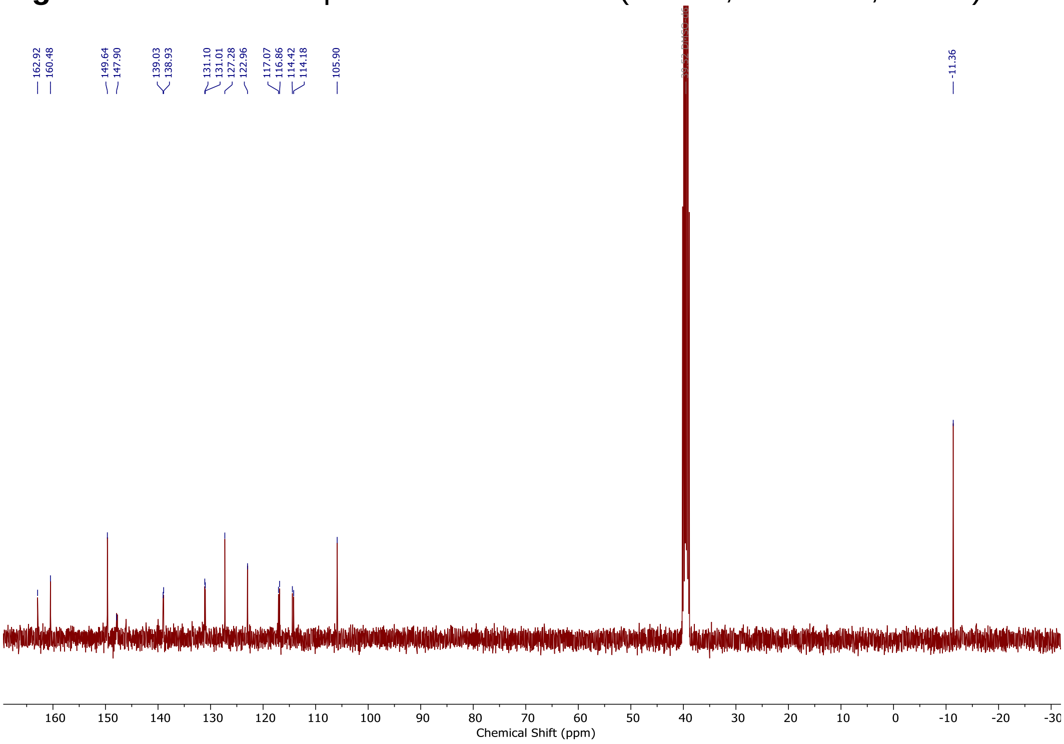

Figure S23. ^{13}C NMR spectrum of **2·ChB^{mF}** (DMSO, 101 MHz, 298 K).

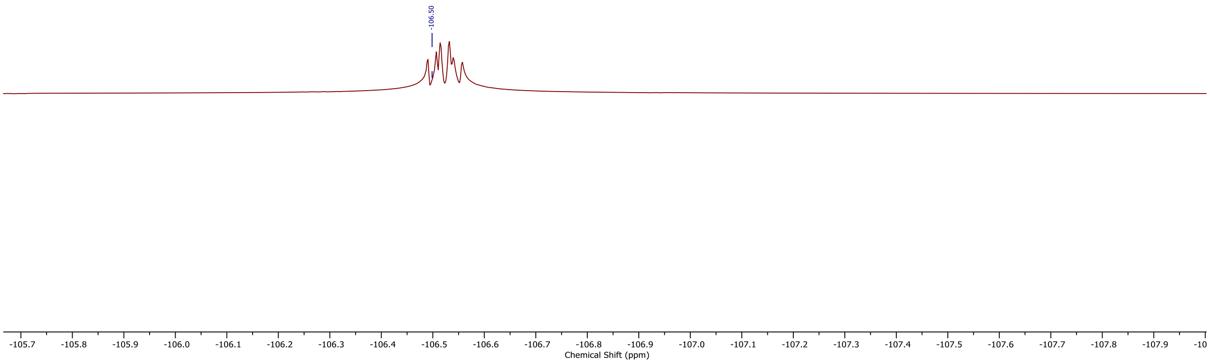

Figure S24. ^{19}F NMR spectrum of **2·ChB$^{\mathbf{mF}}$** (DMSO, 377 MHz, 298 K).

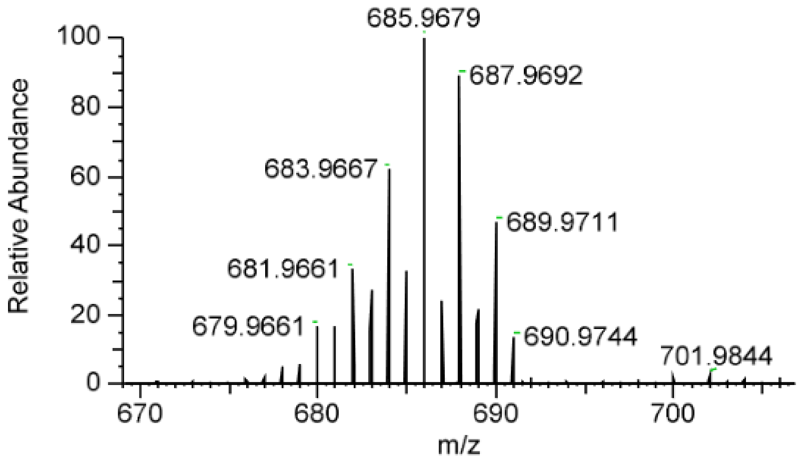

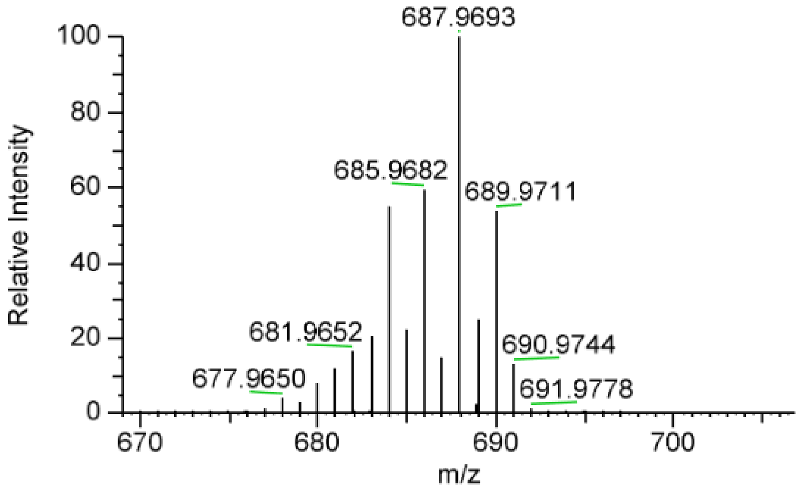

Figure S25. HRESI-MS of **2·ChB^{mF}**.

2·ChB^{2F}

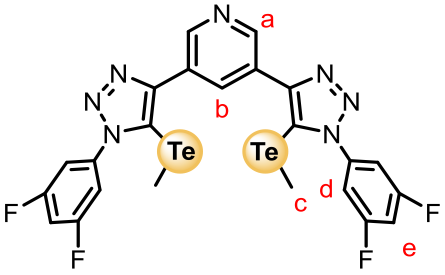

^{1}H NMR (400 MHz, CDCl$_3$) δ 9.26 (d, *J* = 2.2 Hz, 2H$_a$), 9.00 (t, *J* = 2.2 Hz, 1H$_b$), 7.67 – 7.57 (m, 6H$_{d,e}$), 1.80 (s, 6H$_c$).

^{19}F NMR (377 MHz, CDCl3) δ -102.65 – -103.94 (m).

HRMS (ESI +ve) m/z: 721.9489, ([M+H]$^+$, $C_{23}H_{16}N_7F_4Te_2$ requires 721.9493).

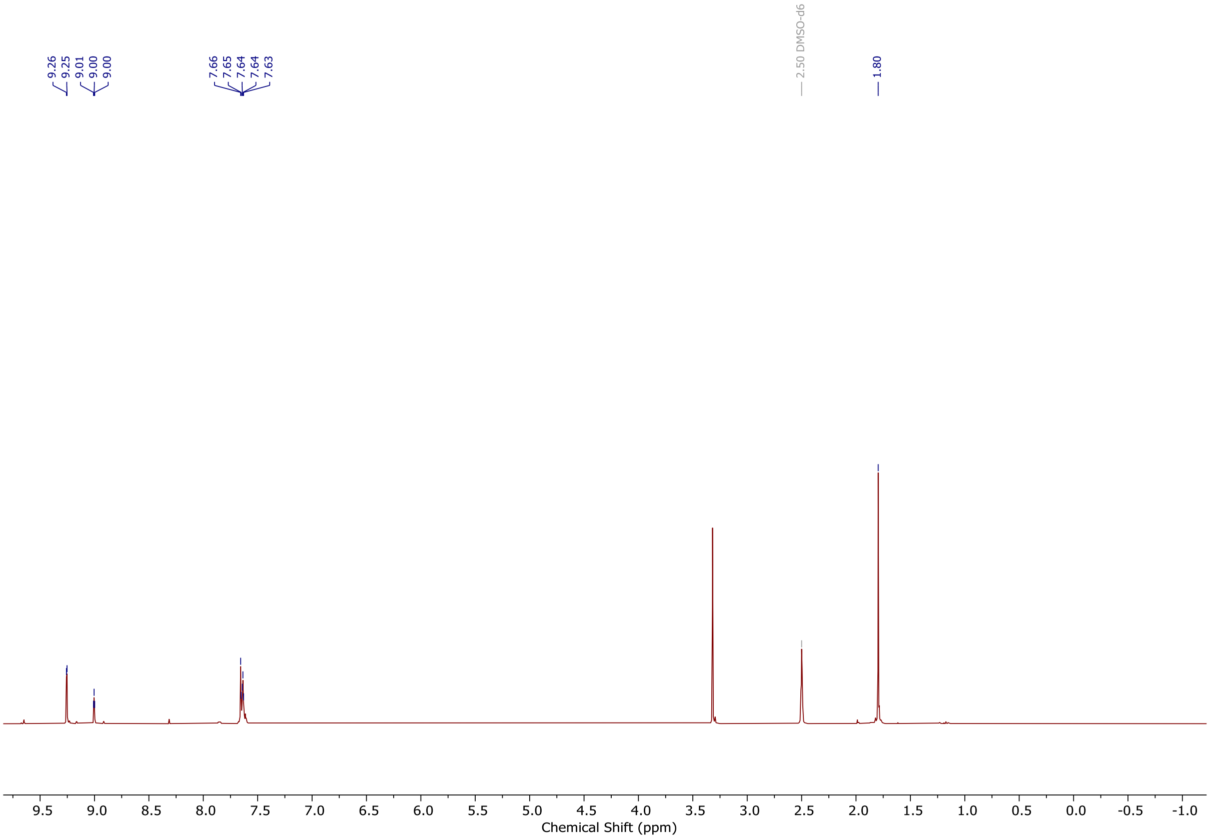

Figure S26. ^{1}H NMR spectrum of **2·ChB^{2F}**($CDCl_3$, 400 MHz, 298 K).

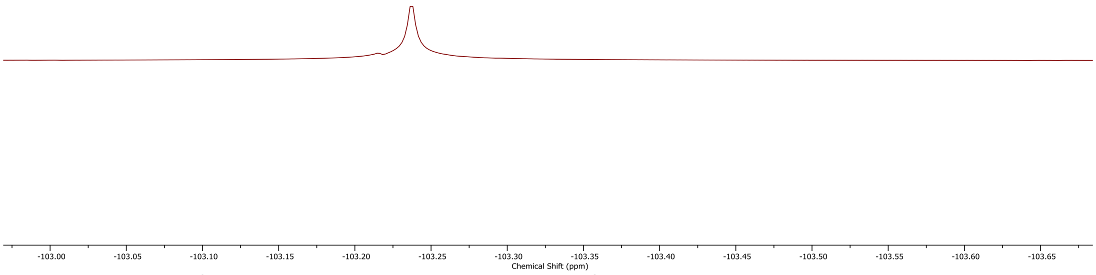

Figure S27. ^{19}F NMR spectrum of **2·ChB^{2F}**(CDCl$_3$, 377 MHz, 298 K).

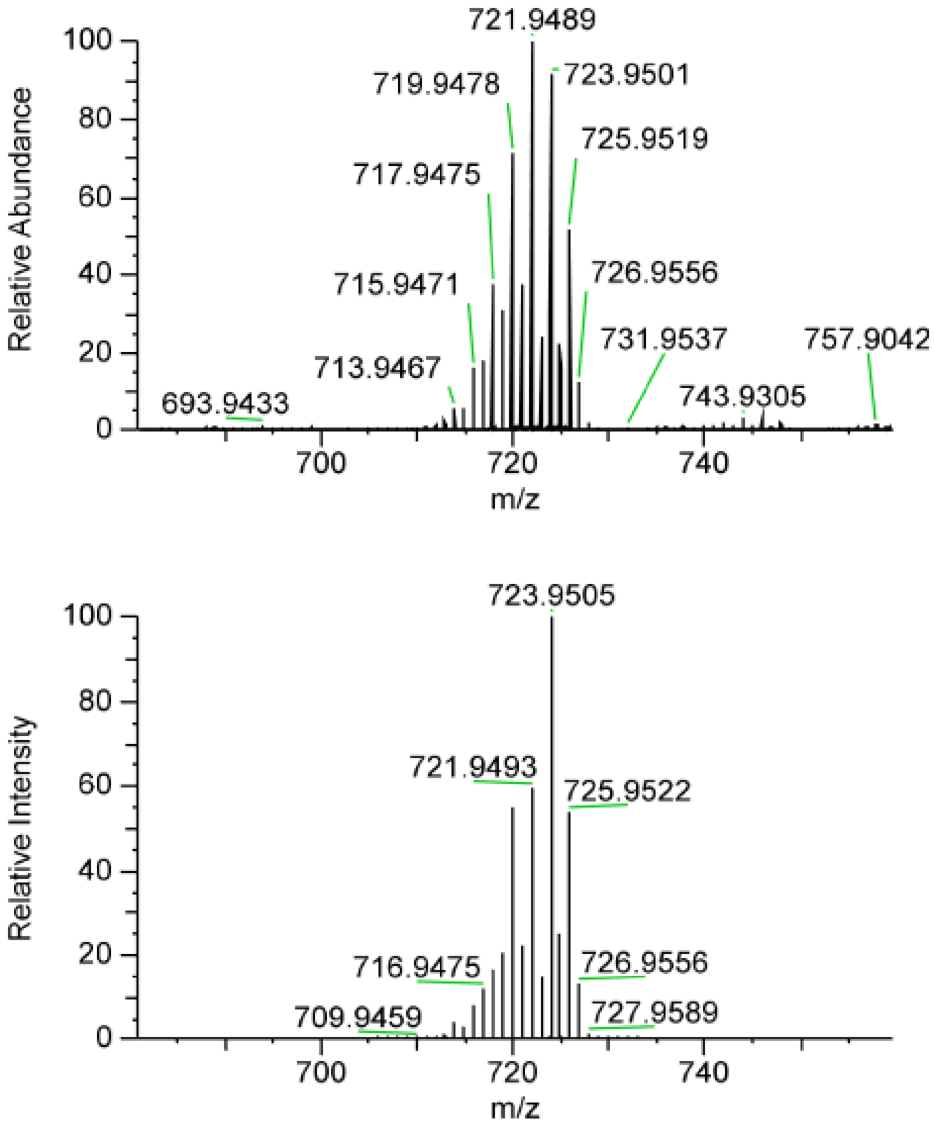

Figure S28. HRESI-MS of **2·ChB**^{2F}.

2·ChB^{3F}

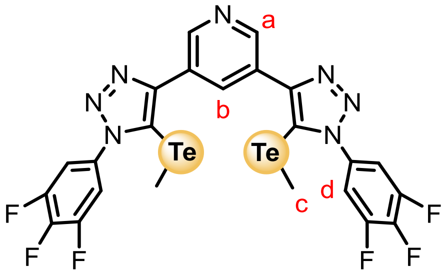

^{1}H NMR (500 MHz, DMSO) δ 9.26 (d, J = 2.1 Hz, 2H$_a$), 9.01 (t, J = 2.1 Hz, 1H$_b$), 7.94 (dd, J = 8.1, 6.2 Hz, 4H$_d$), 1.80 (s, 6H$_c$).

^{13}C NMR (126 MHz, DMSO) δ 149.98 (dm, J = 257 Hz), 149.59, 147.80, 141.03, 133.23, 127.17, 113.28 – 112.65 (m), 106.65, -11.15.

^{19}F NMR (470 MHz, DMSO) δ -133.19 (d, J = 22.4 Hz), -158.26 (t, J = 22.4 Hz).

HRMS (ESI +ve) m/z: 759.9309, ([M+H]$^+$, $C_{23}H_{14}F_6N_7Te_2$ requires 759.9316).

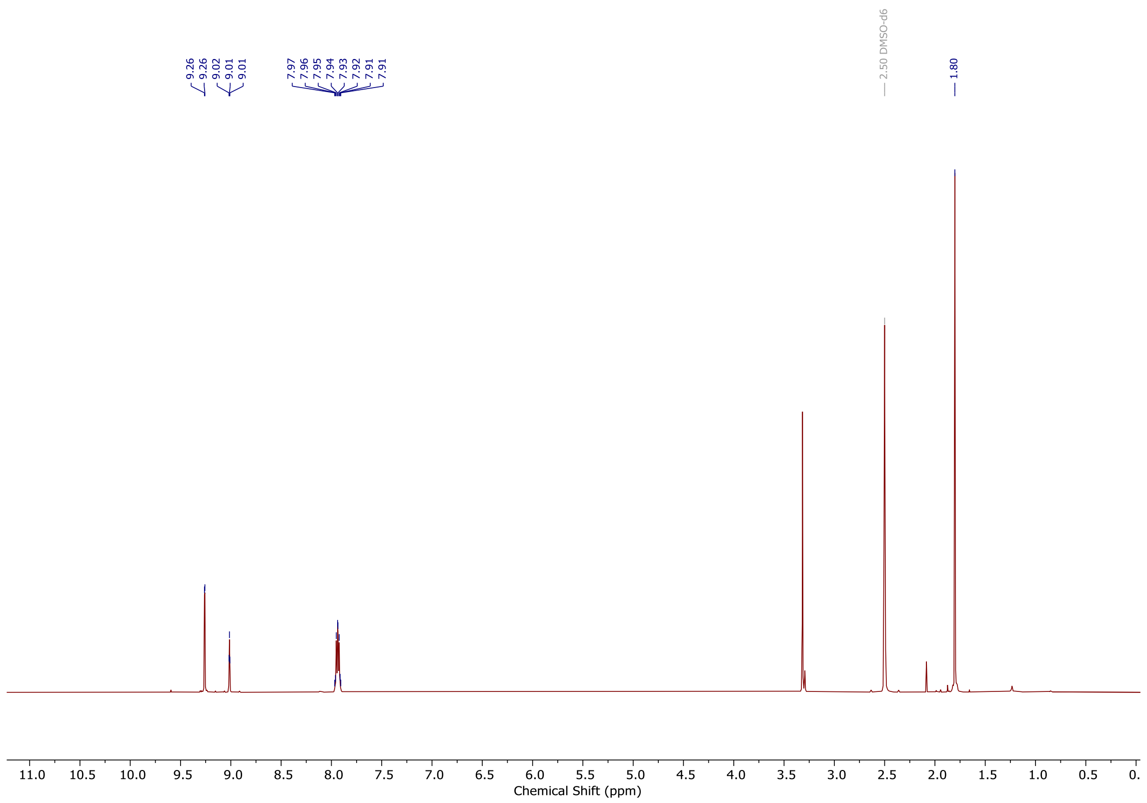

Figure S29. ^{1}H NMR spectrum of **2·ChB^{3F}** (DMSO, 500 MHz, 298 K).

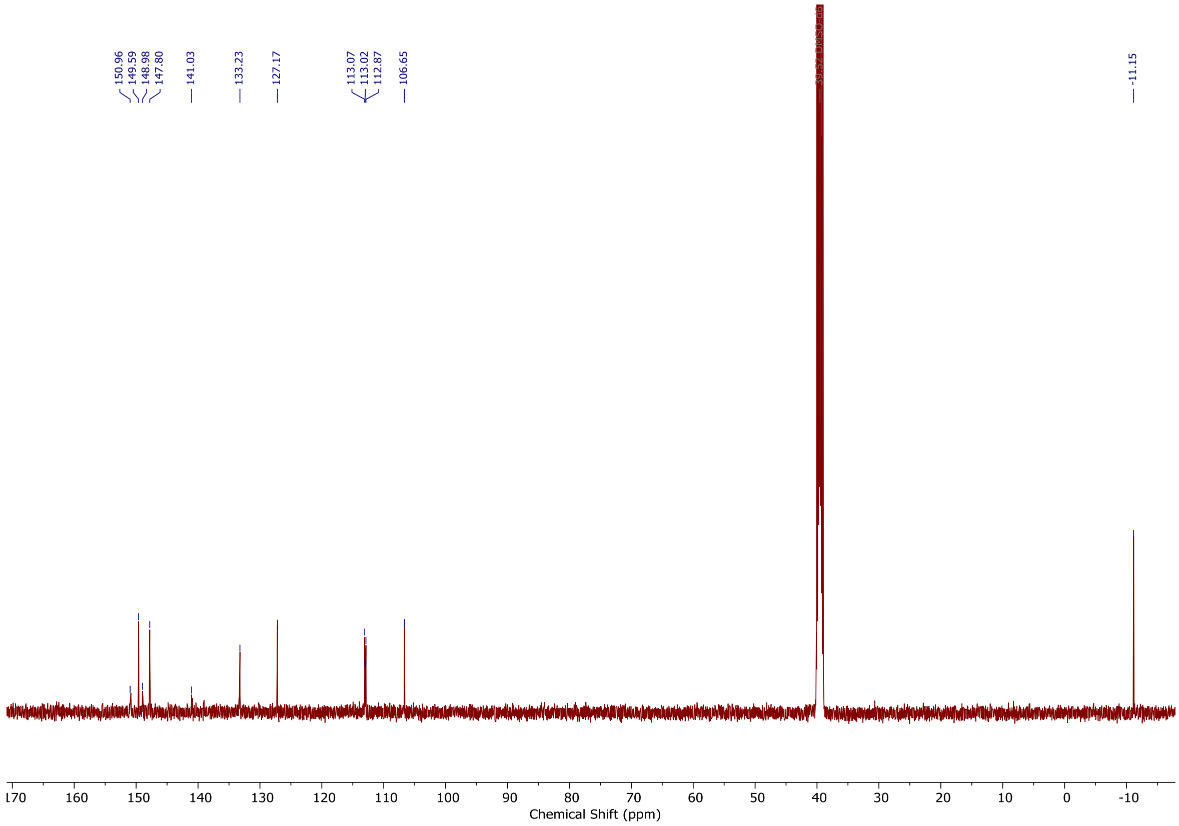

Figure S30. ^{13}C NMR spectrum of **2·ChB^{3F}** (DMSO, 126 MHz, 298 K).

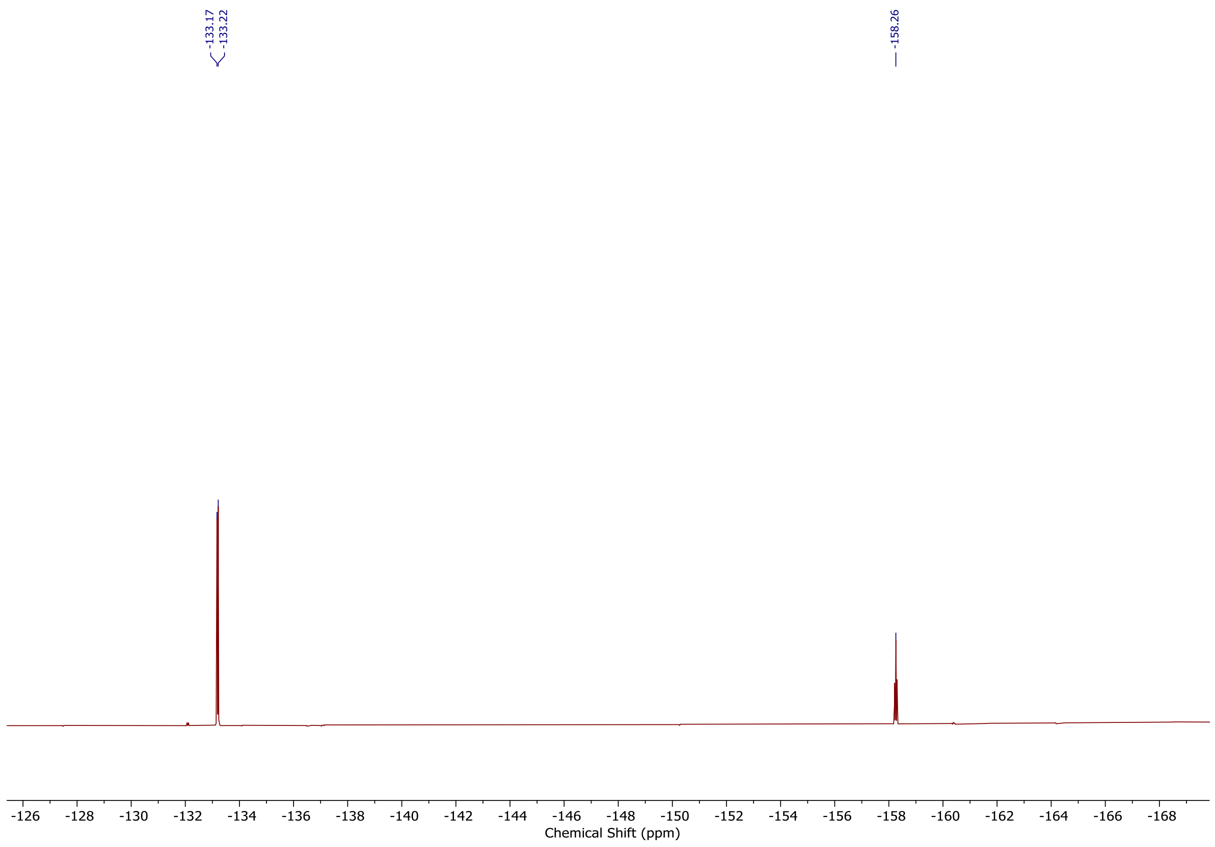

Figure S31. ^{19}F NMR spectrum of **2·ChB^{3F}** (DMSO, 470 MHz, 298 K).

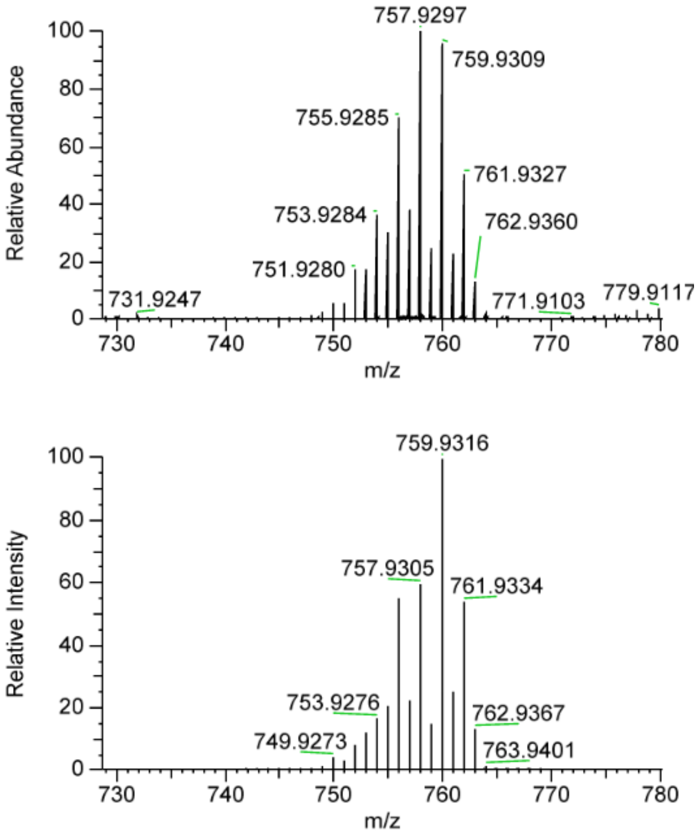

Figure S32. HRESI-MS of **2·ChB^{3F}**.

3·ChB^{pOMe}

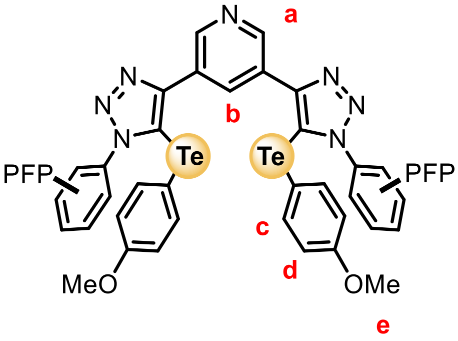

^{1}H NMR (400 MHz, $CDCl_3$) δ 9.28 (d, J = 2.2 Hz, $2H_a$), 8.87 (t, J = 2.2 Hz, $1H_b$), 7.42 – 7.32 (m, $4H_c$), 6.71 – 6.55 (m, $4H_d$), 3.75 (s, $6H_e$).

^{13}C NMR (151 MHz, $CDCl_3$) δ 161.23, 150.68, 149.04, 144.07 (d, J = 12.9 Hz), 143.76 (dm, J = 261 Hz), 143.19 (dm, J = 267 Hz), 142.30 (t, J = 13.5 Hz), 141.01, 138.81 (d, J = 13.8 Hz), 137.96 (dm, J = 257 Hz), 137.11 (d, J = 13.5 Hz), 134.85, 126.61, 116.02, 113.53 (t, J = 13.9 Hz), 107.19, 100.34, 55.42.

^{19}F NMR (377 MHz, $CDCl_3$) δ -141.04 – -143.08 (m), -148.68 (td, J = 21.6, 3.1 Hz), -160.01.

HRMS (ESI +ve) m/z: 1013.9451, ([M+H]$^+$, $C_{35}H_{17}N_7F_{10}O_2Te_2$ requires 1013.9452).

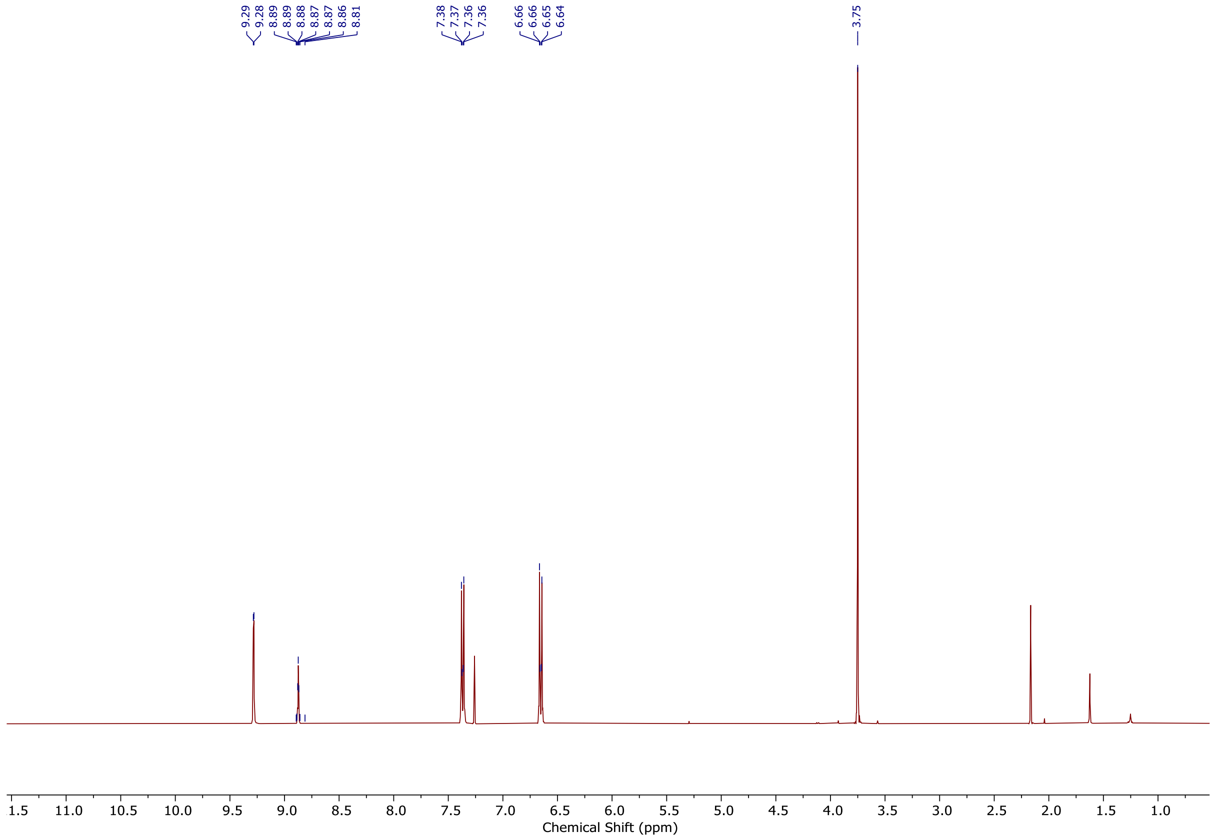

Figure S33. ^{1}H NMR spectrum of **3·ChB^{pOMe}** ($CDCl_3$, 400 MHz, 298 K).

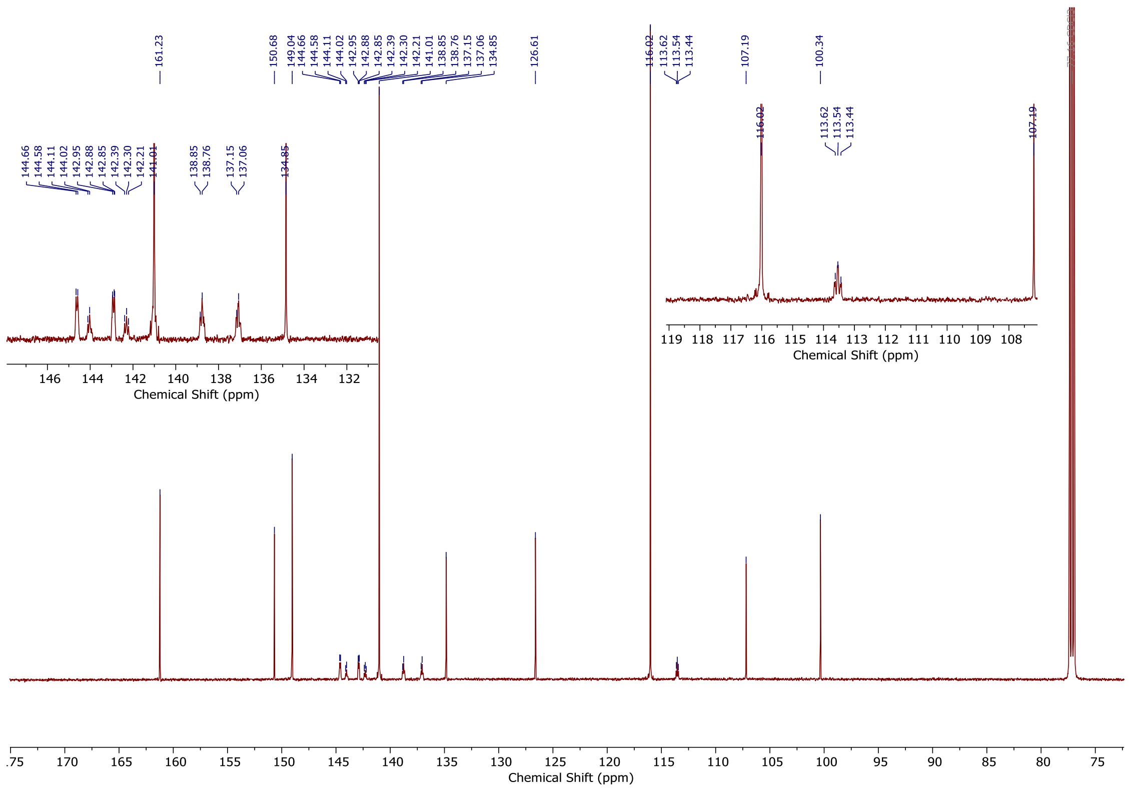

Figure S34. ^{13}C NMR spectrum of **3·ChB^{pOMe}** ($CDCl_3$, 151 MHz, 298 K).

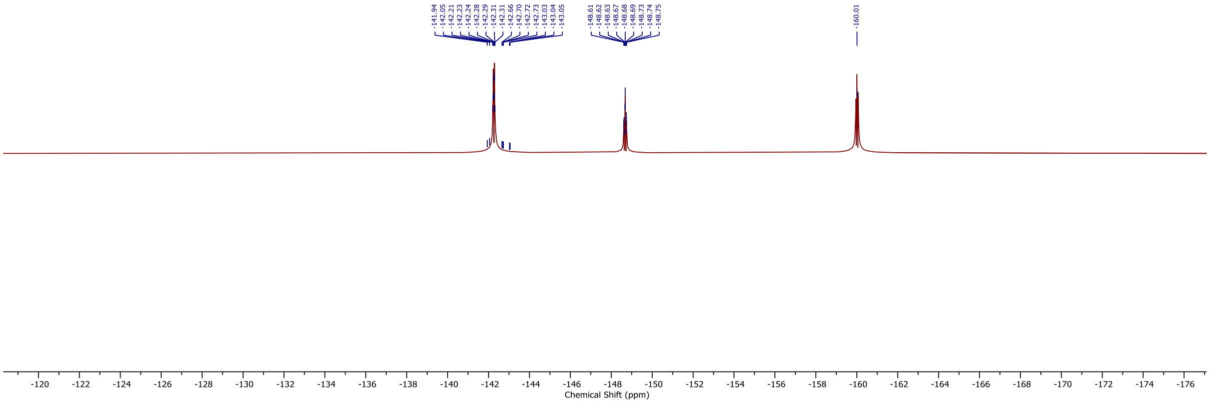

Figure S35. ^{19}F NMR spectrum of **3·ChB^{pOMe}** ($CDCl_3$, 377 MHz, 298 K).

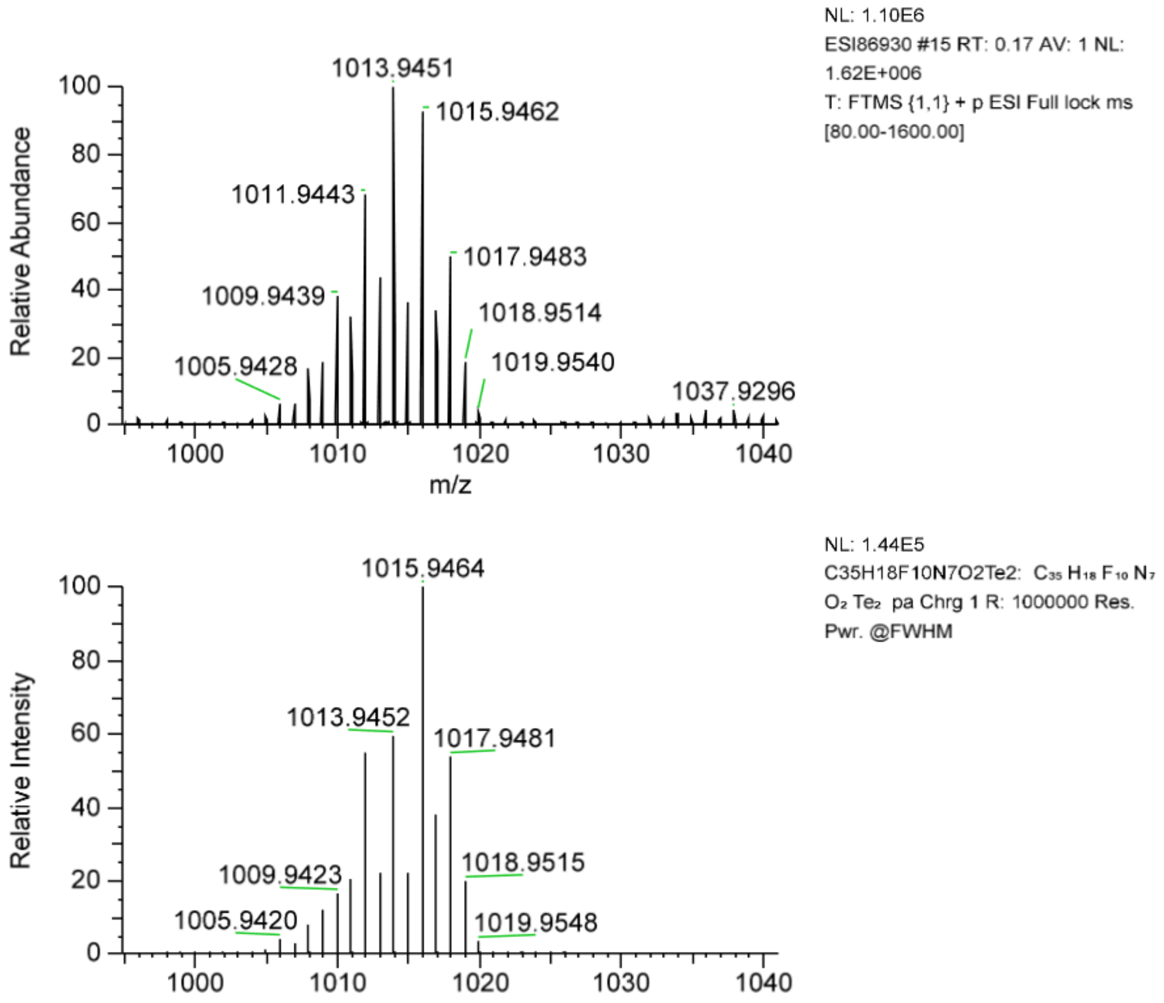

Figure S36. HRESI-MS of **3·ChB^{pOMe}**.

3·ChB^{pMe}

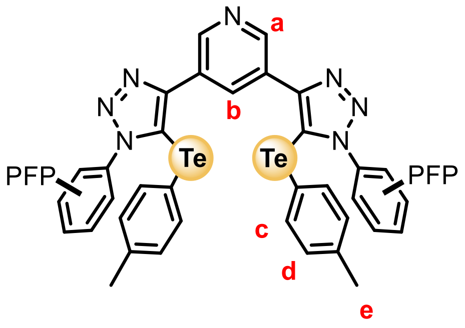

^{1}H NMR (400 MHz, $CDCl_3$) δ 9.28 (d, *J* = 2.1 Hz, $2H_a$), 8.87 (t, *J* = 2.1 Hz, $1H_b$), 7.37 – 7.27 (m, $4H_c$), 7.02 – 6.81 (m, $4H_d$), 2.30 (s, $6H_e$).

^{13}C NMR (151 MHz, $CDCl_3$) δ 150.89, 149.06, 143.72 (dm, *J* = 260 Hz), 143.18 (dm, *J* = 260 Hz), 140.40, 138.73, 137.08 (m), 134.78, 130.98, 126.57, 113.48 (t, *J* = 13.9 Hz), 107.43, 106.97, 21.29.

^{19}F-NMR (377 MHz, $CDCl_3$) δ = -142.12 – -142.29 (m), -148.38 (dd, *J* = 21.8, 3.2 Hz), -159.56 – -159.85 (m).

HRMS (ESI +ve) m/z: 983.9568, ($[M+H]^+$, $C_{35}H_{18}N_7F_{10}Te_2$ requires 983.9571).

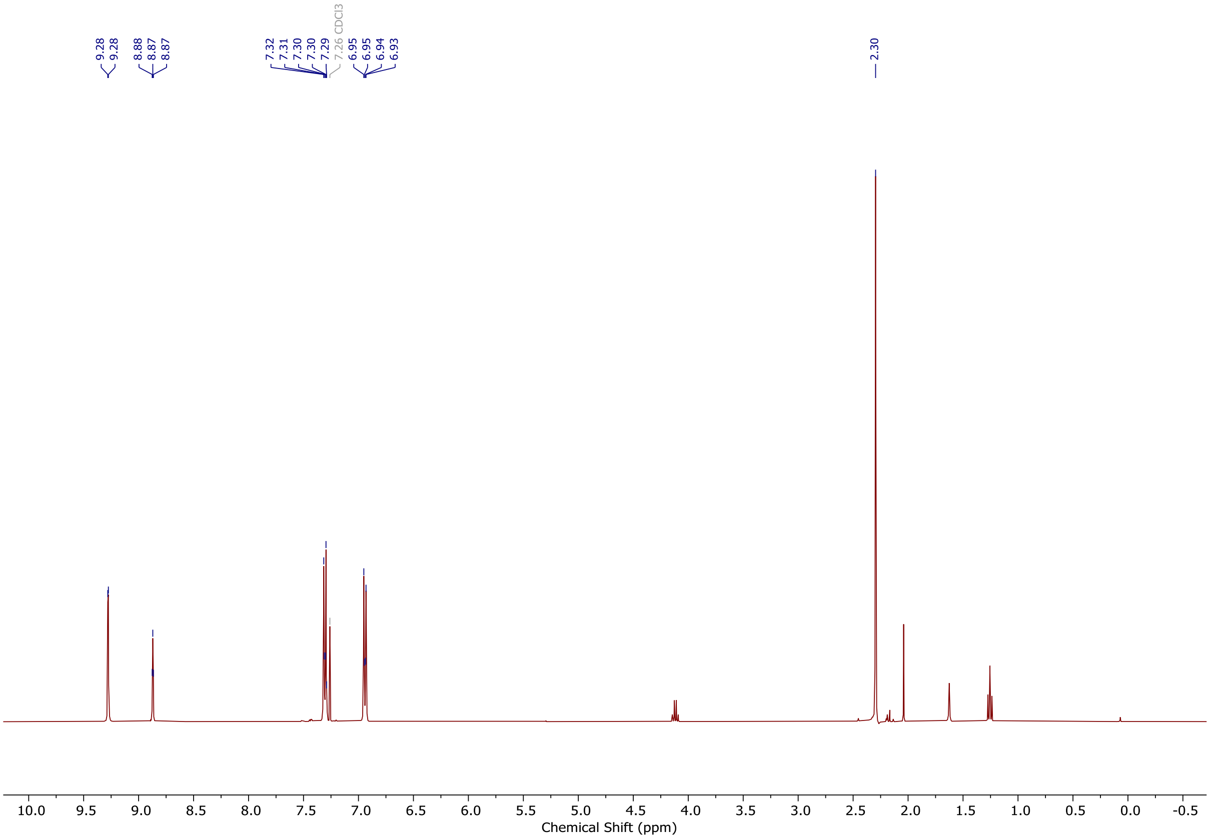

Figure S37. ^{1}H NMR spectrum of **3·ChB^{pMe}** ($CDCl_3$, 400 MHz, 298 K).

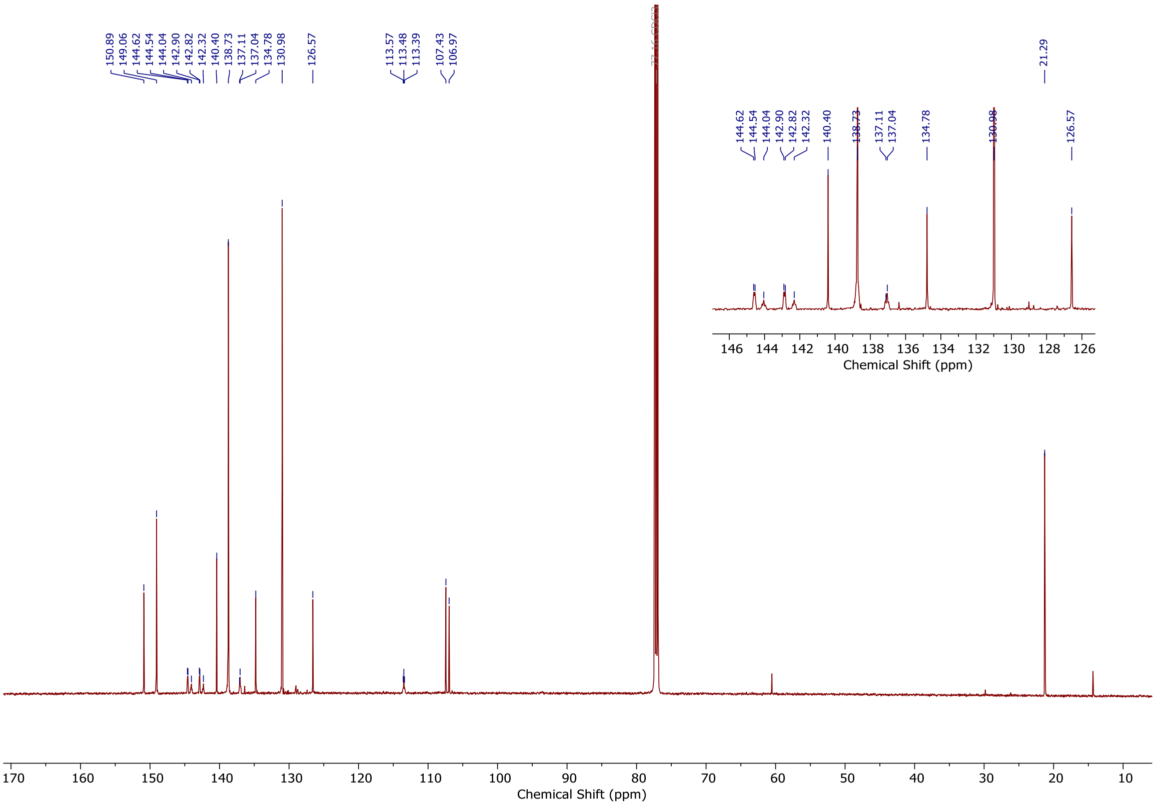

Figure S38. ^{1}H NMR spectrum of **3·ChB^{pMe}** ($CDCl_3$, 151 MHz, 298 K).

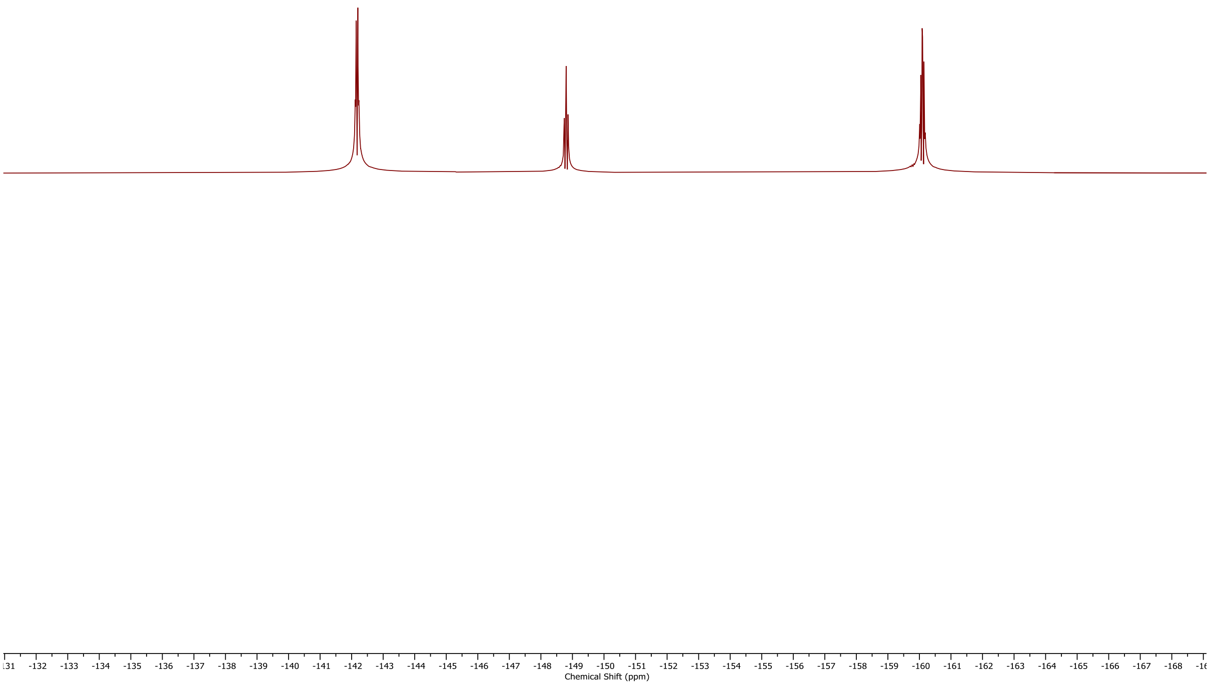

Figure S39. ^{19}F NMR spectrum of **3·ChB^{pMe}** ($CDCl_3$, 377 MHz, 298 K).

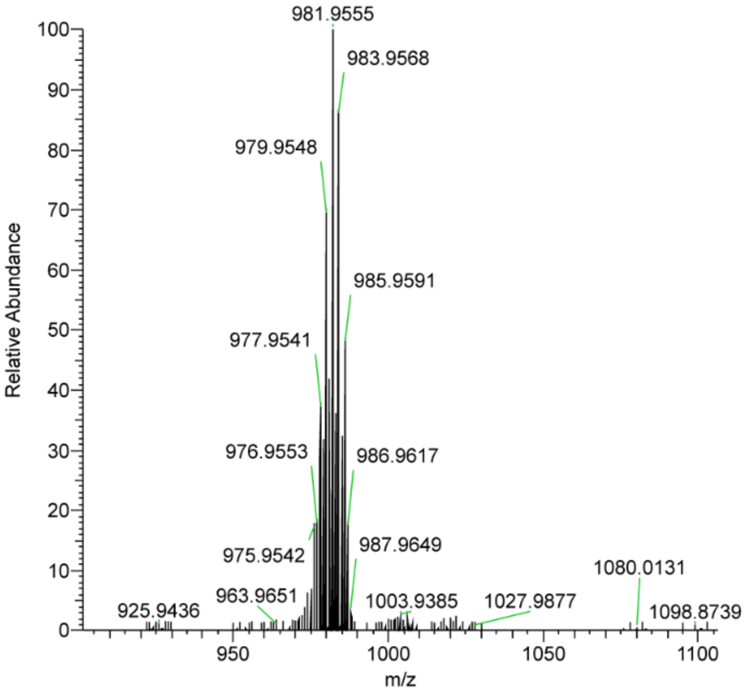

Figure S40. ^{1}H NMR spectrum of **3·ChB^{pMe}**.

3·ChB[pCl]

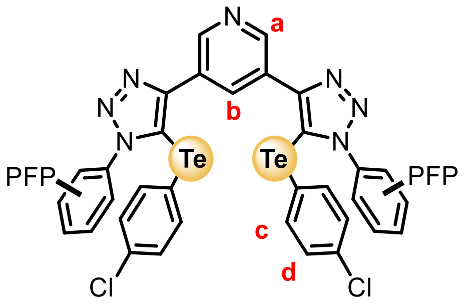

^{1}H NMR (400 MHz, $CDCl_3$) δ 9.29 (d, *J* = 2.1 Hz, $2H_a$), 8.89 (t, *J* = 2.2 Hz, $1H_b$), 7.36 – 7.28 (m, $2H_c$), 7.20 – 7.05 (m, $2H_d$).

^{13}C NMR (151 MHz, $CDCl_3$) δ 151.33, 149.11, 143.76, (dm, *J* = 254 Hz), 143.36, (dm, *J* = 261 Hz), 139.24, 138.21 (dm, *J* = 261 Hz), 136.59, 134.81, 130.52, 126.38, 109.31, 106.81.

^{19}F NMR (377 MHz, $CDCl_3$) δ -141.62 – -143.23 (m), -148.81 (td, *J* = 21.5, 3.1 Hz).

HRMS (ESI +ve) m/z: 1023.8447, ($[M+H]^+$, $C_{33}H_{12}Cl_2N_7F_{10}Te_2$ requires 1023.8473).

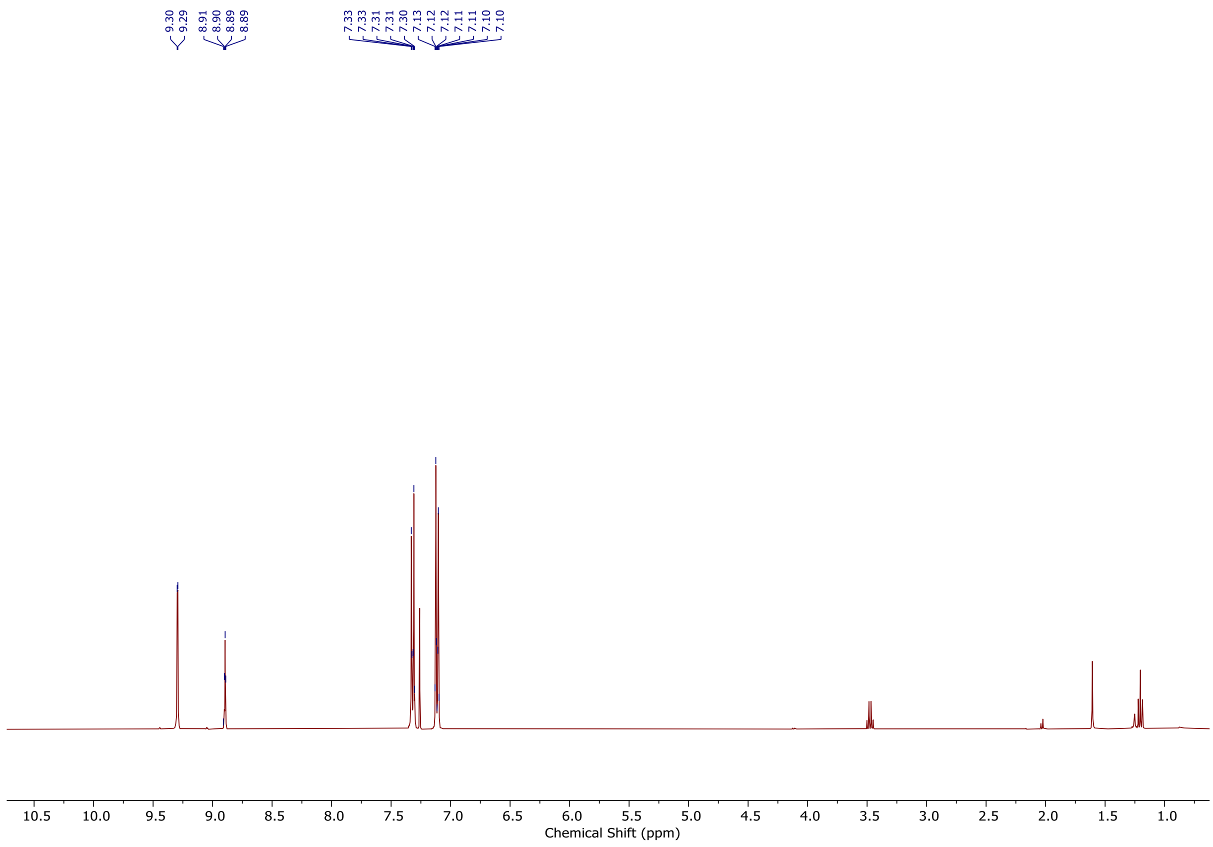

Figure S41. ^{1}H NMR spectrum of **3·ChB^{pCl}** ($CDCl_3$, 400 MHz, 298 K).

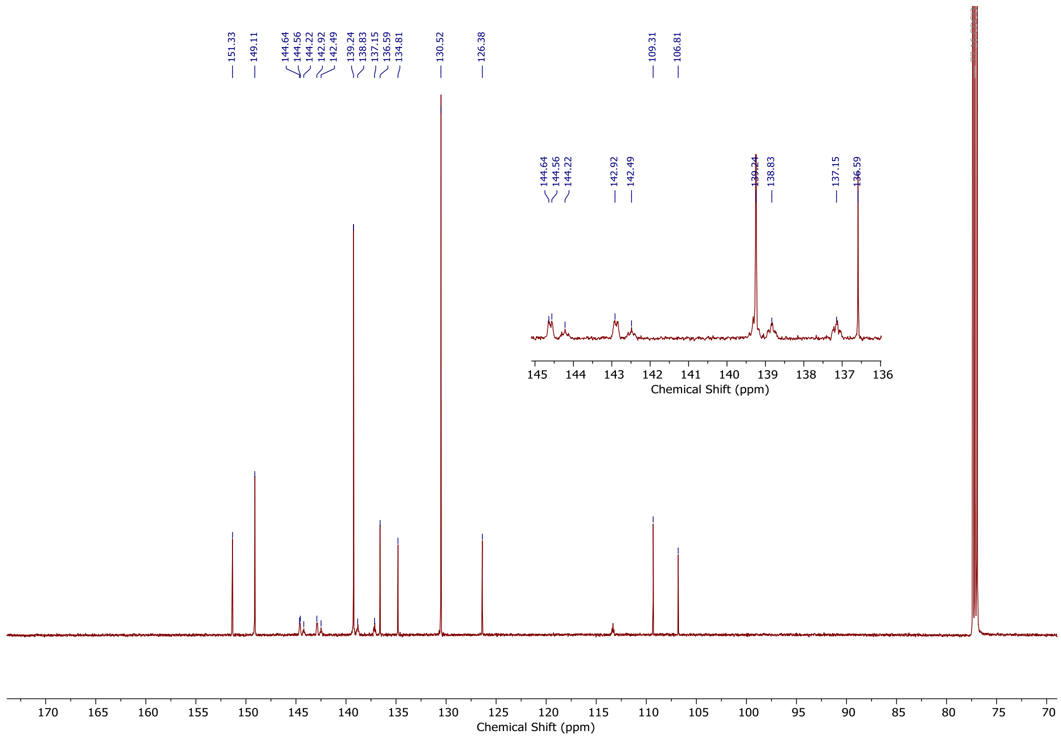

Figure S42. ^{13}C NMR spectrum of **3·ChB^{pCl}** ($CDCl_3$, 151 MHz, 298 K).

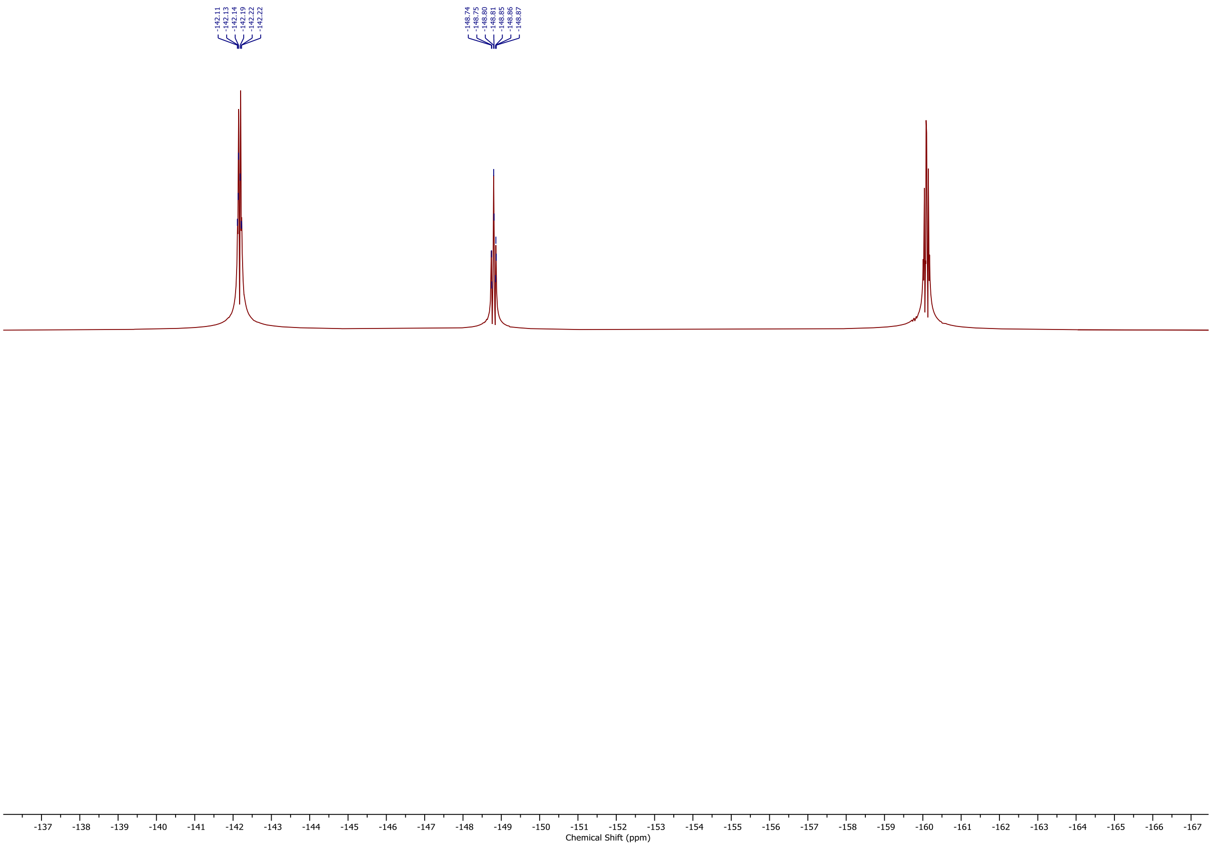

Figure S43. ^{19}F NMR spectrum of **3·ChB^{pCl}** ($CDCl_3$, 377 MHz, 298 K).

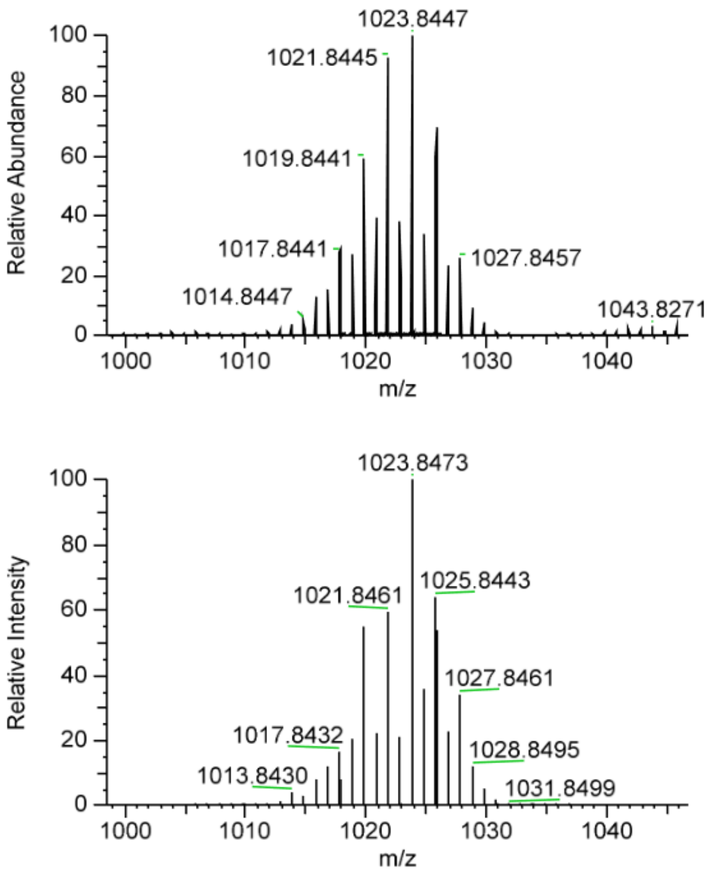

Figure S44. HRESI-MS of **3·ChB^{pCl}**.

3·ChB^{mF}

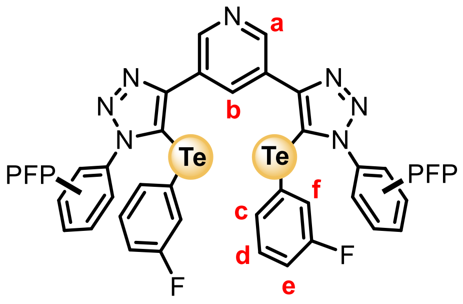

^{1}H NMR (500 MHz, CDCl$_3$) δ 9.30 (d, *J* = 2.1 Hz, 2H$_a$), 8.94 (t, *J* = 2.1 Hz, 1H$_b$), 7.19 – 7.05 (m, 6H$_{c,d,e}$), 6.98 (ddt, *J* = 7.7, 6.6, 1.6 Hz, 2H$_f$).

^{13}C NMR (126 MHz, CDCl$_3$) δ 162.74 (d, *J* = 253.8 Hz), 151.52, 149.19, 143.67 (d, *J* = 223 Hz), 136.84 (d, *J* = 25.5 Hz), 134.81, 133.25 (d, *J* = 3.3 Hz), 131.61 (d, *J* = 7.5 Hz), 126.32, 124.43 (d, *J* = 22.1 Hz), 116.95 (d, *J* = 20.8 Hz), 112.55 (d, *J* = 5.7 Hz), 106.67.

^{19}F NMR (470 MHz, CDCl$_3$) δ -109.55 (dd, *J* = 8.0, 5.3 Hz), -139.23 – -143.79 (m), -147.76 (td, *J* = 21.8, 3.4 Hz), -154.98 – -162.37 (m).

HRMS (ESI +ve) m/z: 989.9042, ([M+H]$^+$, $C_{33}H_{12}N_7F_{12}Te_2$ requires 989.9052.

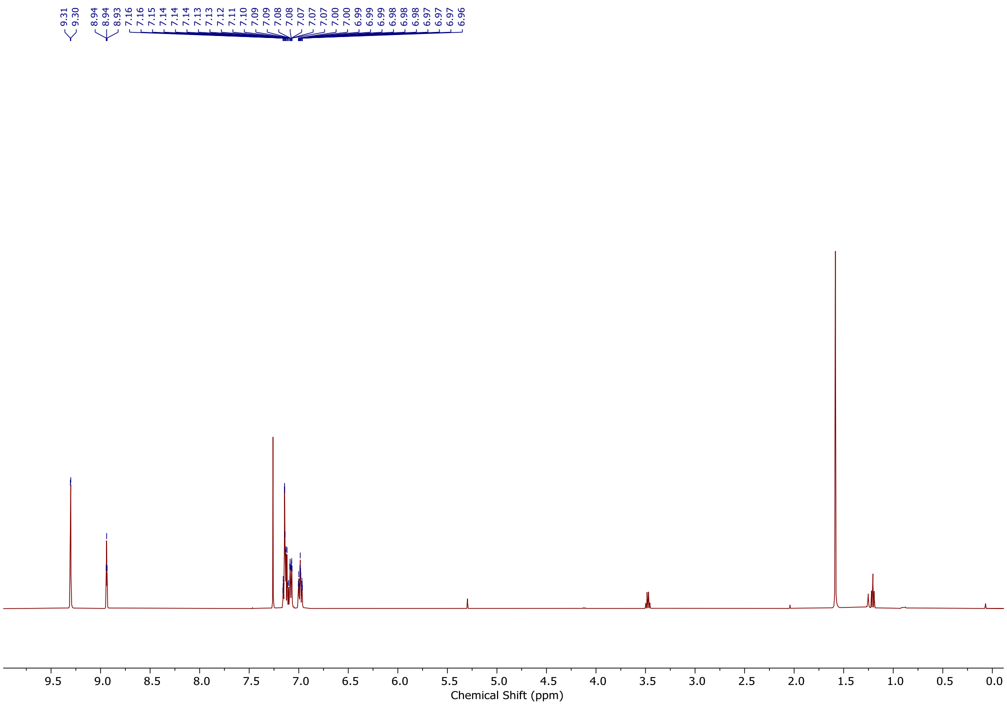

Figure S45. ^{1}H NMR spectrum of **3·ChB^{mF}** ($CDCl_3$, 500 MHz, 298 K).

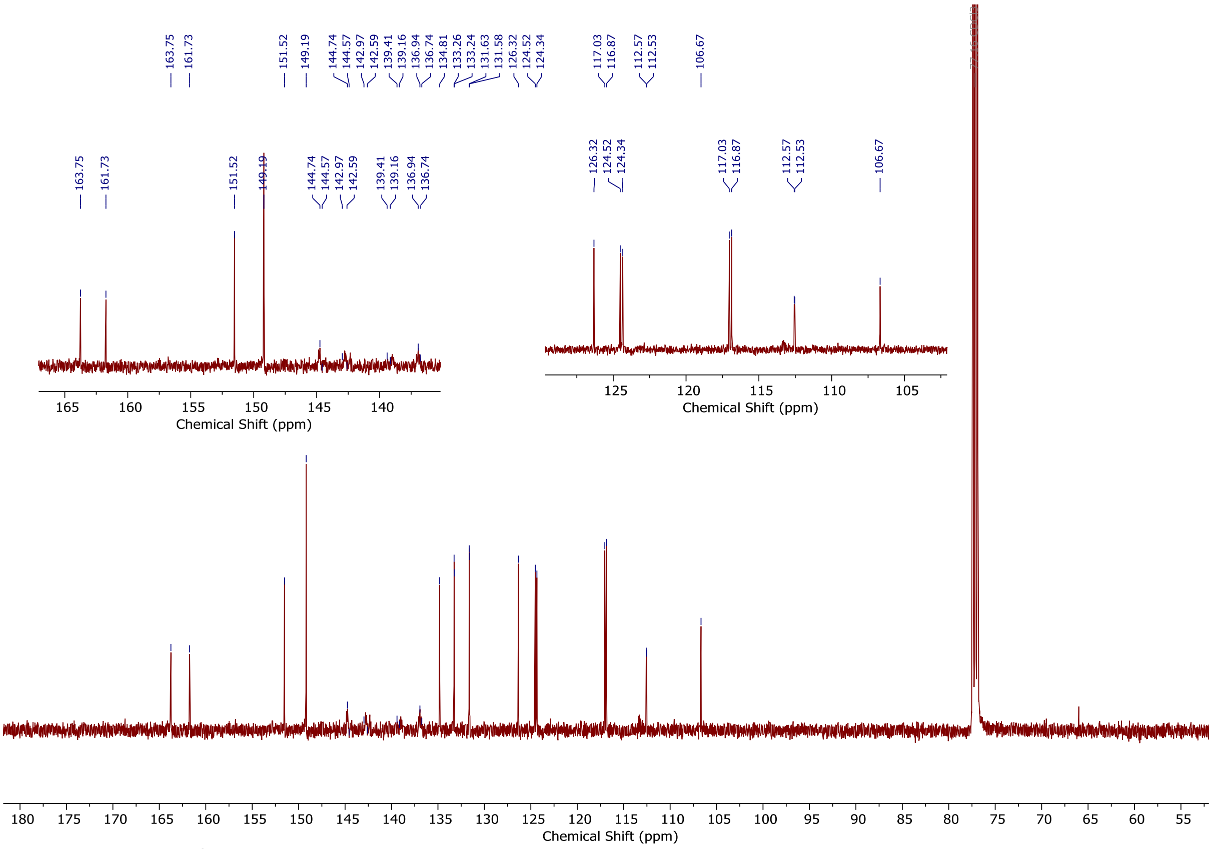

Figure S46. ^{13}C NMR spectrum of **3·ChB^{mF}** ($CDCl_3$, 126 MHz, 298 K).

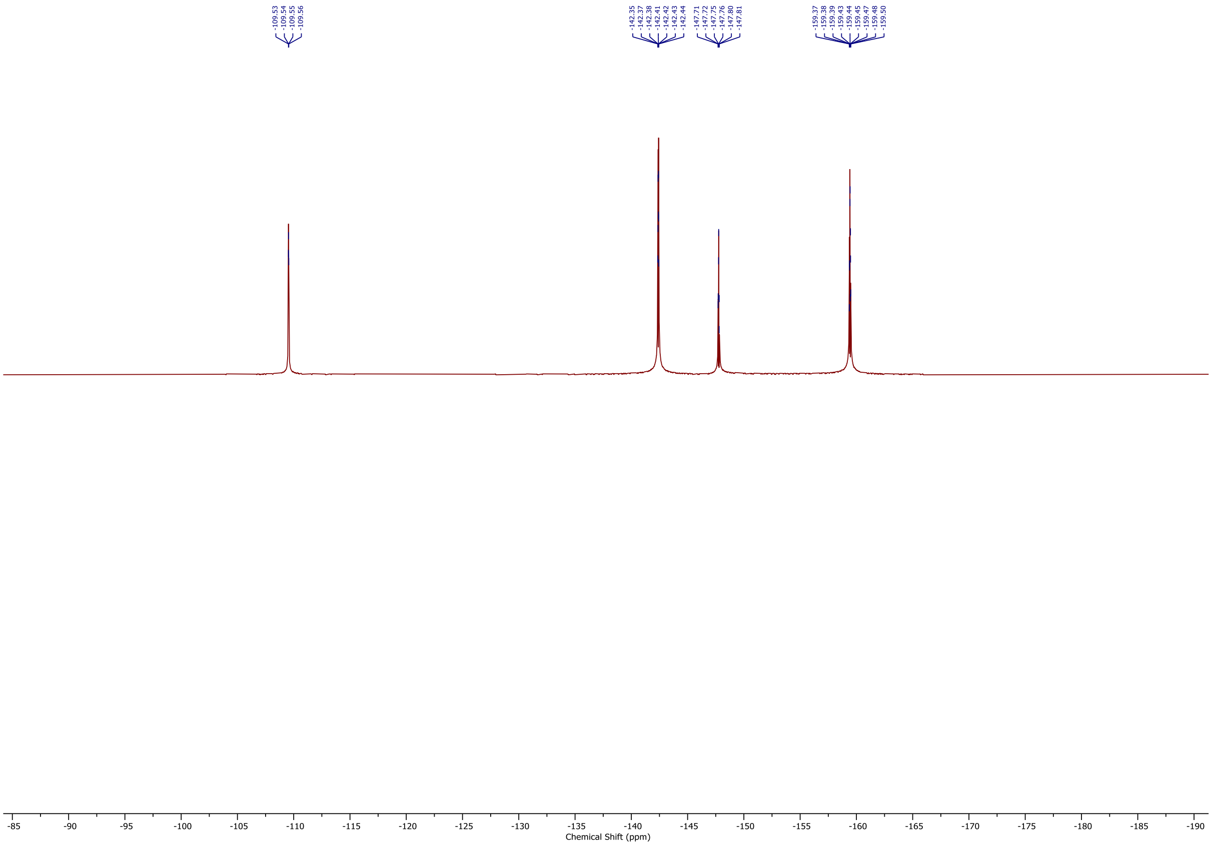

Figure S47. ^{1}H NMR spectrum of **3·ChB^{mF}** ($CDCl_3$, 470 MHz, 298 K).

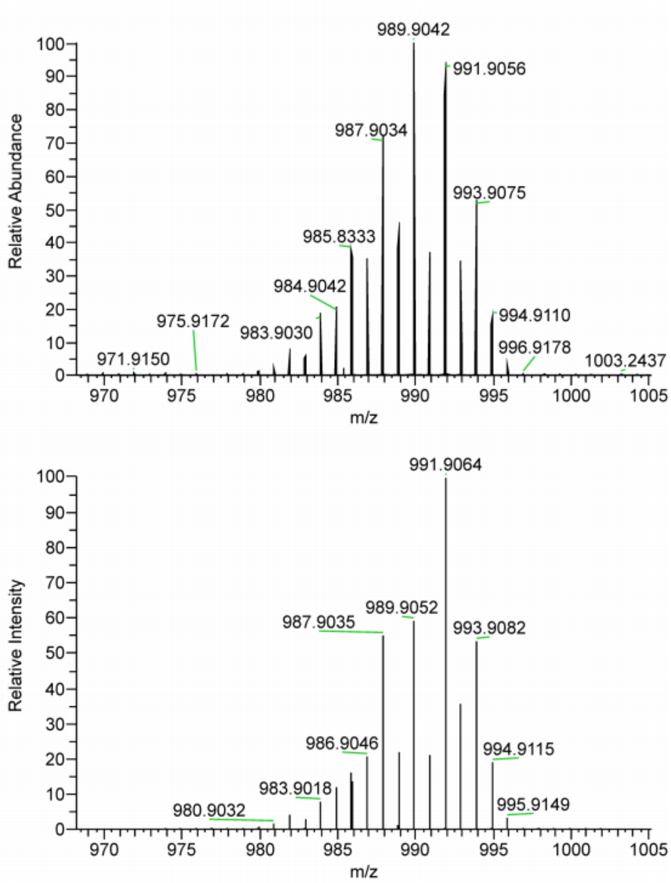

Figure S48. HRESI-MS of **3·ChB^mF^**.

3·ChB^{mCF3}

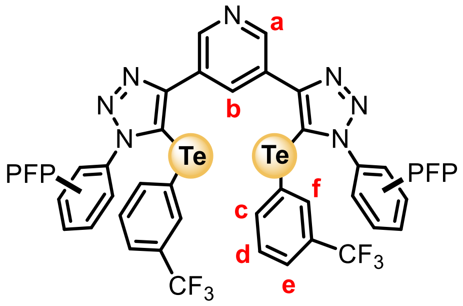

^{1}H NMR (500 MHz, CDCl$_3$) δ 9.32 (d, *J* = 2.1 Hz, 2H$_a$), 8.98 (t, *J* = 2.2 Hz, 1H$_b$), 7.70 –7.52 (m, 6H$_{c,e,f}$), 7.29 (t, *J* = 7.7 Hz, 2H$_d$).

^{13}C NMR (126 MHz, CDCl$_3$) δ 151.43, 149.18, 143.66 (d, *J* = 276 Hz), 141.23, 137.92 (d, *J* = 252 Hz), 134.79, 134.33 (q, *J* = 3.7 Hz), 132.31 (q, *J* = 32.8 Hz), 130.68, 126.53 (q, *J* = 3.6 Hz), 126.32, 124.16, 121.99, 112.29, 106.44.

^{19}F-NMR (470 MHz, CDCl$_3$) δ -63.20, -138.46 – -144.86 (m), -147.36 (d, *J* = 21.4 Hz), -155.74 – -162.59 (m).

HRMS (ESI +ve) m/z: 1089.8979, ([M+H]$^+$, $C_{35}H_{11}N_7F_{16}Te_2$ requires 1089.8989).

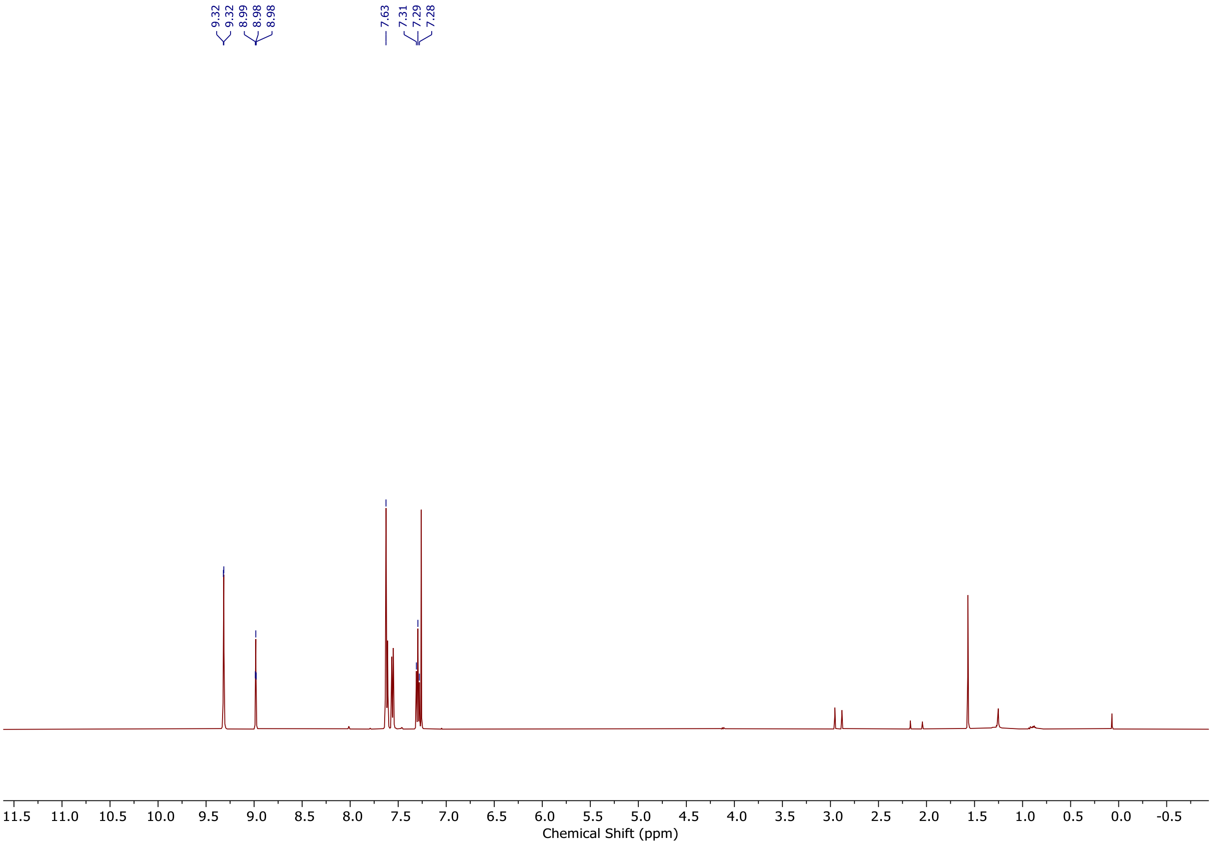

Figure S49. ^{1}H NMR spectrum of **3·ChB^{mCF3}** ($CDCl_3$, 500 MHz, 298 K).

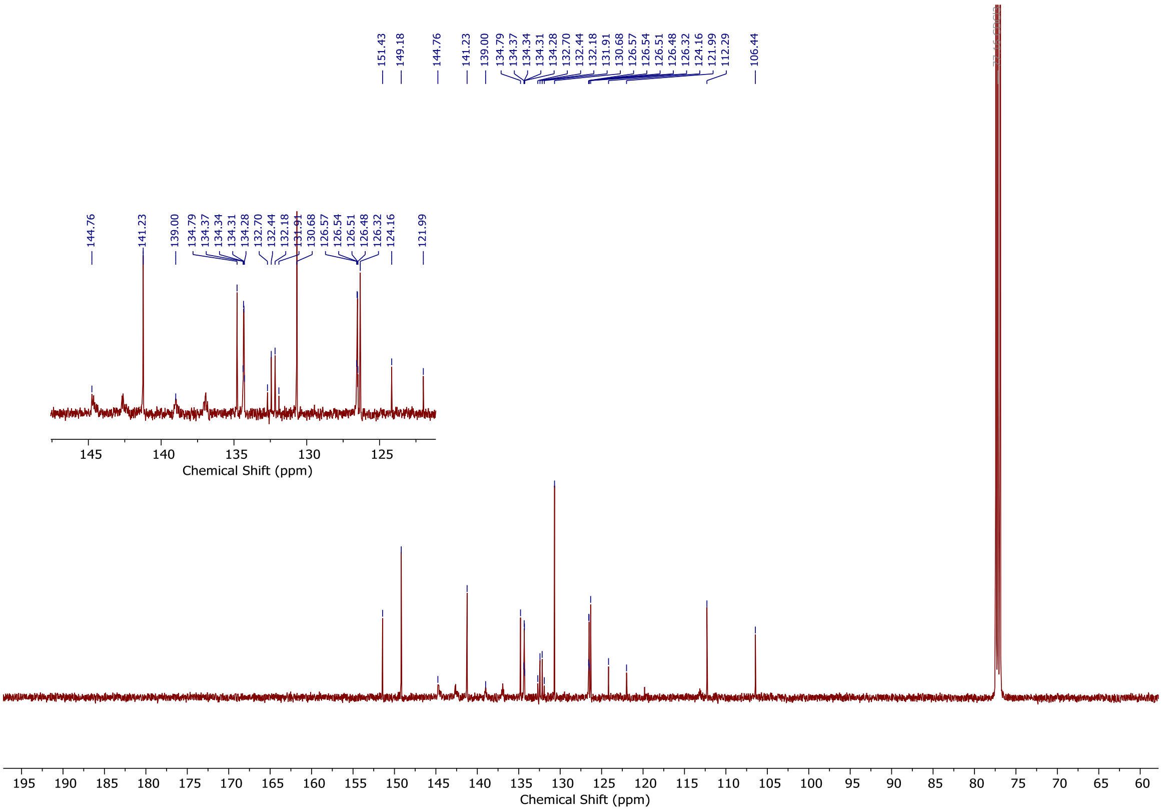

Figure S50. ^{13}C NMR spectrum of **3·ChB^{mCF3}** ($CDCl_3$, 126 MHz, 298 K).

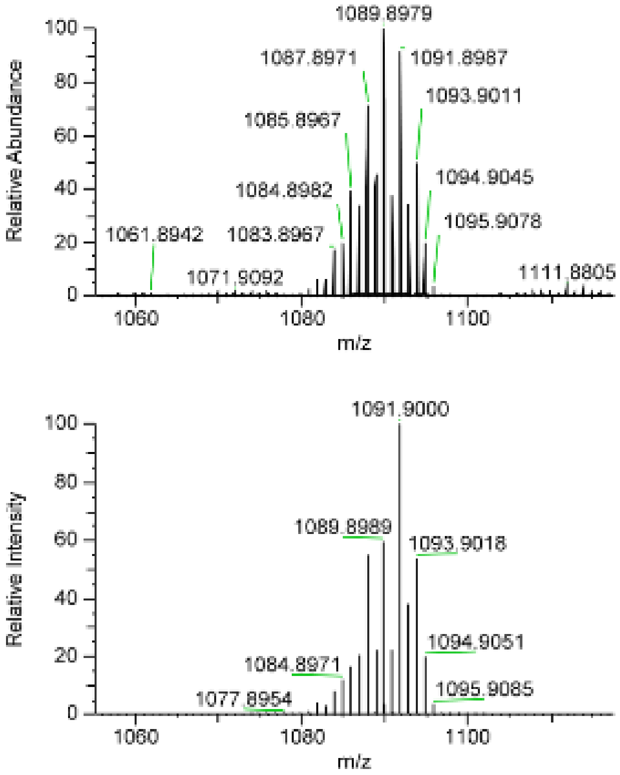

Figure S51. HRESI-MS of **3·ChB^{mCF3}**.

3·ChB^{pCF3}

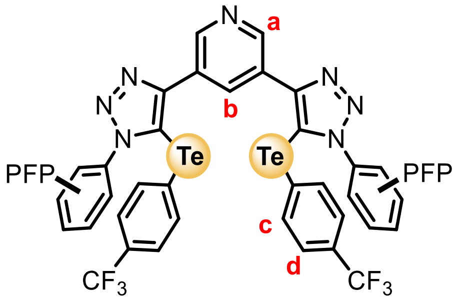

^{1}H NMR (500 MHz, $CDCl_3$) δ 9.30 (d, *J* = 2.1 Hz, $2H_a$), 8.93 (t, *J* = 2.1 Hz, $1H_b$), 7.47 (d, *J* = 8.0 Hz, $2H_c$), 7.38 (d, *J* = 8.0 Hz, $2H_d$).

^{13}C NMR (126 MHz, $CDCl_3$) δ 151.68, 149.13, 144.74 (dm, *J* = 255 Hz), 143.36 (dm, *J* = 260 Hz), 137.99 (dm, *J* = 262 Hz), 137.09 , 134.73, 131.60 (q, *J* = 32.9 Hz), 126.80 (q, *J* = 3.6 Hz), 126.28, 124.64, 122.47, 120.31, 117.12, 113.92 – 112.86 (m), 106.49.

^{19}F NMR (470 MHz, $CDCl_3$) δ -63.28, -142.51 (dd, *J* = 29.4, 10.4 Hz), -145.09 – -151.40 (m), -156.50 – -162.59 (m).

HRMS (ESI +ve) m/z: 1089.8981, ($[M+H]^+$, $C_{35}H_{11}N_7F_{16}Te_2$ requires 1089.8989).

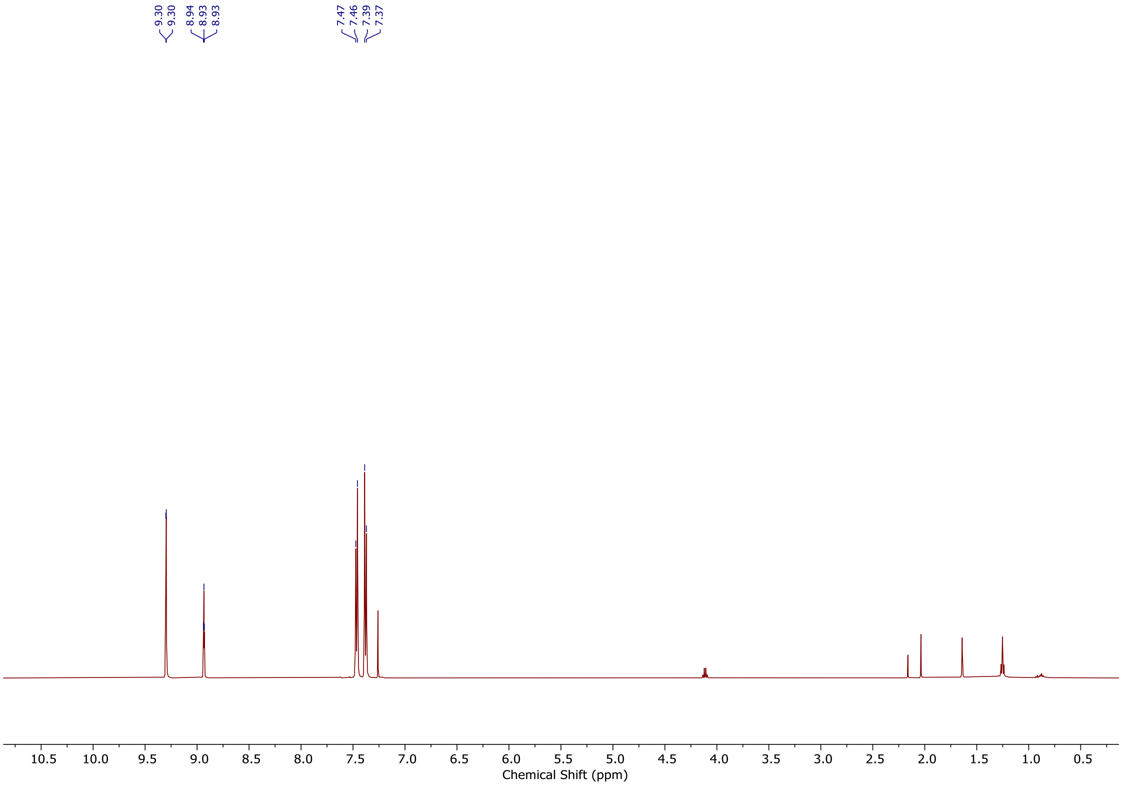

Figure S52. ^{1}H NMR spectrum of **3·ChB^{pCF3}** ($CDCl_3$, 500 MHz, 298 K).

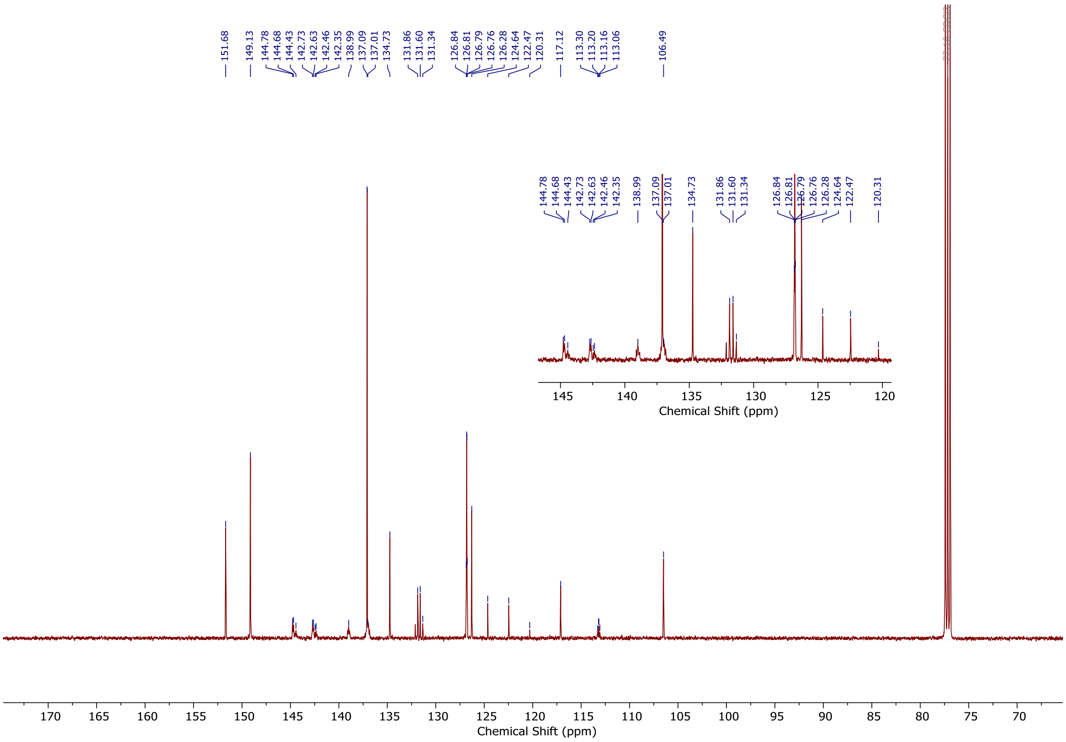

Figure S53. ^{13}C NMR spectrum of **3·ChB^{pCF3}** ($CDCl_3$, 126 MHz, 298 K).

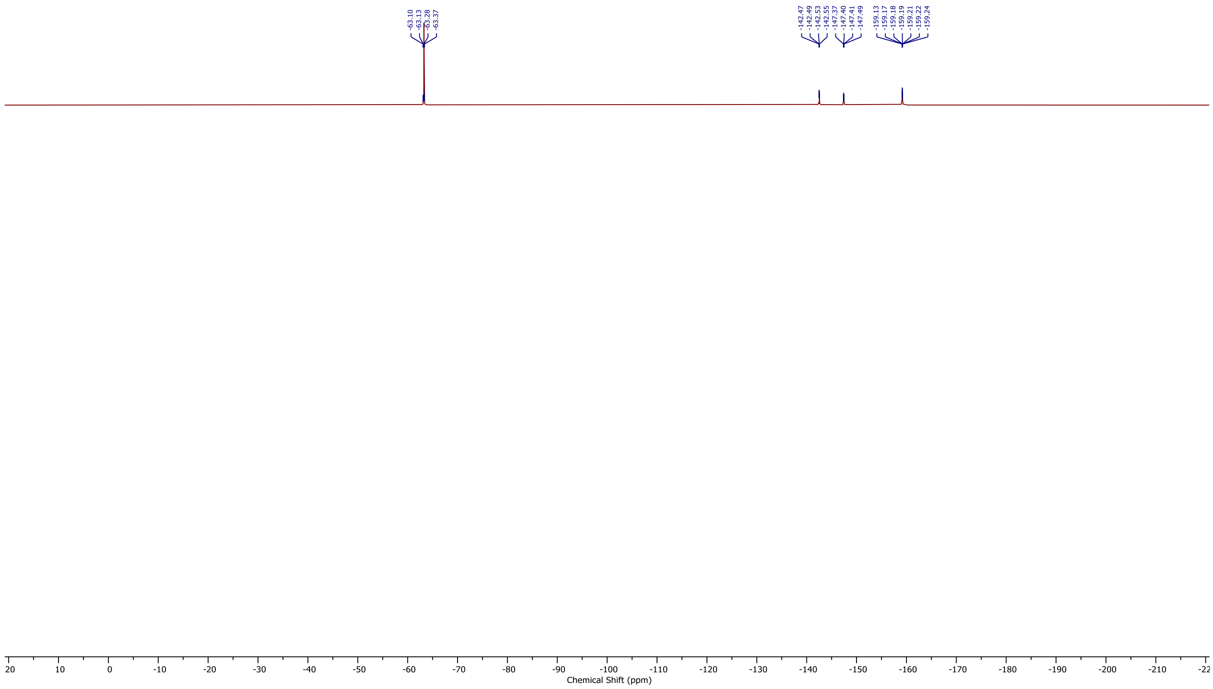

Figure S54. ^{19}F NMR spectrum of **3·ChB^{pCF3}** ($CDCl_3$, 470 MHz, 298 K).

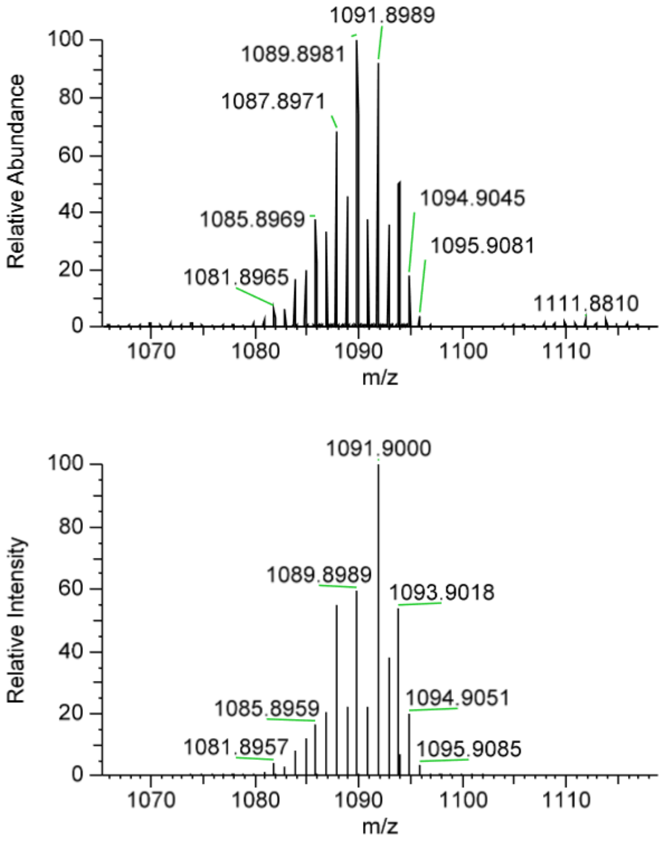

Figure S55. HRESI-MS of **3·ChB**$^{\mathbf{pCF3}}$.

3·ChB^Ph^

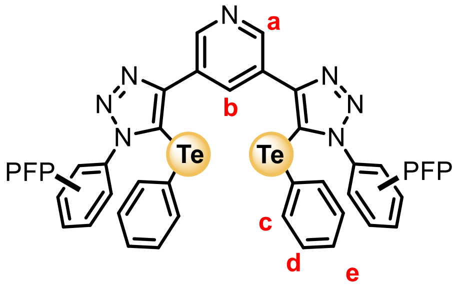

^{1}H NMR (500 MHz, $CDCl_3$) δ = 9.31 (d, *J* = 2.1 Hz, $2H_a$), 9.03 (t, *J* = 2.1 Hz, $1H_b$), 7.41 (dd, *J* = 8.1, 1.3 Hz, $4H_c$), 7.34 – 7.27 (m, $2H_e$), 7.18 – 7.10 (m, $4H_d$).

^{13}C NMR (126 MHz, $CDCl_3$) δ = 150.61, 147.69, 143.71 (dm, *J* = 258 Hz), 143.28 (dm, *J* = 261 Hz), 138.25, 137.92 (dm, *J* = 255 Hz), 135.65, 130.26, 129.80, 127.07, 113.34 (td, *J* = 16.4 Hz, *J* = 3.8 Hz, 2C), 111.55, 107.25.

^{19}F NMR (377 MHz, $CDCl_3$) δ = -142.12 – -142.29 (m), -148.38 (dd, *J* = 21.8, 3.2 Hz), -159.56 – -159.85 (m).

HRMS (ESI +ve) m/z: 953.9233, ($[M+H]^+$, $C_{33}H_{14}N_7F_{10}Te_2$ requires 953.9241).

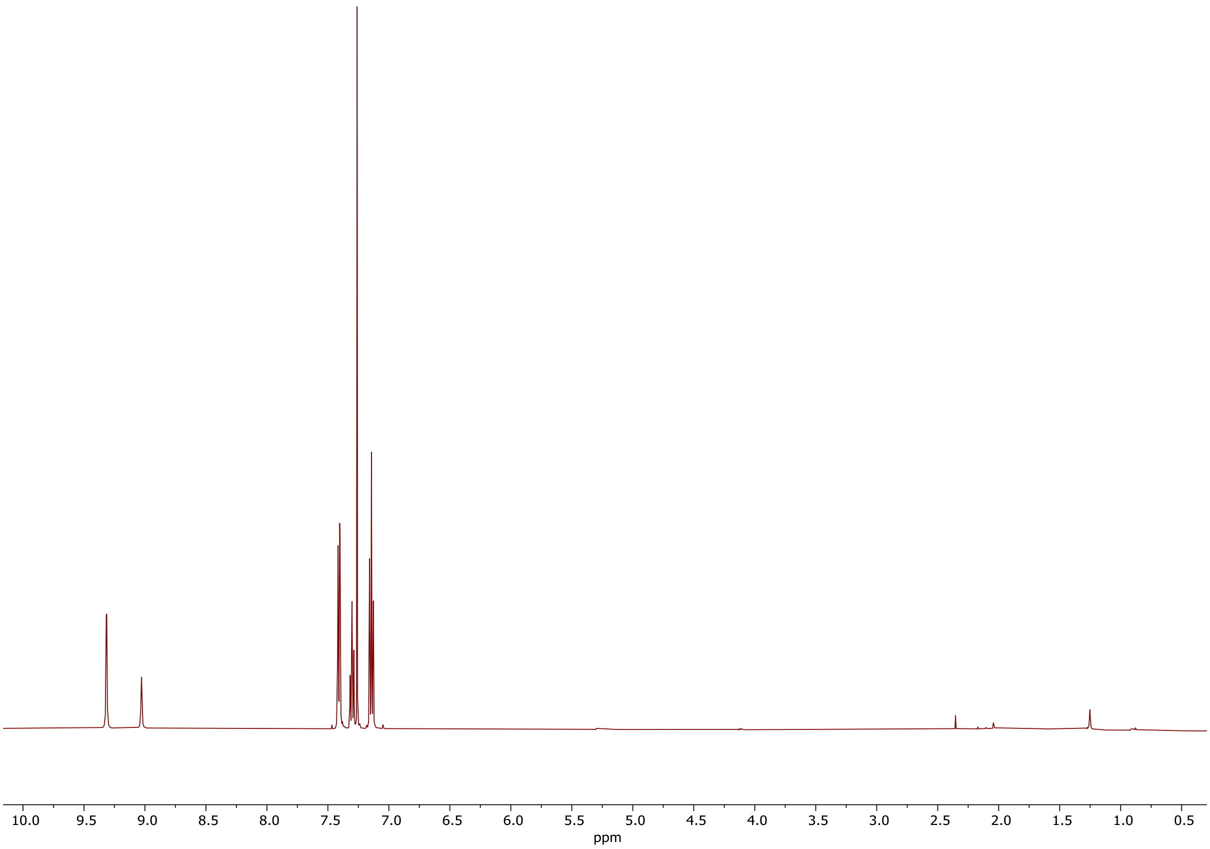

Figure S56. ^{1}H NMR spectrum of **3·ChB^{Ph}** ($CDCl_3$, 500 MHz, 298 K).

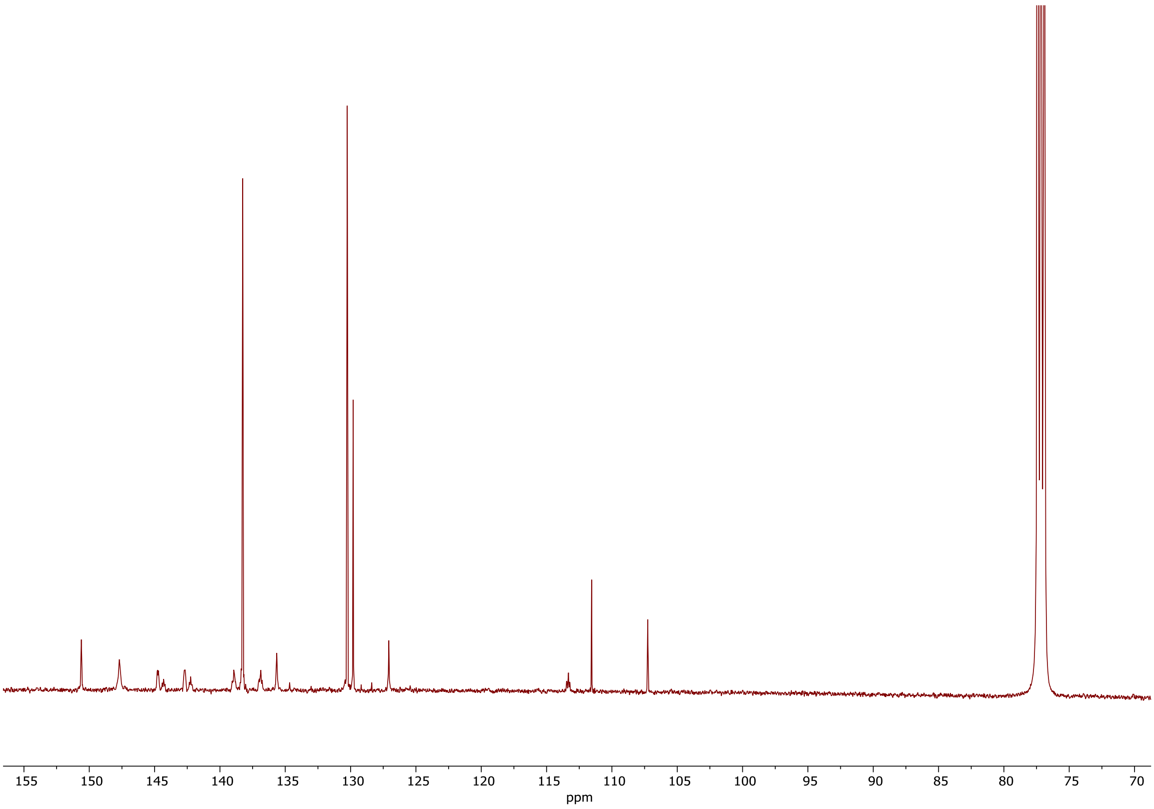

Figure S57. ^{13}C NMR spectrum of **3·ChB^{Ph}** ($CDCl_3$, 126 MHz, 298 K).

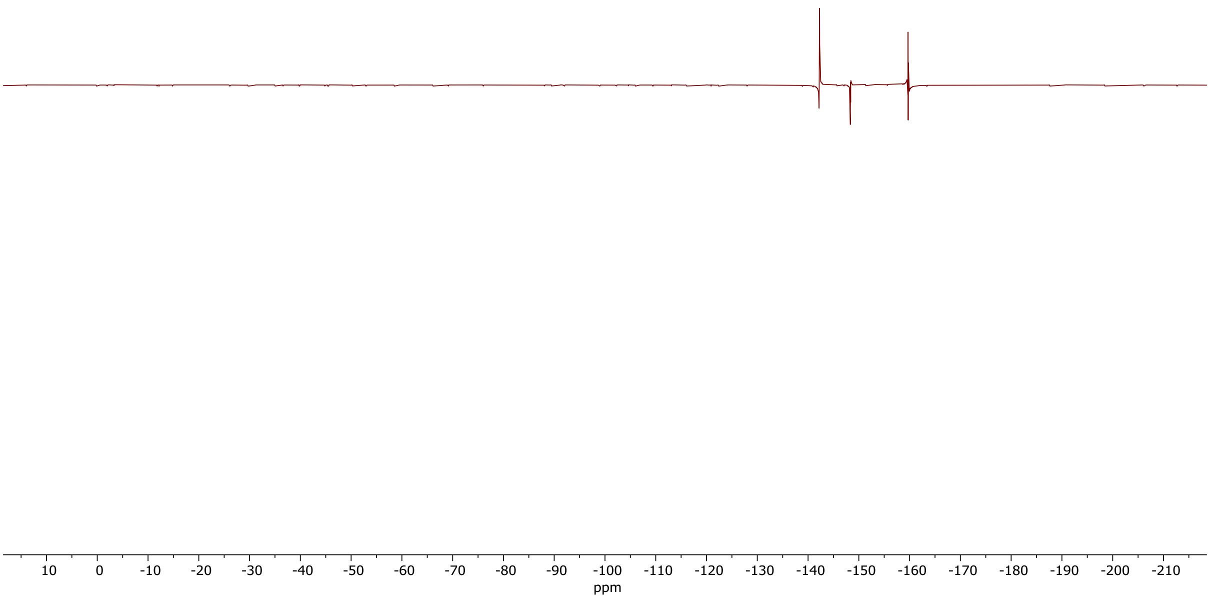

Figure S58. ^{19}F NMR spectrum of **3·ChB^{Ph}** ($CDCl_3$, 377 MHz, 298 K).

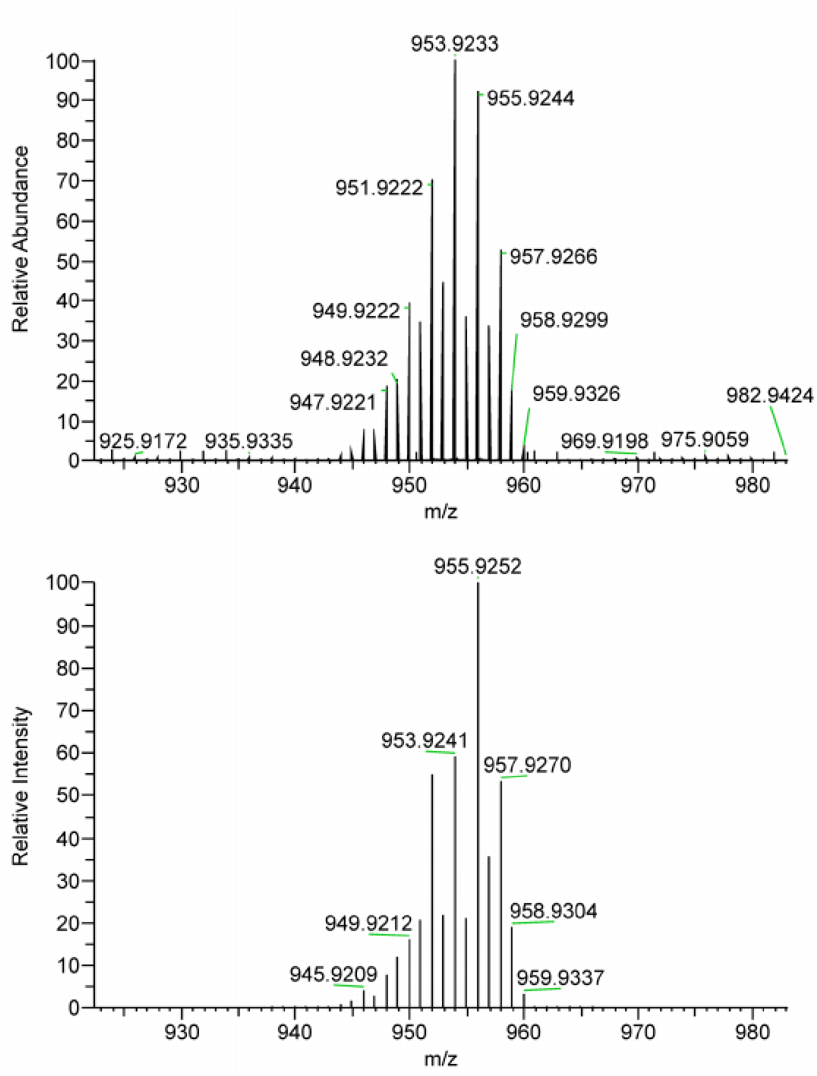

Figure S59. HRESI-MS of **3·ChB^{Ph}**.

3·ChB^{pF}

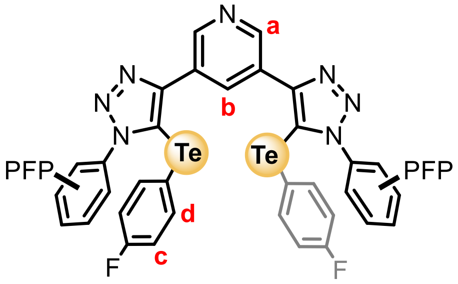

^{1}H NMR (400 MHz, $CDCl_3$) δ = 9.32 (d, *J* = 2.2 Hz, 2H_a), 8.96 (s, 1H_b), 7.42 (dd, *J* = 8.7, 5.4 Hz, 4H_c), 6.86 (t, *J* = 8.6 Hz, 4H_d).

^{13}C NMR (126 MHz, $CDCl_3$) δ = 163.87 (d, *J* = 252 Hz,), 150.79, 148.16, 143.76 (dm, *J*= 259 Hz), 143.36 (dm, *J* = 260 Hz), 140.78 (d, *J* = 8.9 Hz,), 137.99 (dm, *J* = 261 Hz), 135.45, 126.82, 117.83 (d, *J* = 21.4 Hz), 113.33 (td, *J* = 14.5 Hz, *J* = 4.0 Hz,), 107.18, 105.48 (d, *J* = 3.8 Hz).

^{19}F NMR (376 MHz, $CDCl_3$) δ = -109.17, -142.46 (dt, *J* = 21.7, 4.9 Hz), -147.75 (t, *J* = 21.6 Hz), -159.40 (dd, *J* = 21.5, 17.1 Hz).

HRMS (ESI +ve) m/z: 989.9042, $([M+H]^+)$, $C_{33}H_{12}N_7F_{12}Te_2$ requires 989.9052.

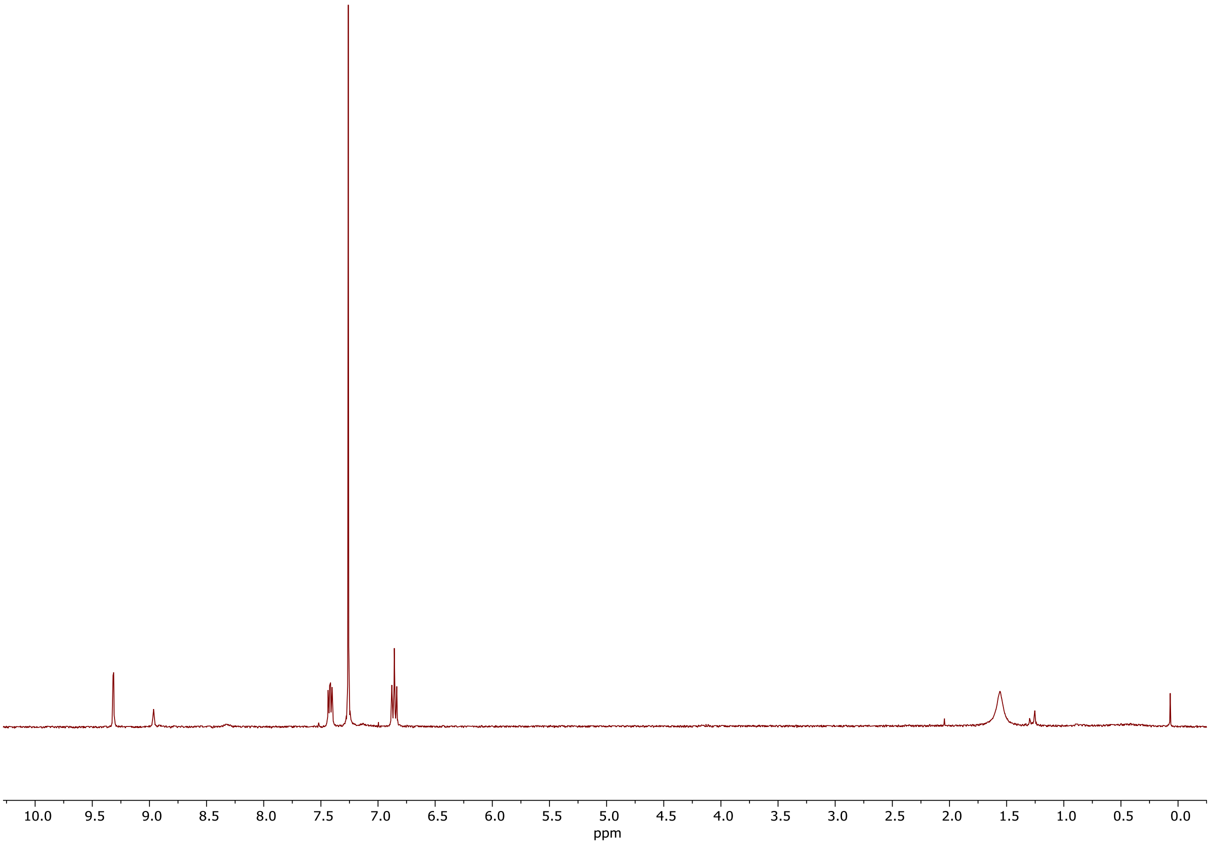

Figure S60. ^{1}H NMR spectrum of **3·ChB^{pF}** ($CDCl_3$, 400 MHz, 298 K).

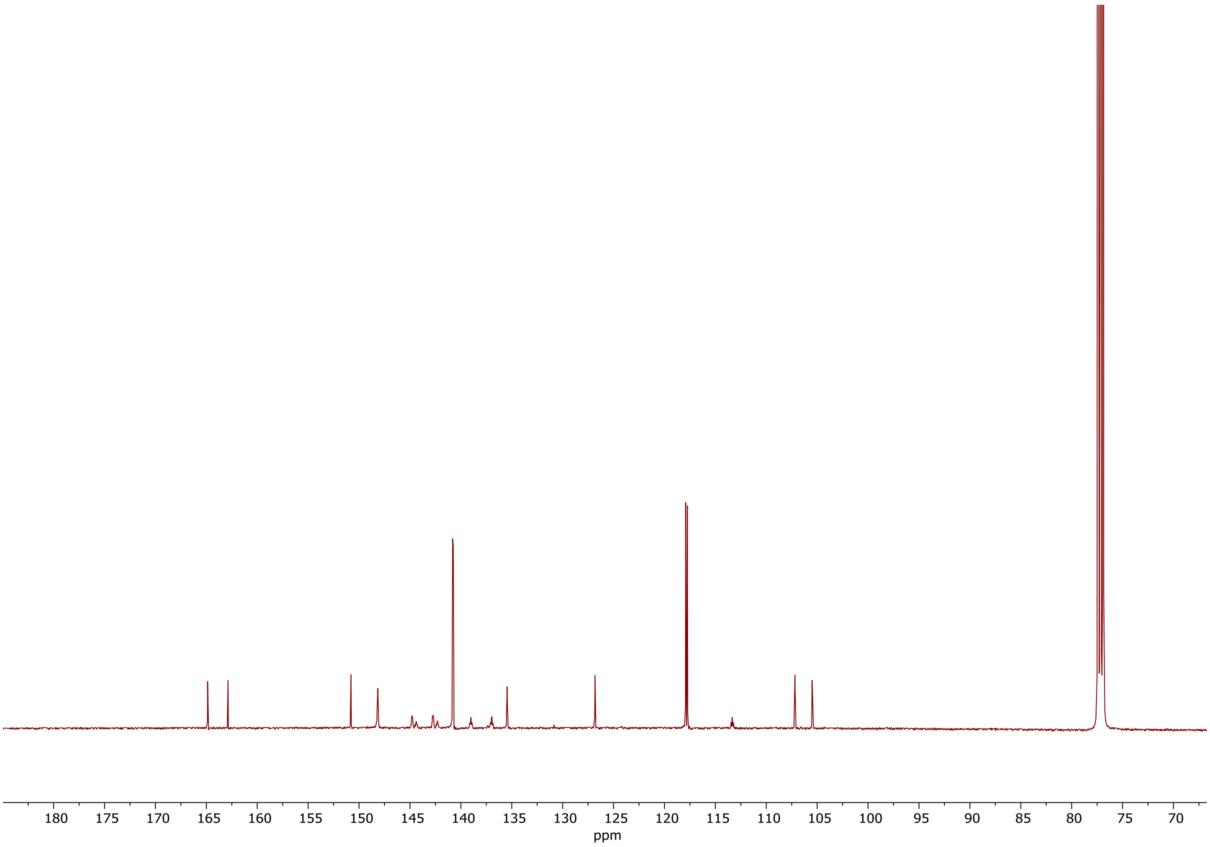

Figure S61. ^{13}C NMR spectrum of **3·ChB^{pF}** ($CDCl_3$, 126 MHz, 298 K).

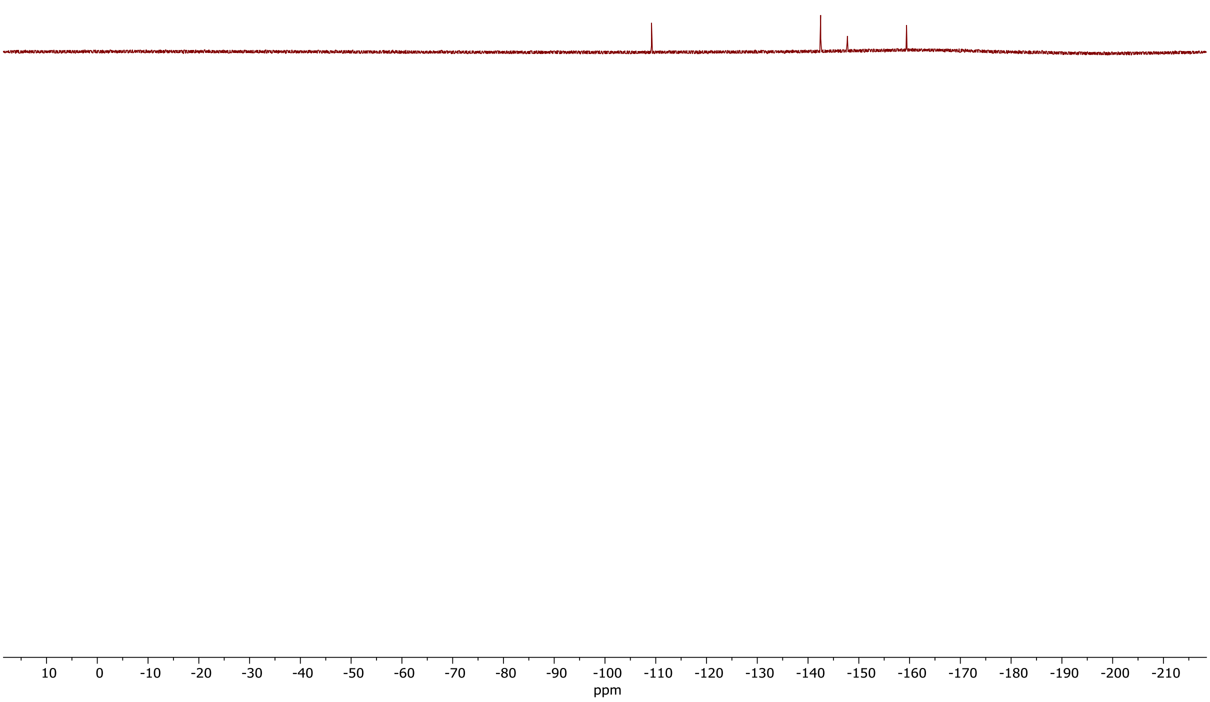

Figure S62. ^{19}F NMR spectrum of **3·ChB^{pF}** ($CDCl_3$, 376 MHz, 298 K).

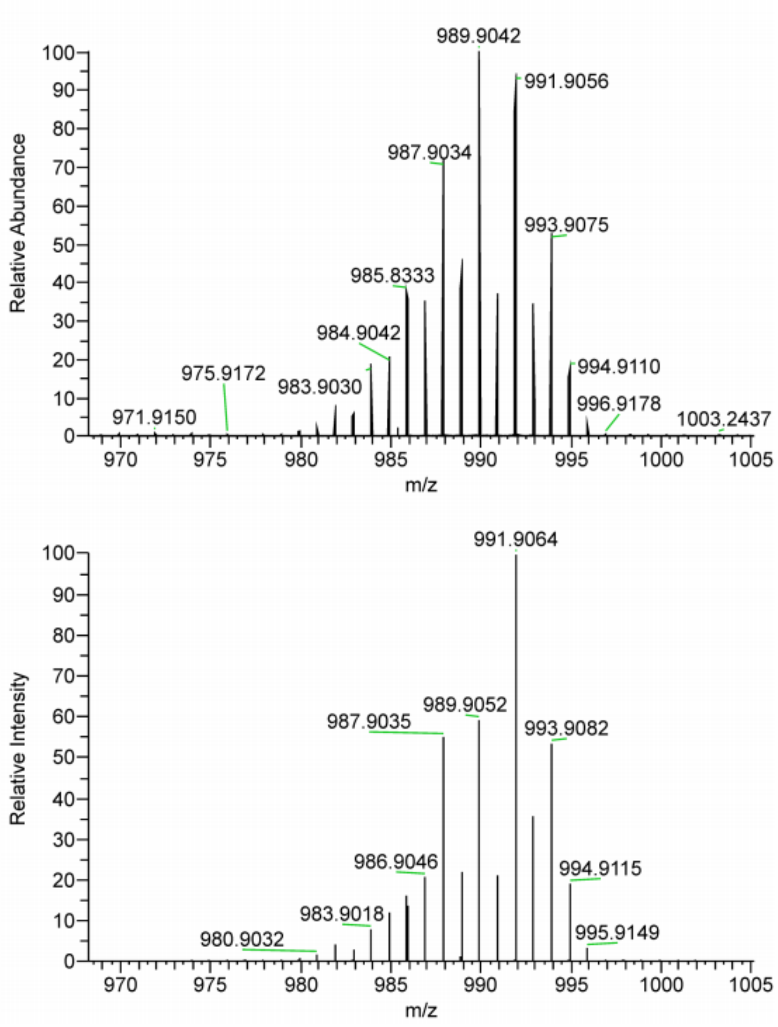

Figure S63. HRESI-MS of **3·ChB^{pF}**.

3·ChB^{2F}

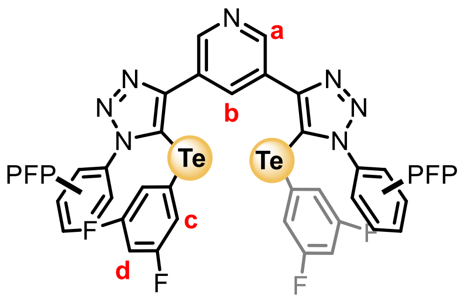

^{1}H NMR (400 MHz, CDCl$_3$) δ = 9.36 (d, J = 2.1 Hz, 2H$_a$), 9.12 (s, 1H$_b$), 6.88 (ddd, J = 5.0, 2.3, 1.1 Hz, 4H$_c$), 6.73 (tt, J = 8.8, 2.3 Hz, 2H$_d$).

^{13}C NMR (126 MHz, CDCl$_3$) δ = 163.21 (dd, J = 257 Hz, J = 11.3 Hz,), 151.38, 148.22, 143.61 (dm, J = 257 Hz), 143.54 (dm, J = 263 Hz), 138.05 (dm, J = 257 Hz), 135.50, 126.67, 119.79 (dd, J = 20.2 Hz, J = 6.3 Hz), 113.24 (t, J = 7.56 Hz,), 113.08 – 112.93 (m,), 106.70, 105.64 (t, J = 25.2 Hz)

HRMS (ESI +ve) m/z: 1025.8853, ([M+H]$^+$, $C_{33}H_{10}N_7F_{14}Te_2$ requires 1025.8864.

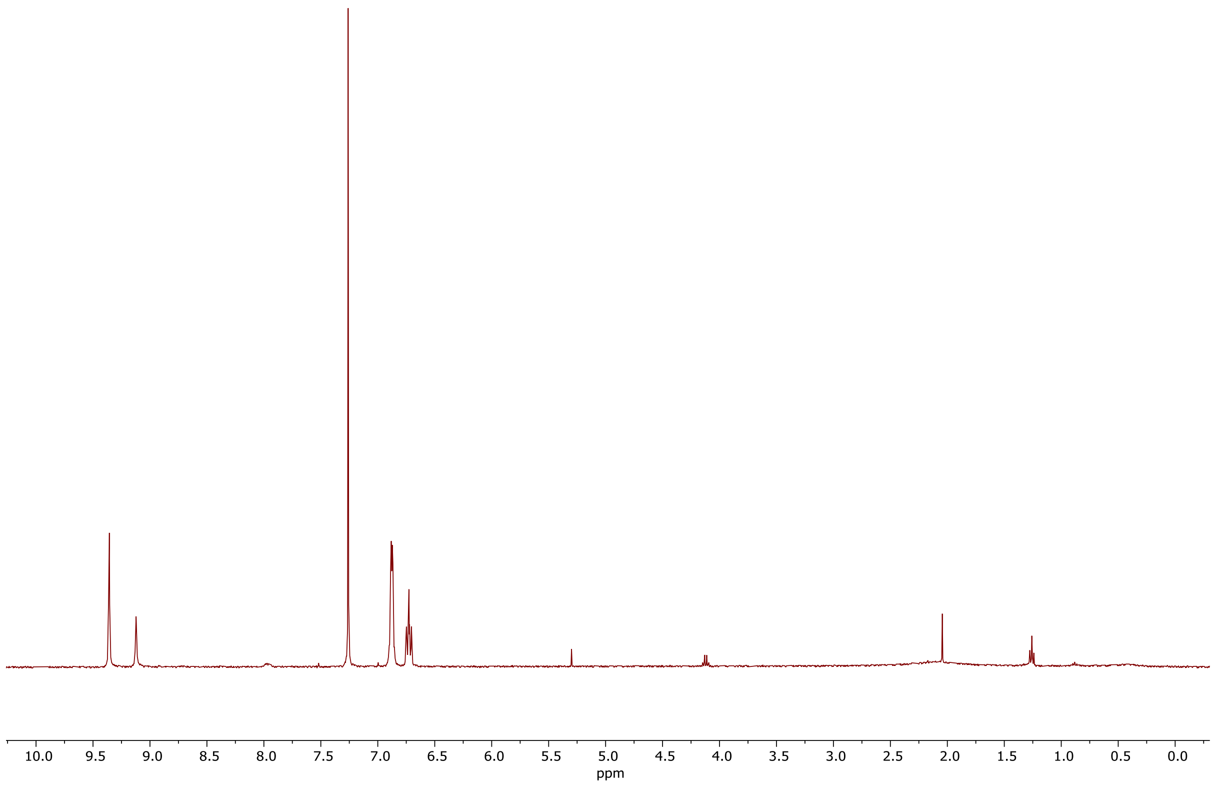

Figure S64. ^{1}H NMR spectrum of **3·ChB^{2F}** ($CDCl_3$, 400 MHz, 298 K).

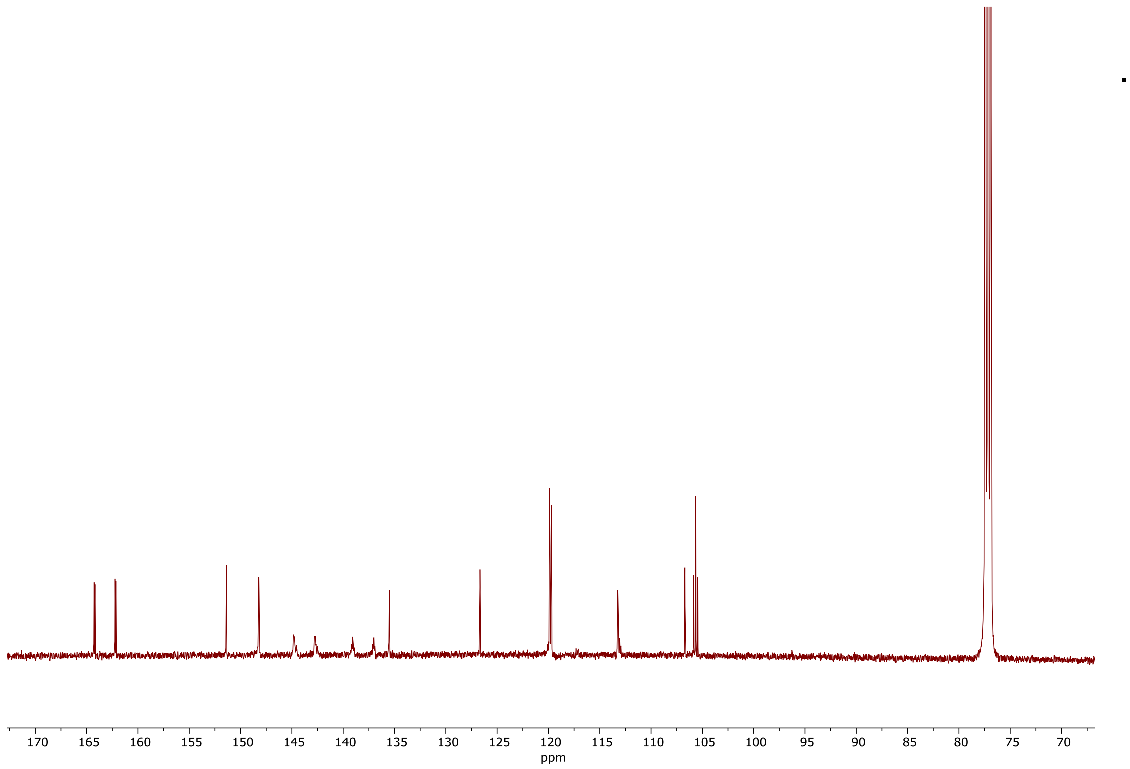

Figure S65. ^{13}C NMR spectrum of **3·ChB^{2F}** ($CDCl_3$, 126 MHz, 298 K).

.

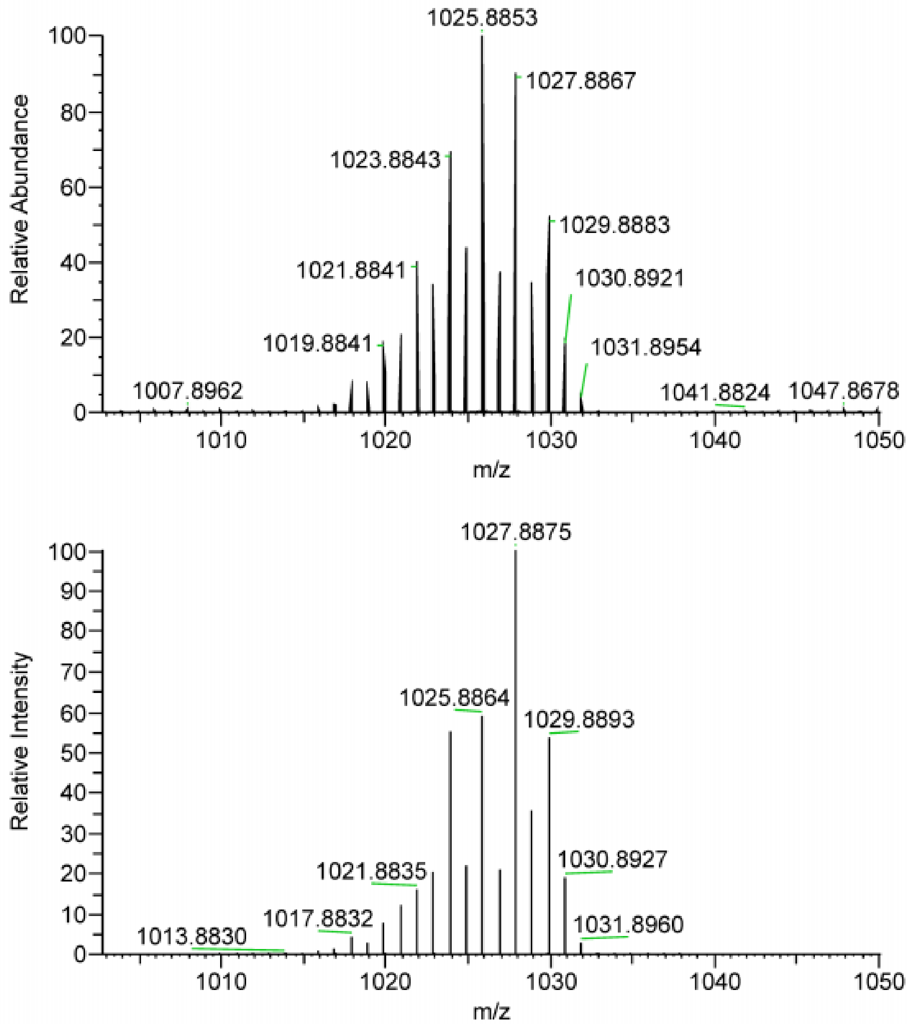

Figure S66. HRMS of **3·ChB^{pF}**.

3·ChB^{3F}

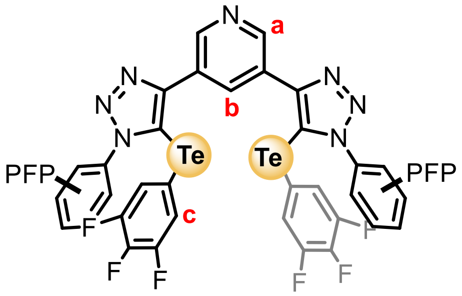

^{1}H NMR (400 MHz, $CDCl_3$) δ = 9.37 (d, *J* = 2.1 Hz, 2H_a), 9.10 (t, *J* = 2.1 Hz, 1H_b), 7.07 – 6.99 (m, 4H_c).

^{13}C NMR (126 MHz, $CDCl_3$) δ = 151.73 (ddd, *J* = 259 Hz, *J* = 10.1 Hz, *J* = 3.78 Hz), 151.34, 148.42, 143.73 (dm, *J* = 258 Hz), 143.60 (dm, *J* = 262 Hz), 141.07 (dt, *J* = 258 Hz, *J* = 15.1 Hz), 138.11 (dm, *J* = 258 Hz), 135.41, 126.54, 121.80 (dd, *J* = 16.4 Hz, *J* = 5.04 Hz), 113.05 (td, *J* = 14.2 Hz, *J* = 3.4 Hz), 106.69, 104.26 – 104.14 (m, 2C).

HRMS (ESI +ve) m/z: 1061.8662, ($[M+H]^+$, $C_{33}H_8N_7F_{18}Te_2$ requires 1061.8676.

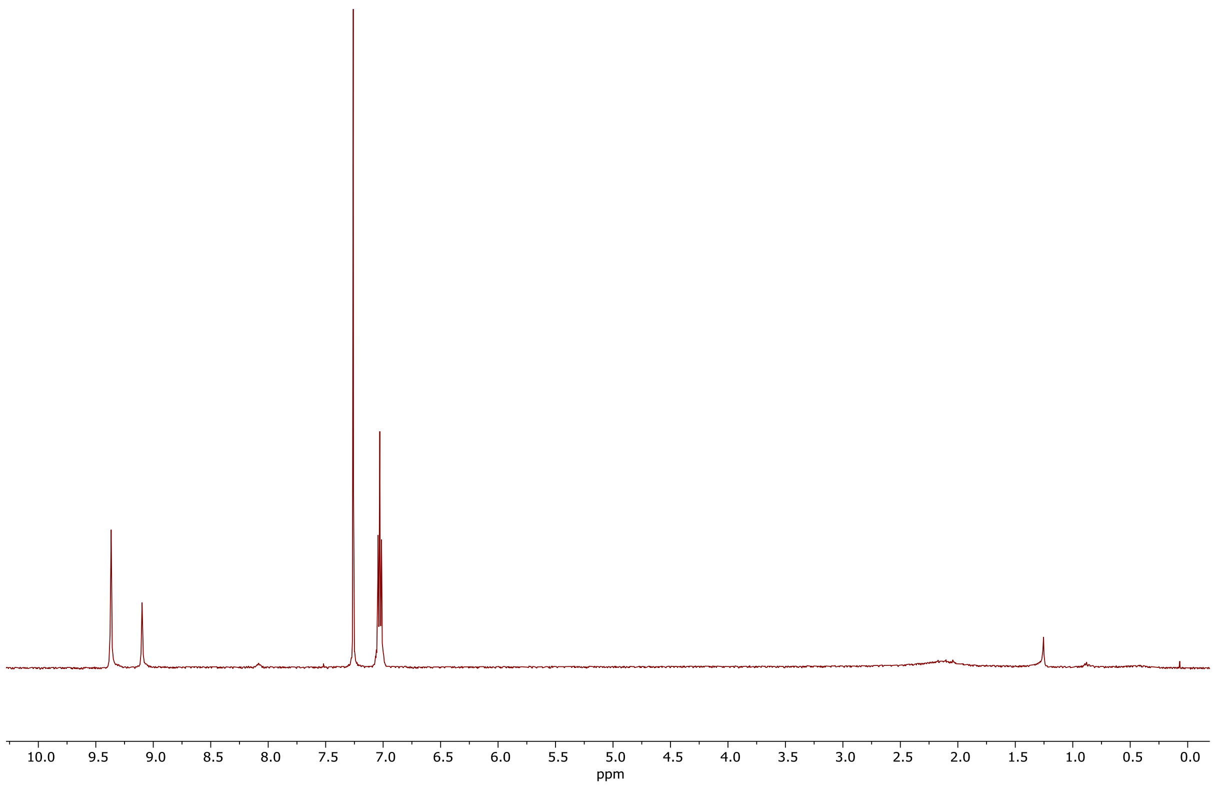

Figure S67. ^{1}H NMR spectrum of **3·ChB^{3F}** **(**CDCl$_3$, 400 MHz, 298 K).

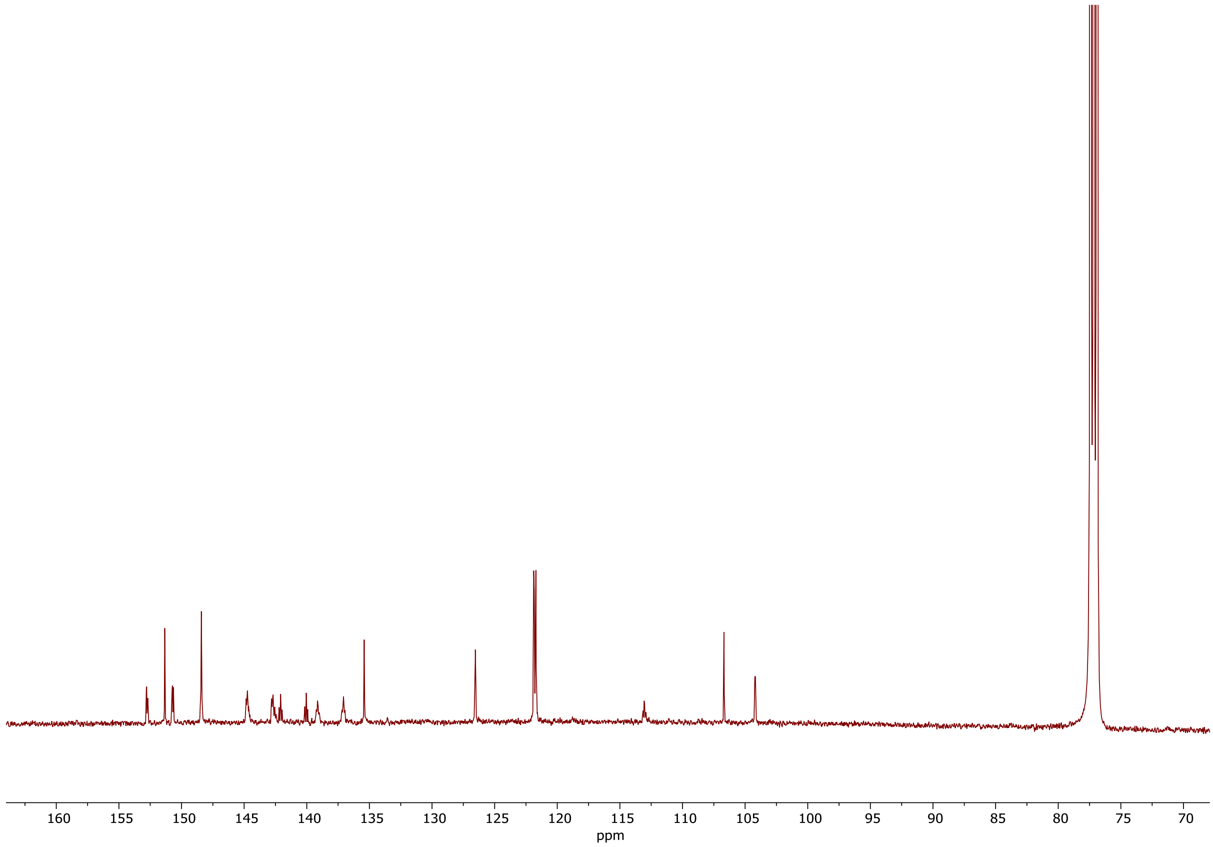

Figure S68. ^{13}C NMR spectrum of **3·ChB^{3F}** (CDCl$_3$, 126 MHz, 298 K).

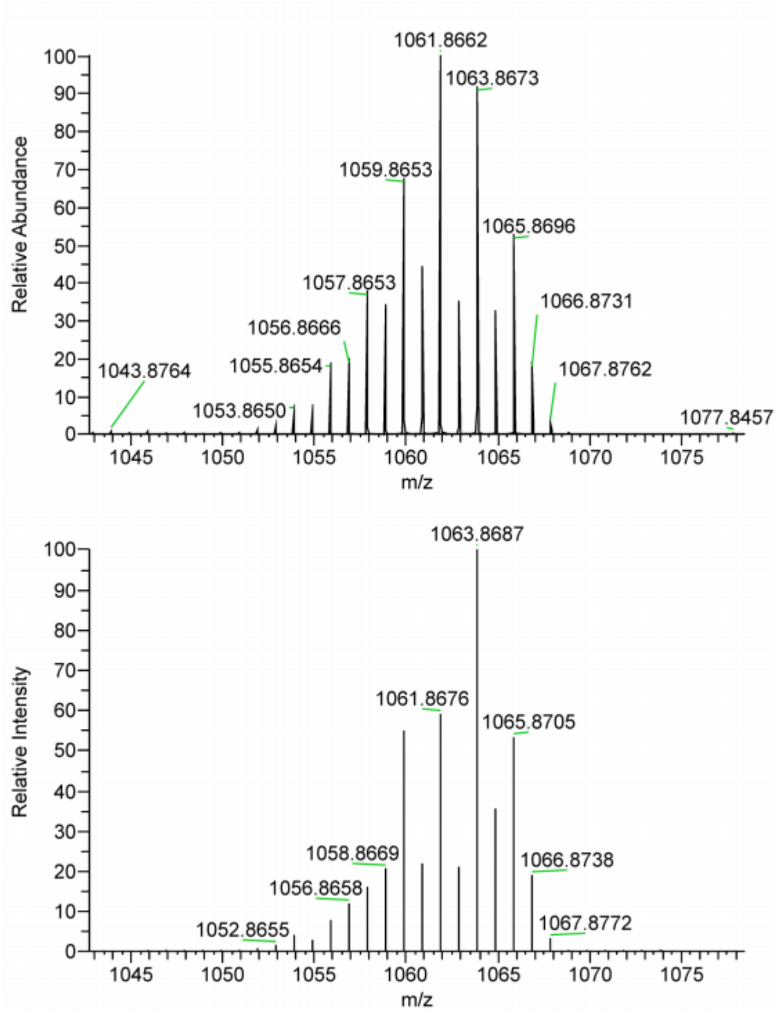

Figure S69. HRESI-MS of **3·ChB^{3F}**.

3·ChB^{2CF3}

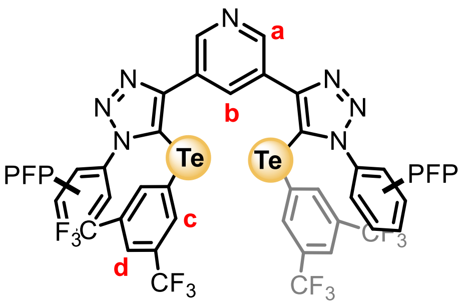

^{1}H NMR (500 MHz, $CDCl_3$) δ = 9.38 (d, J = 2.1 Hz, 2H_a), 9.17 (t, J = 2.1 Hz, 1H_b), 7.83 (d, J = 1.6 Hz, 4H_c), 7.79 (s, 2H_d).

^{13}C NMR (126 MHz, $CDCl_3$) δ = 151.04, 147.97, 143.64 (dm, J= 262 Hz), 138.07 (dm, J = 255 Hz), 137.31 (d, J = 3.8 Hz), 135.61, 133.24 (q, J = 34.0 Hz), 126.74, 123.68 – 123.59 (m), 122.31 (q, J = 273 Hz,), 113.62, 113.01 – 112.72 (m), 106.35. (Missing signal due to coalescence).

^{19}F NMR (376 MHz, $CDCl_3$) δ = -63.39, -142.76 – -142.92 (m), -145.42 (dt, J = 19.1, 3.6 Hz), -146.77 (tt, J = 21.2, 3.4 Hz), -149.20 (tt, J = 21.2, 2.7 Hz), -158.76 – -159.10 (m).

HRMS (ESI +ve) m/z: 1225.8724, $([M+H]^+$, $C_{37}H_{10}N_7F_{22}Te_2$ requires 1225.8736.

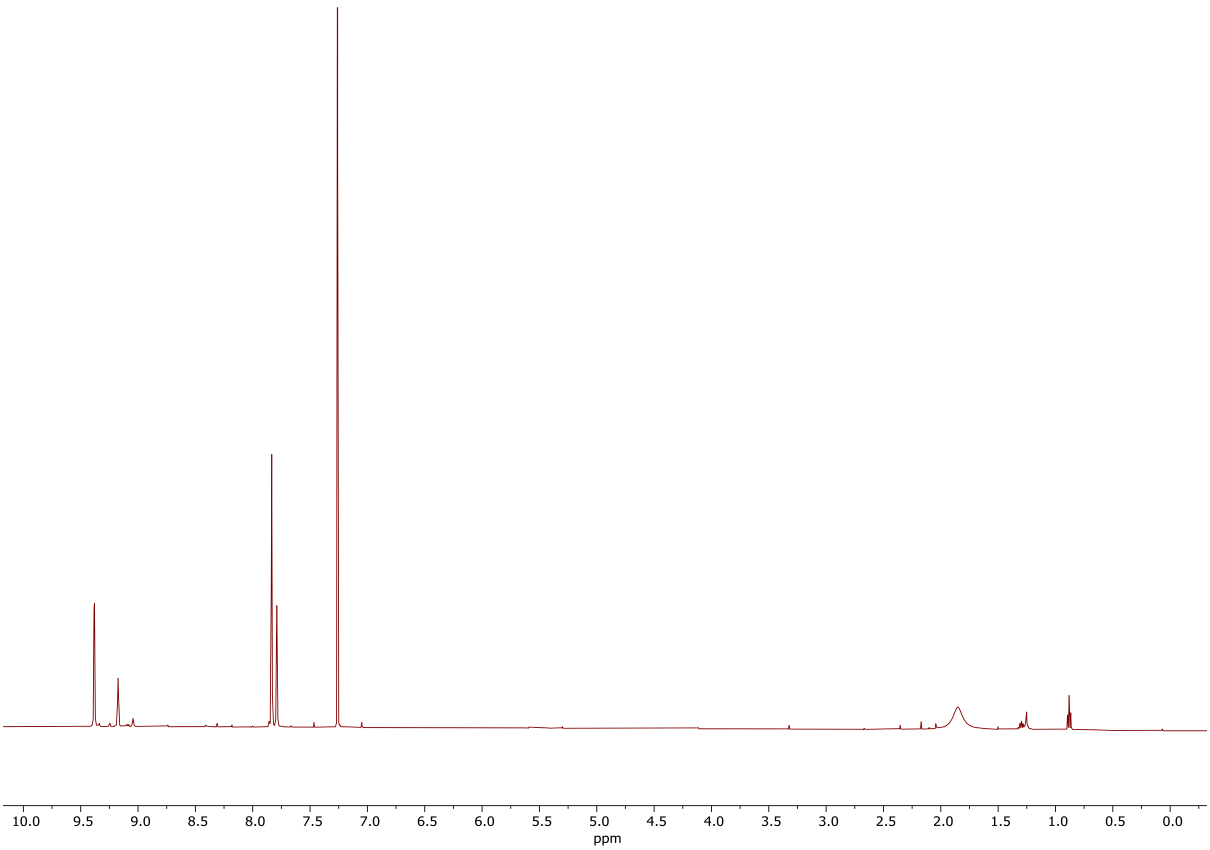

Figure S70. ^{1}H NMR spectrum of **3·ChB^{2CF3}** ($CDCl_3$, 500 MHz, 298 K).

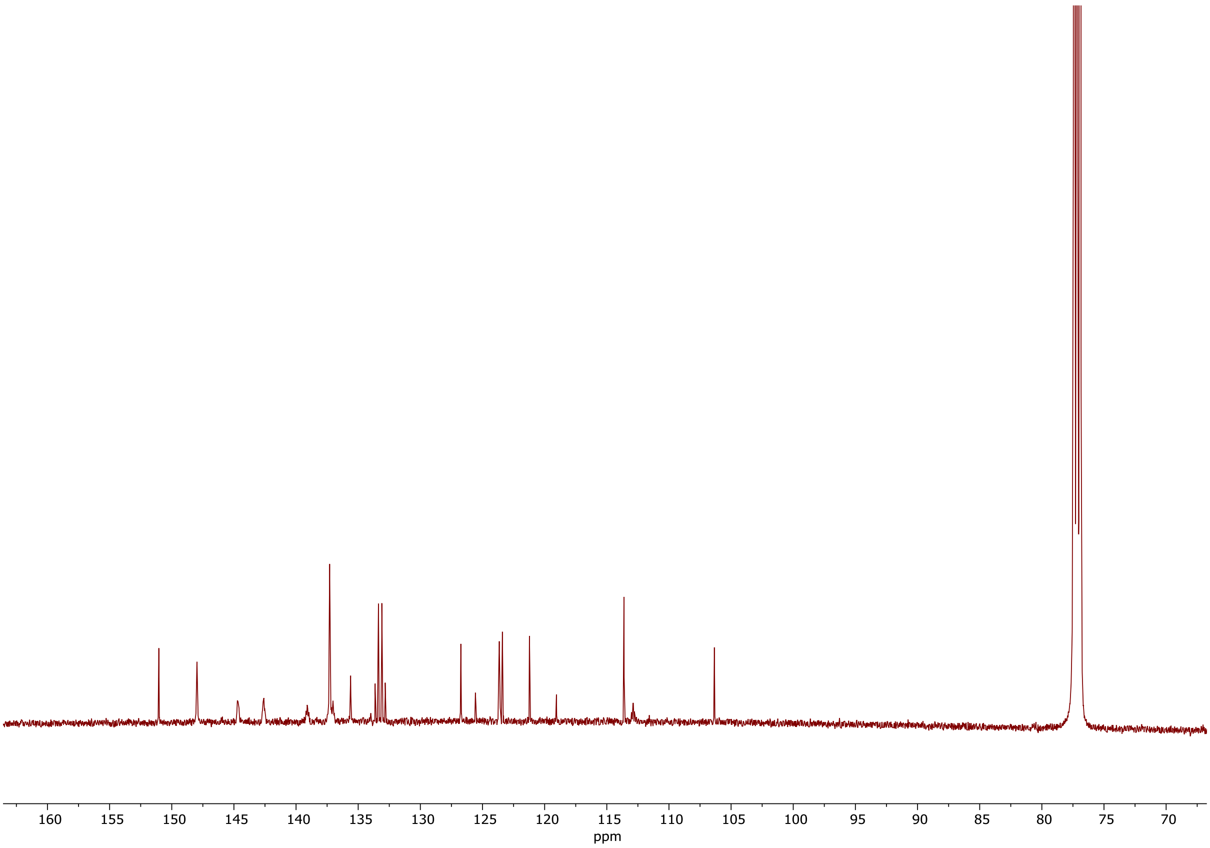

Figure S71. ^{13}C NMR spectrum of **3·ChB^{2CF3}** ($CDCl_3$, 126 MHz, 298 K).

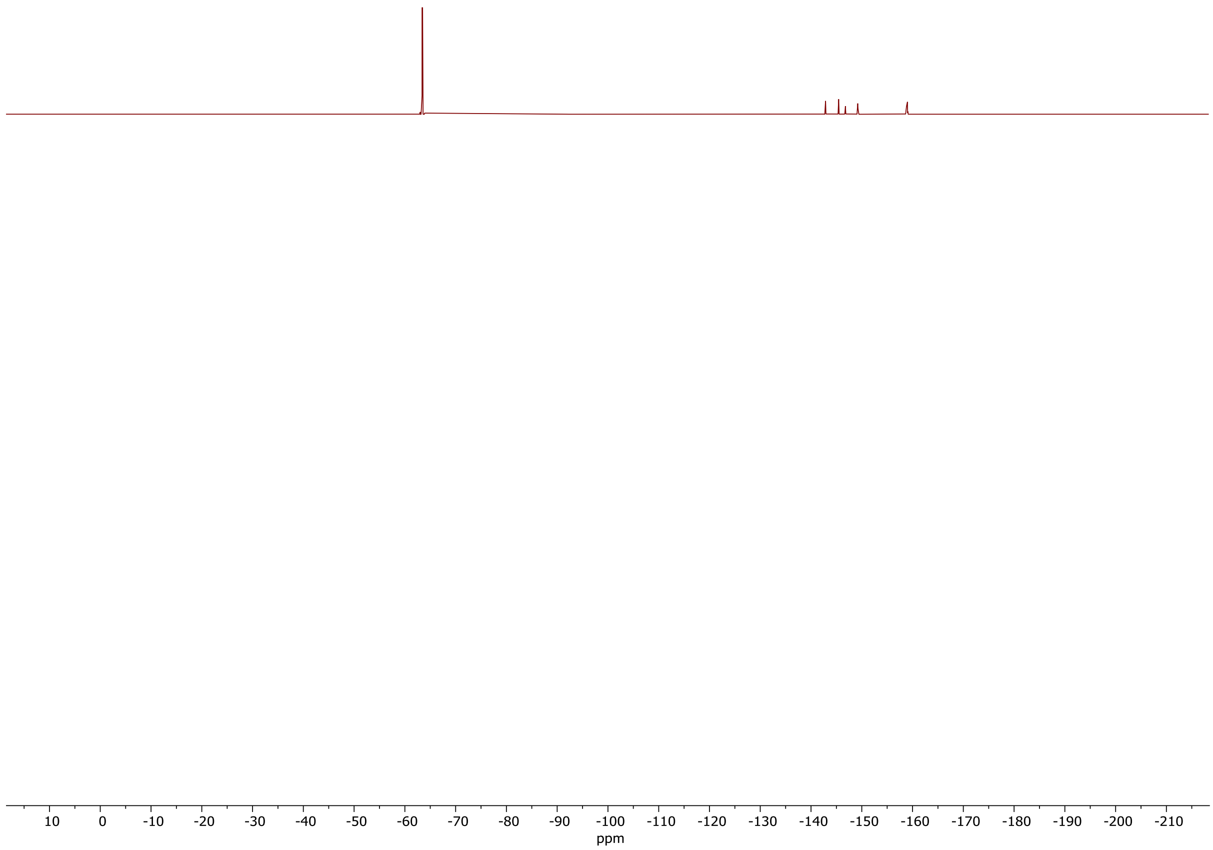

Figure S72. ^{19}F NMR spectrum of **3·ChB^{2CF3}** ($CDCl_3$, 376 MHz, 298 K).

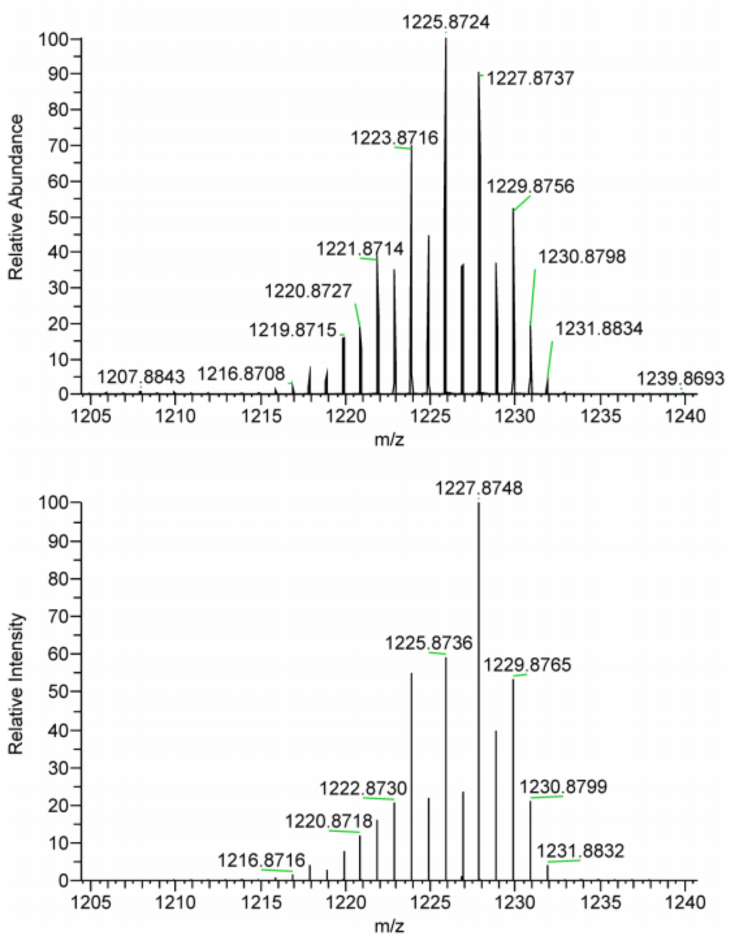

Figure S73. HRESI-MS of **3·ChB^{2CF}**

^{1}H NMR Titration Experiments

Titration protocol: In a typical ^{1}H NMR anion titration experiment, aliquots of anion were added to acetone-d_6, 2.5%-D_2O acetone-d_6 (v/v) or 5%-D_2O acetone-d_6 (v/v) solution of the receptor.

[Host] = 1 mM

[TBACl] or [TBABr] or [TBAI] = 50 mM

Spectra were recorded at 0, 0.2, 0.4, 0.6, 0.8, 1.0, 1.2, 1.4, 1.6, 1.8, 2.0, 2.5, 3.0, 4.0, 5.0, 7.0 and 10 equivalents.

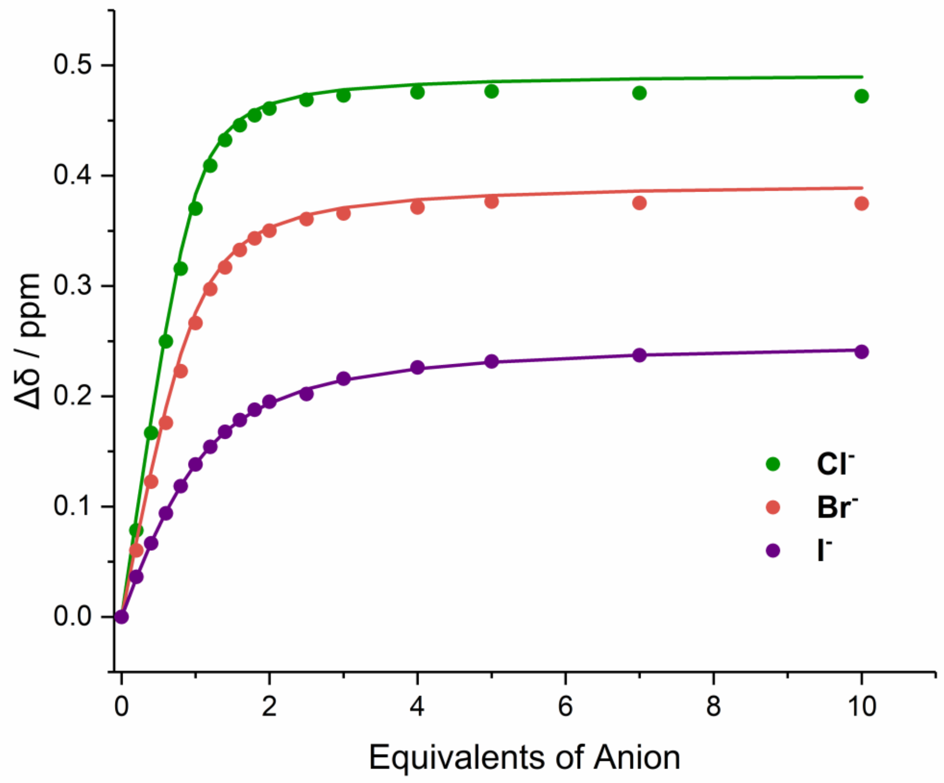

Figure S74. Anion titration binding curve for **1·XB^{Ph}**. Fitted binding isotherm is indicated by line, circles indicate experimental data.

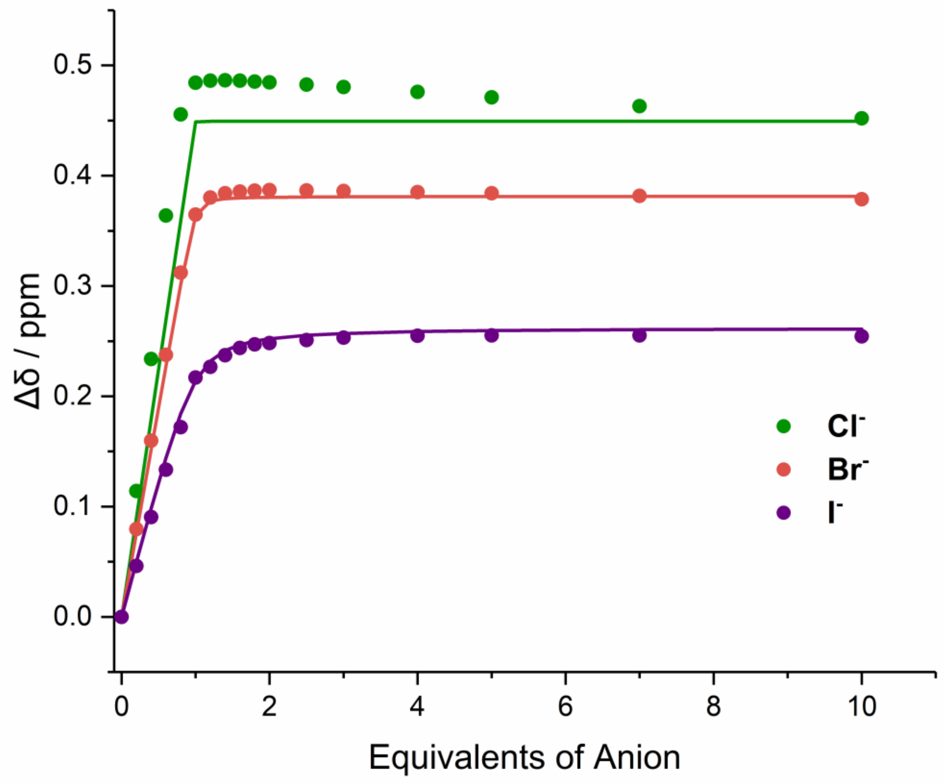

Figure S75. Anion titration binding curve for **1·XB**^{PFB}. Fitted binding isotherm is indicated by line, circles indicate experimental data.

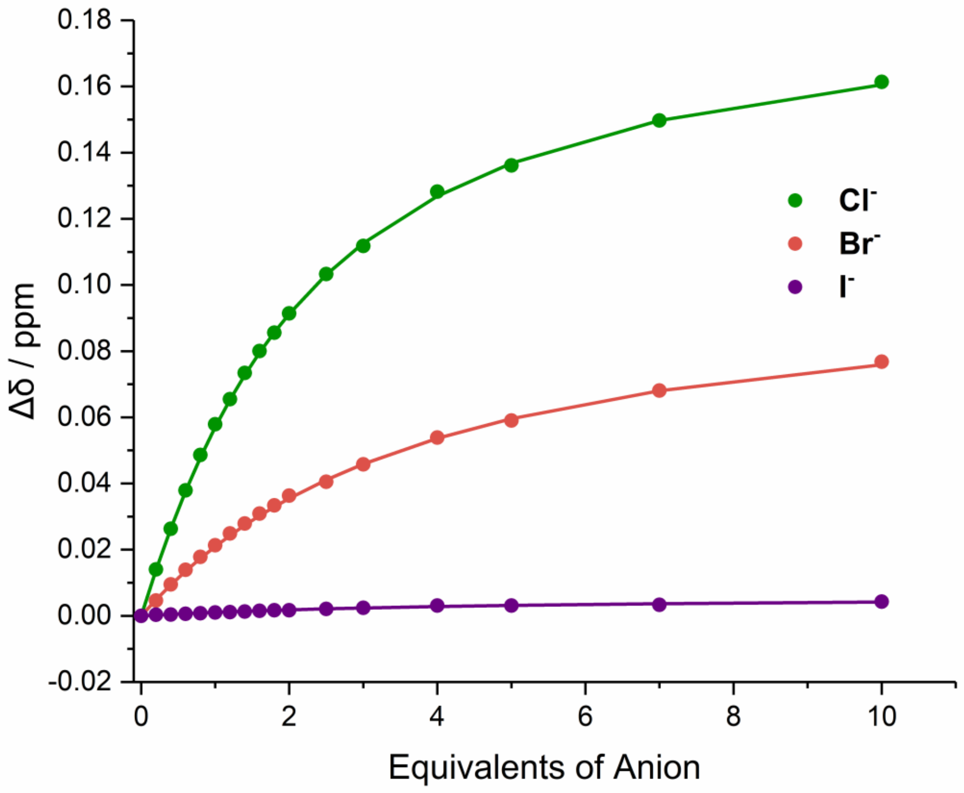

Figure S77. Anion titration binding curve for **1·ChB**$^{\mathbf{Ph}}$. Fitted binding isotherm is indicated by line, circles indicate experimental data.

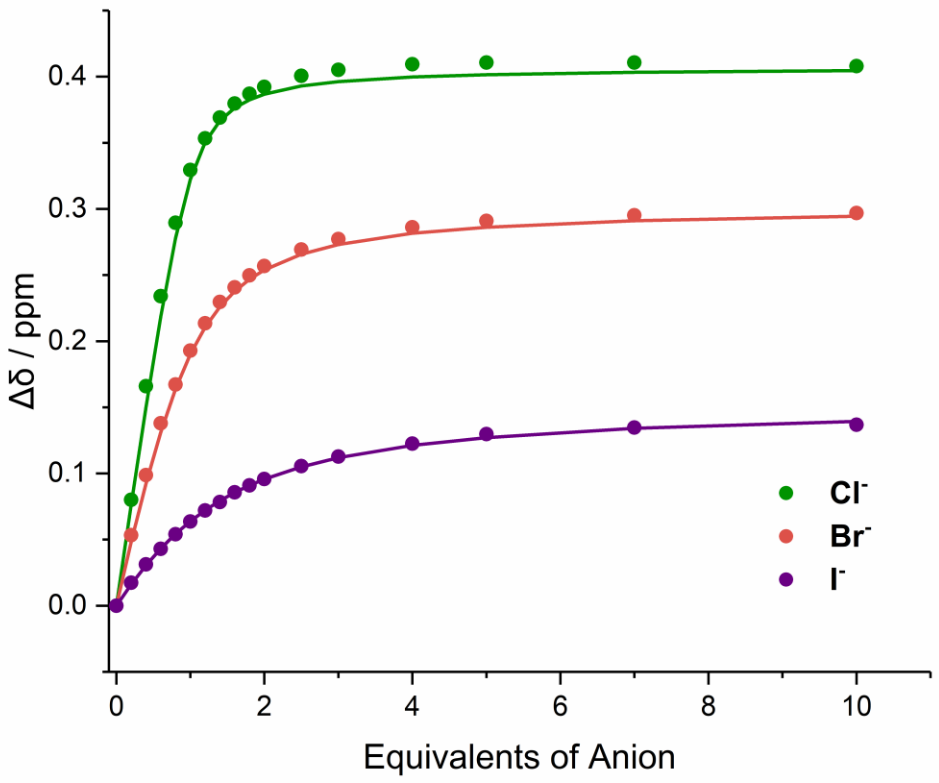

Figure S78. Anion titration binding curve for **1·ChB**^{PFB}. Fitted binding isotherm is indicated by line, circles indicate experimental data.

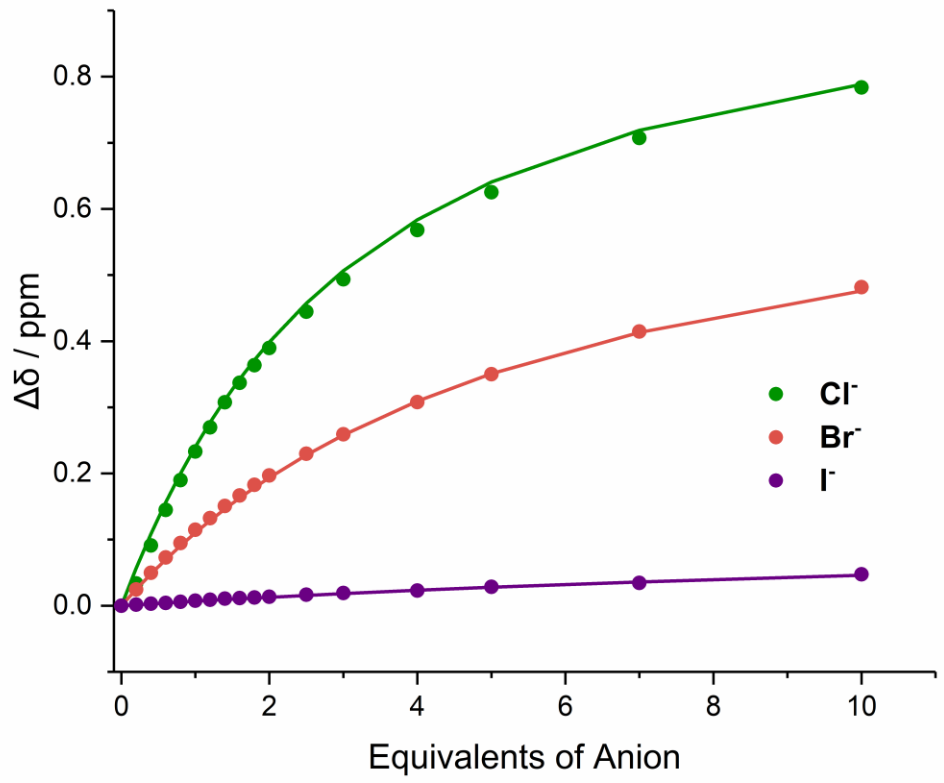

Figure S79. Anion titration binding curve for **1·HB^{Ph}**. Fitted binding isotherm is indicated by line, circles indicate experimental data.

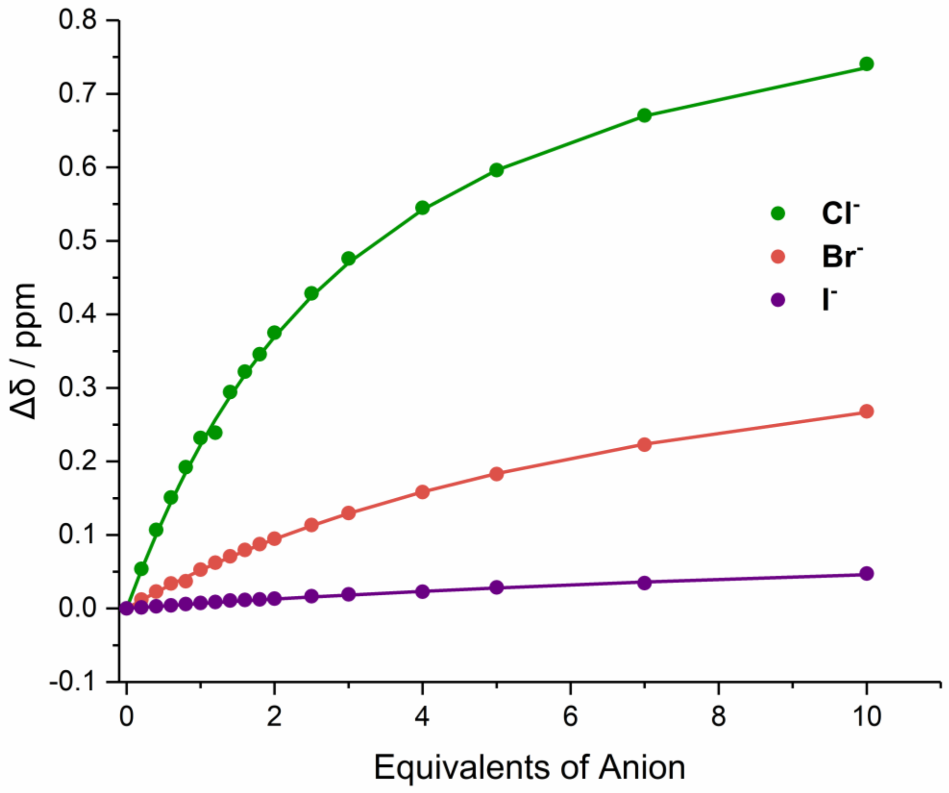

Figure S80. Anion titration binding curve for **1·HB^{PFB}**. Fitted binding isotherm is indicated by line, circles indicate experimental data.

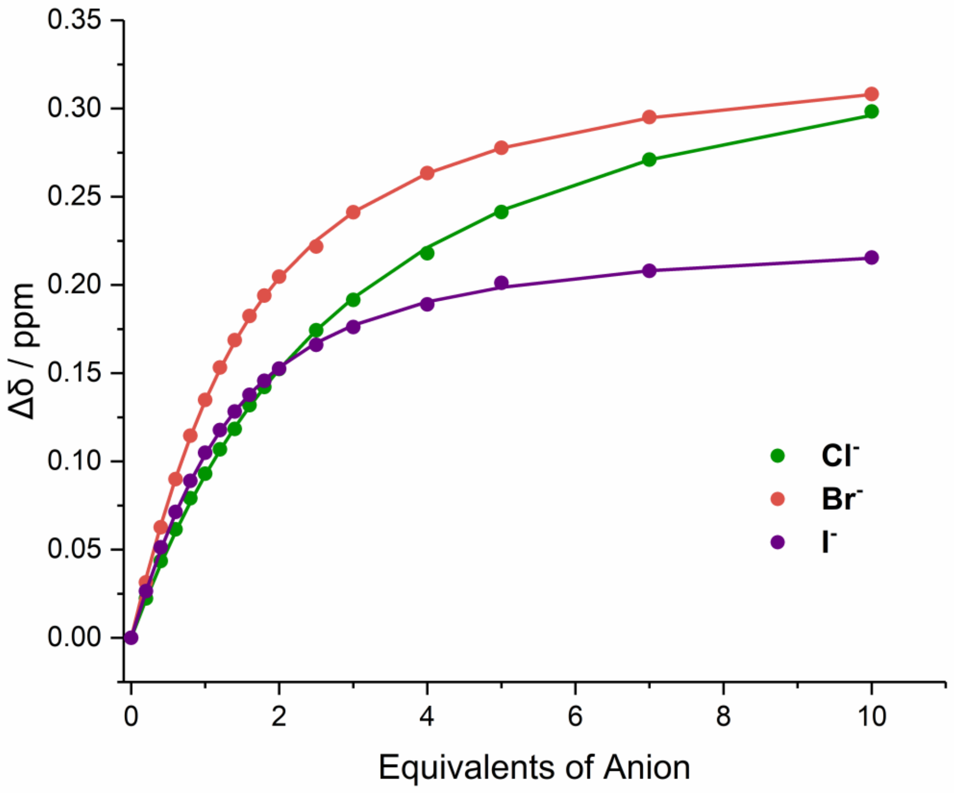

Figure S81. Anion titration binding curve for **1·XB^{Ph}** (2.5% D_2O). Fitted binding isotherm is indicated by line, circles indicate experimental data.

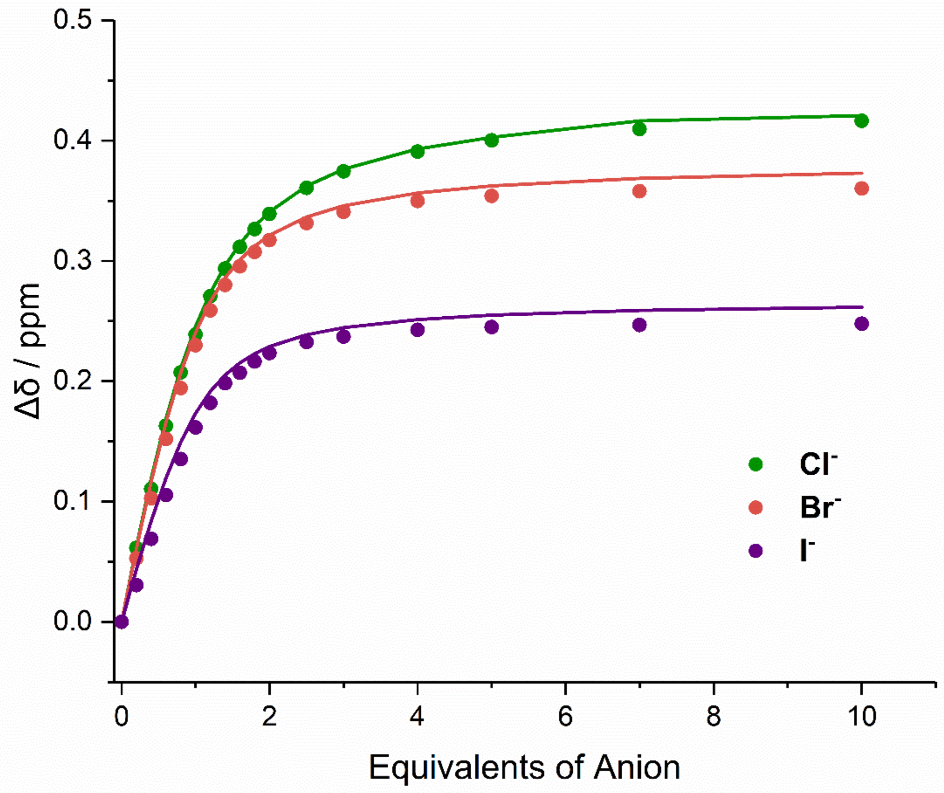

Figure S82. Anion titration binding curve for **1·XB**^{PFB} (2.5% D_2O). Fitted binding isotherm is indicated by line, circles indicate experimental data.

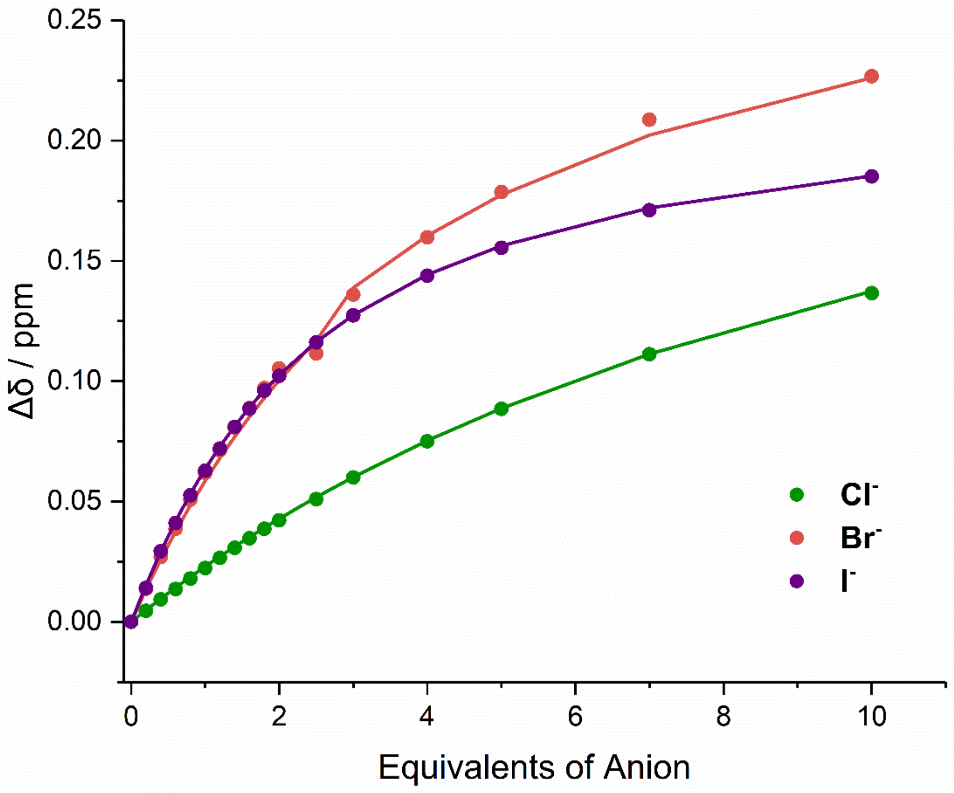

Figure S83. Anion titration binding curve for **1·XB^{Ph}** (5.0% D_2O). Fitted binding isotherm is indicated by line, circles indicate experimental data.

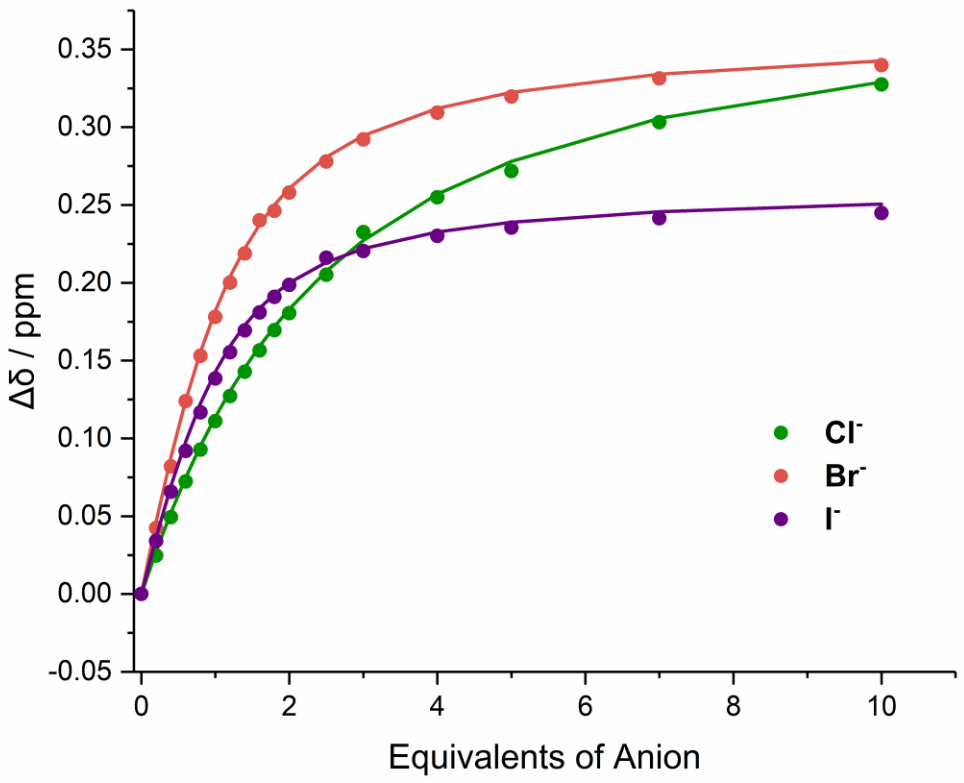

Figure S84. Anion titration binding curve for **1·XB^{PFB}** (5.0% D_2O). Fitted binding isotherm is indicated by line, circles indicate experimental data.

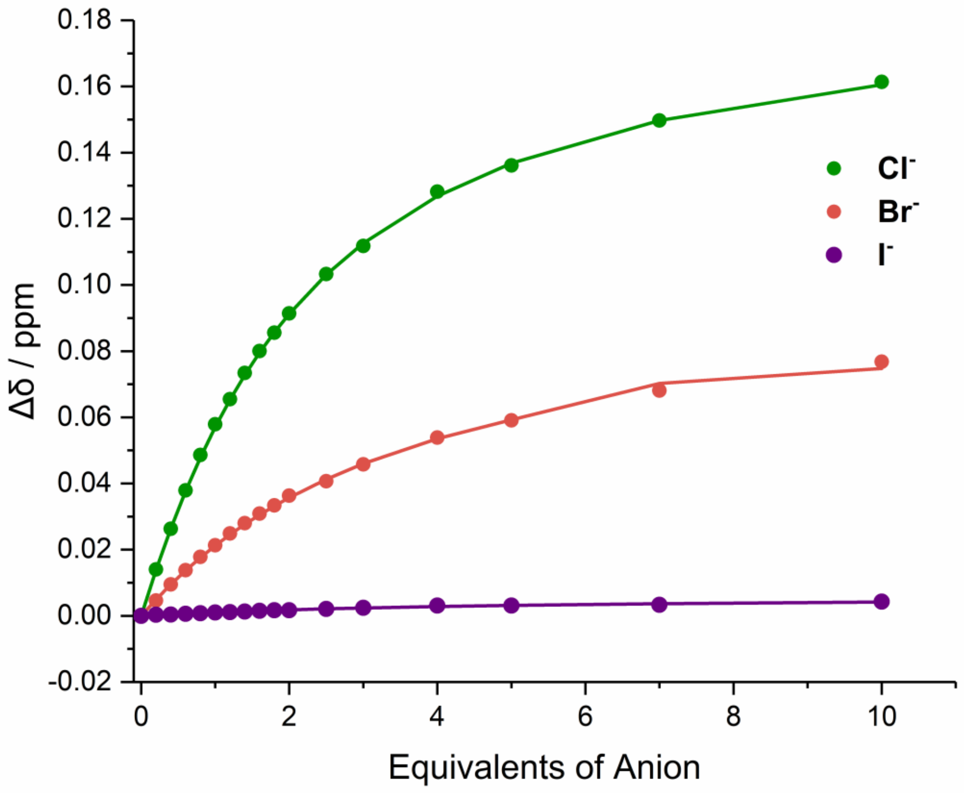

Figure S85. Anion titration binding curve for **1·ChB^Ph**. Fitted binding isotherm is indicated by line, circles indicate experimental data.

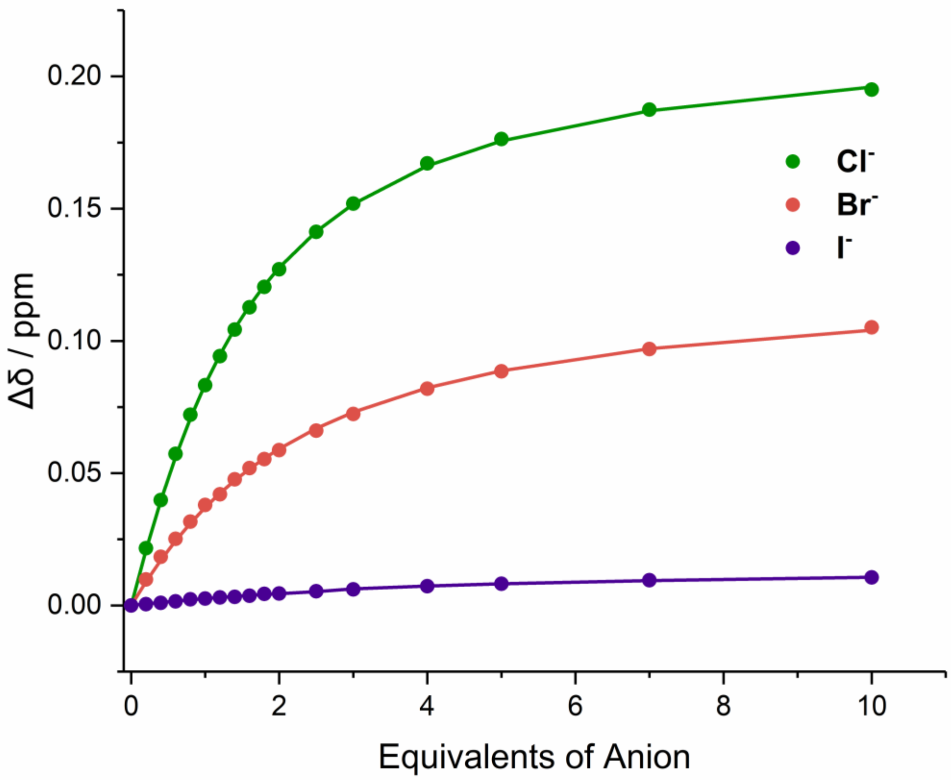

Figure S86. Anion titration binding curve for **2·ChB^{mF}**. Fitted binding isotherm is indicated by line, circles indicate experimental data.

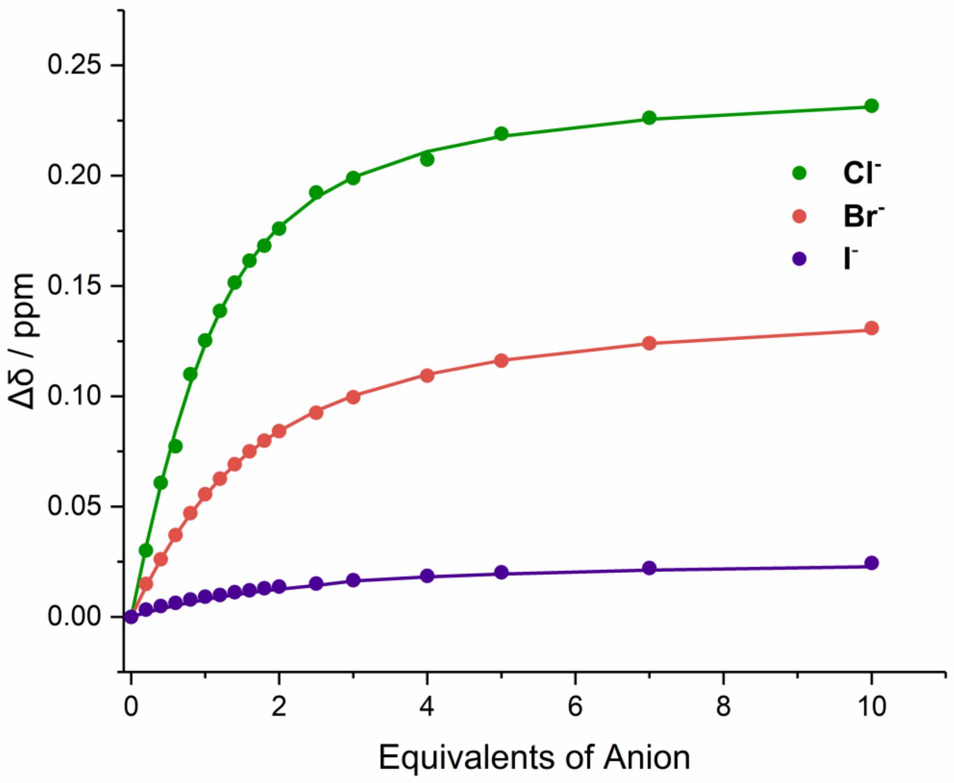

Figure S87. Anion titration binding curve for **2·ChB^{2F}**. Fitted binding isotherm is indicated by line, circles indicate experimental data.

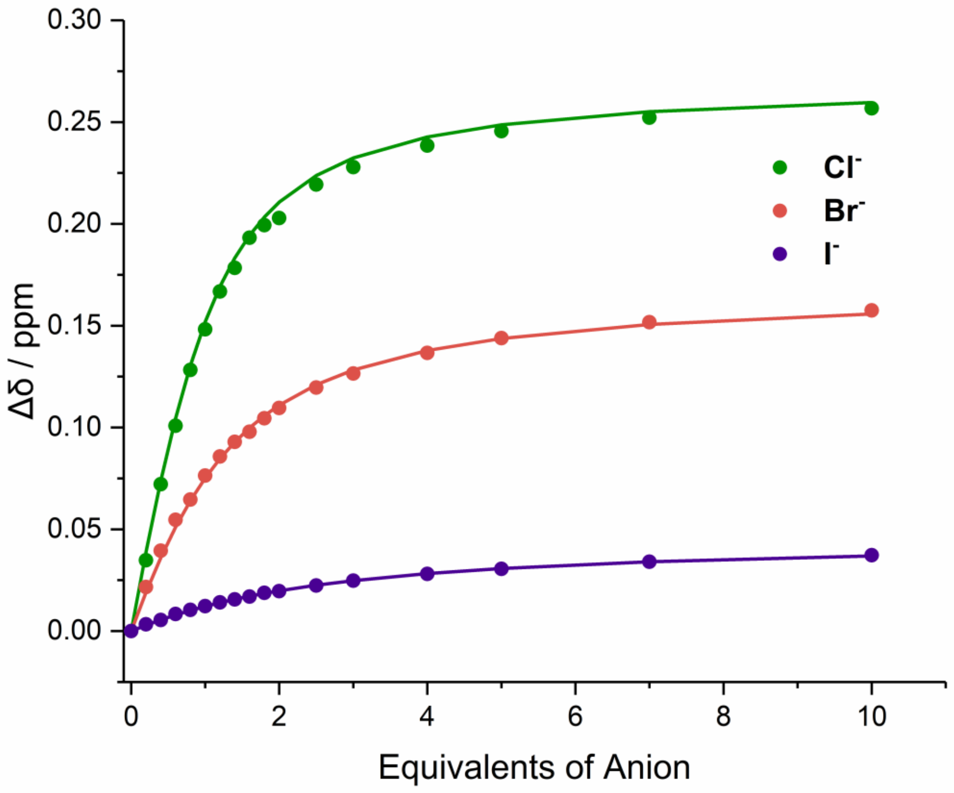

Figure S88. Anion titration binding curve for **2·ChB^{3F}**. Fitted binding isotherm is indicated by line, circles indicate experimental data.

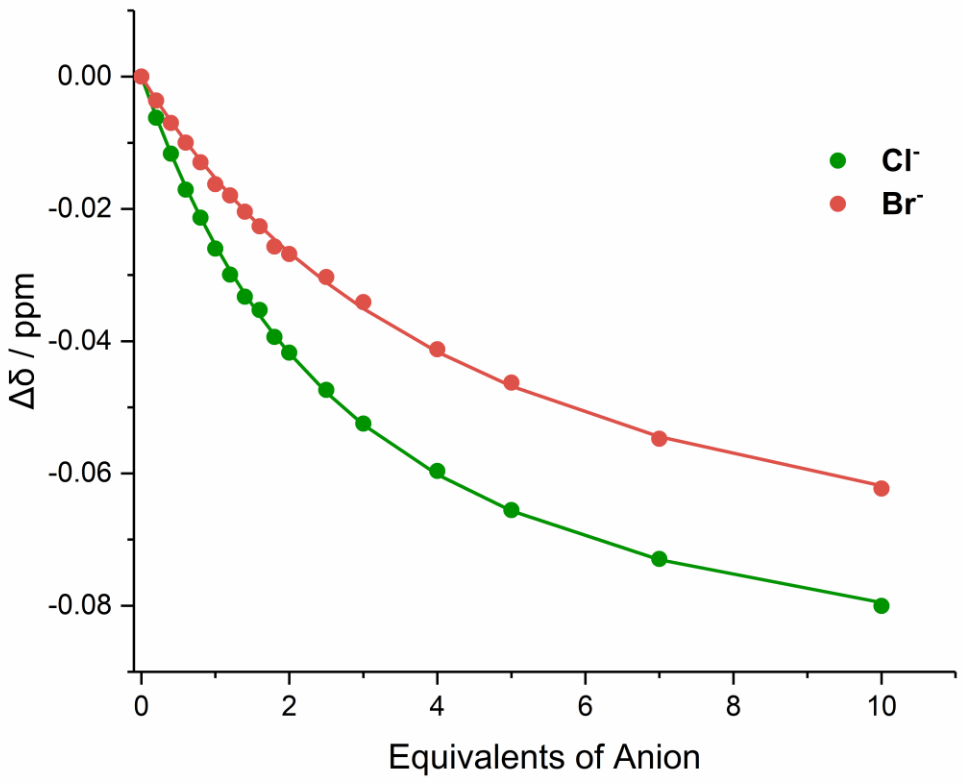

Figure S89. Anion titration binding curve for **3·ChB^{pOMe}**. Fitted binding isotherm is indicated by line, circles indicate experimental data.

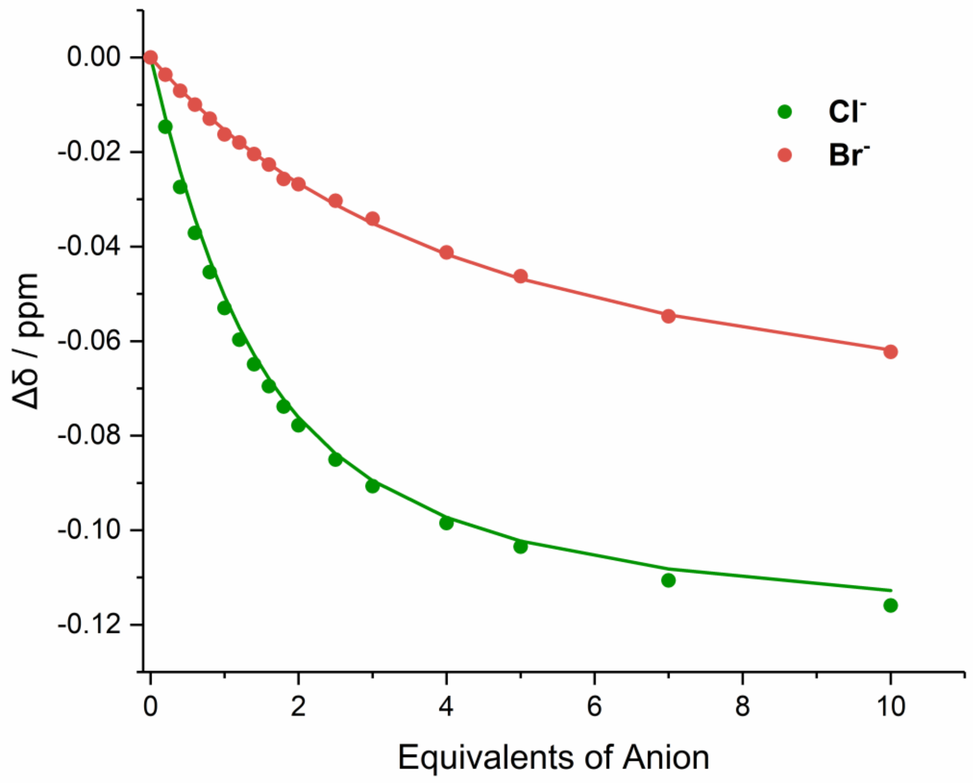

Figure S90. Anion titration binding curve for **3·ChB^pMe^**. Fitted binding isotherm is indicated by line, circles indicate experimental data.

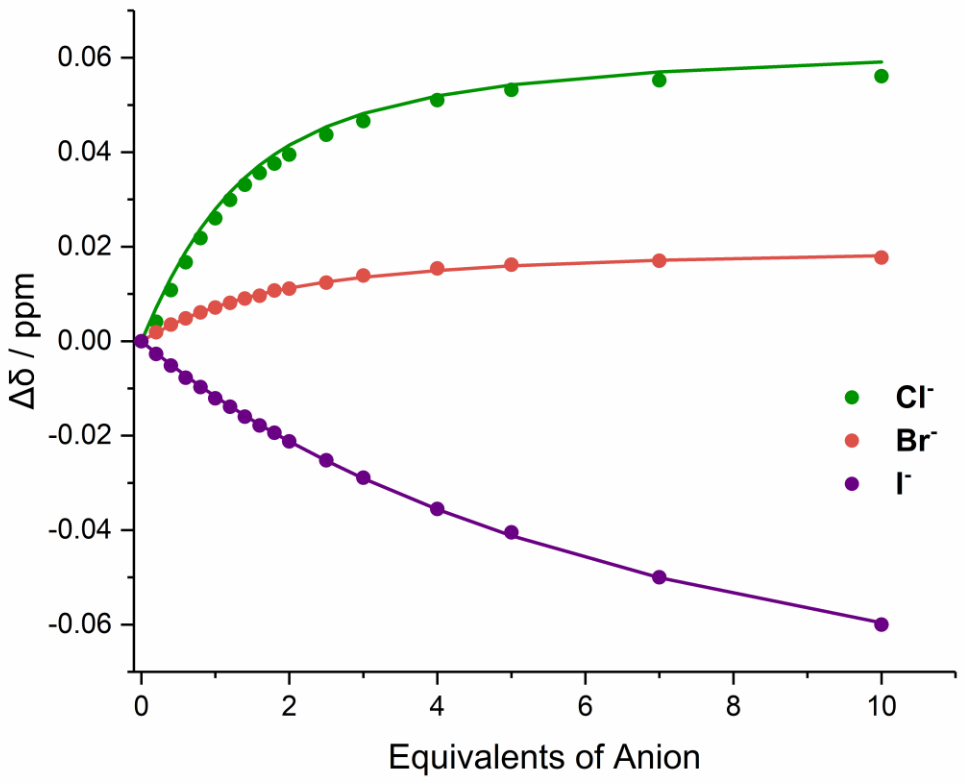

Figure S91. Anion titration binding curve for **3·ChB^{Ph}**. Fitted binding isotherm is indicated by line, circles indicate experimental data.

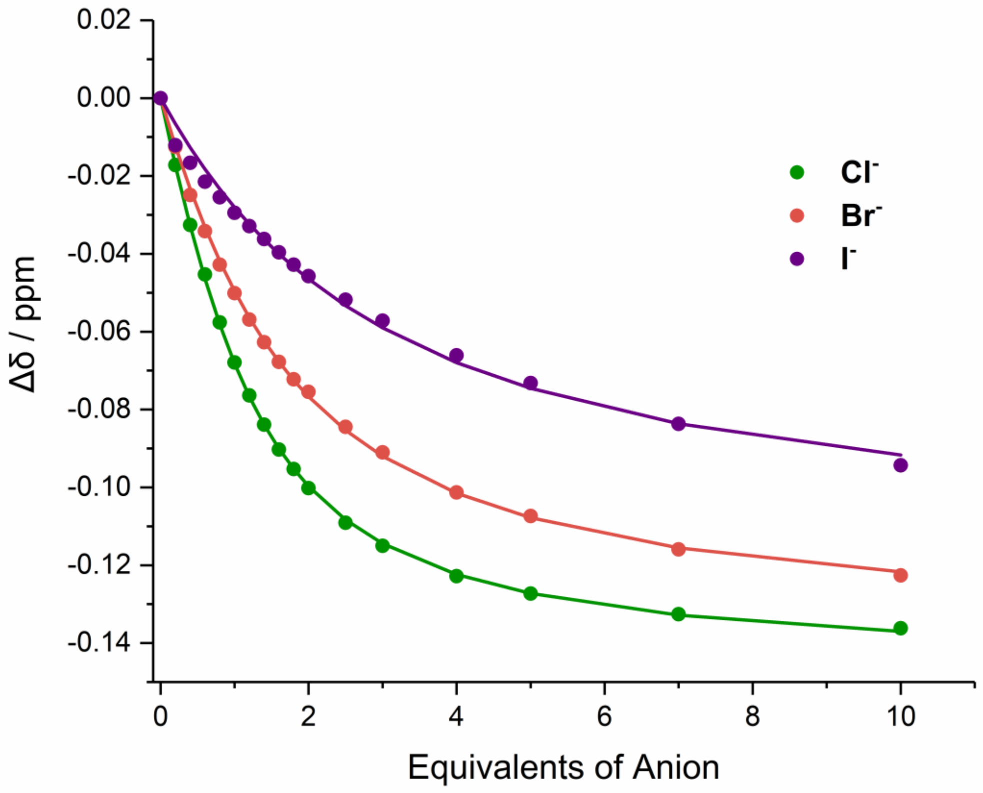

Figure S92. Anion titration binding curve for **3·ChB**^{pF}. Fitted binding isotherm is indicated by line, circles indicate experimental data.

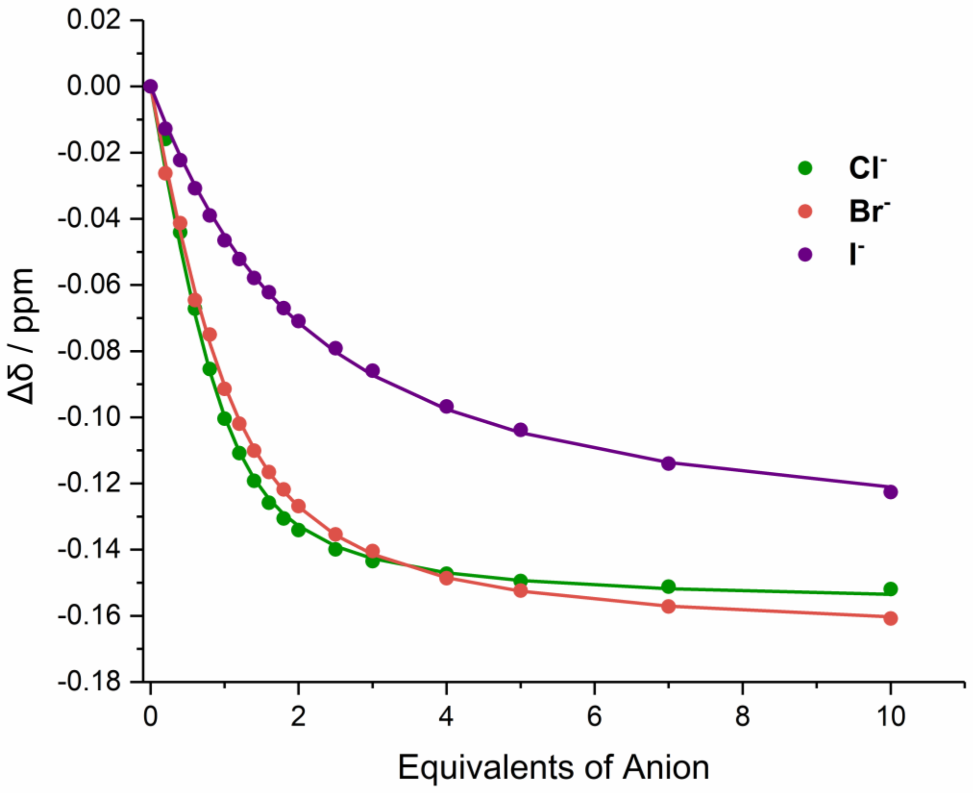

Figure S93. Anion titration binding curve for **3·ChB^{pCl}**. Fitted binding isotherm is indicated by line, circles indicate experimental data.

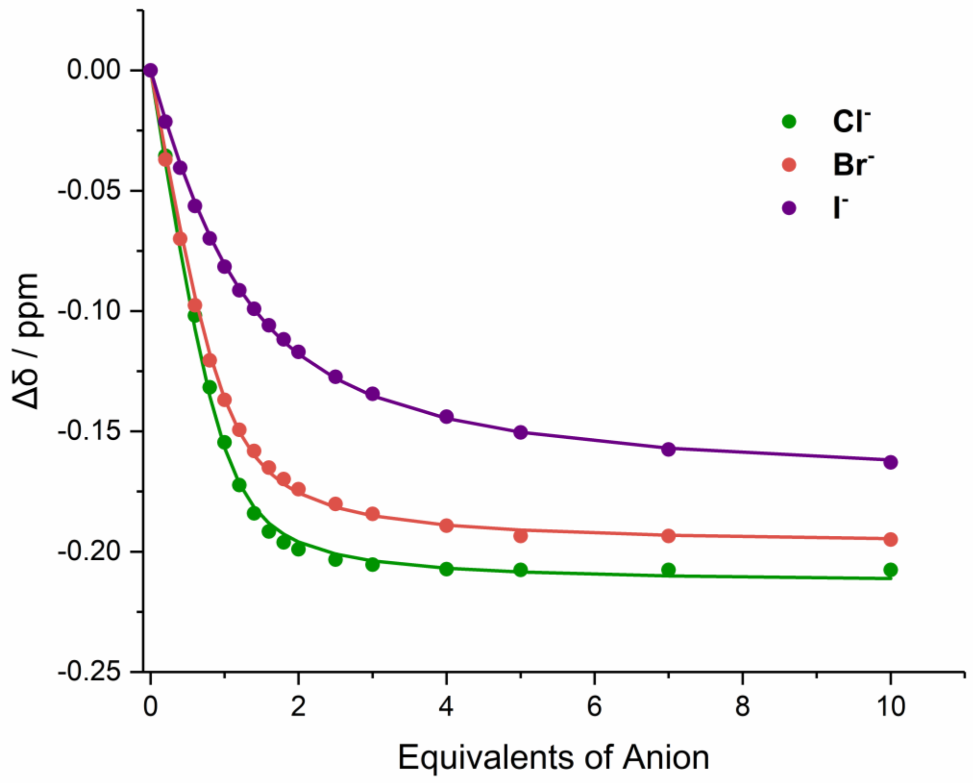

Figure S94. Anion titration binding curve for **3·ChB^{mF}**. Fitted binding isotherm is indicated by line, circles indicate experimental data.

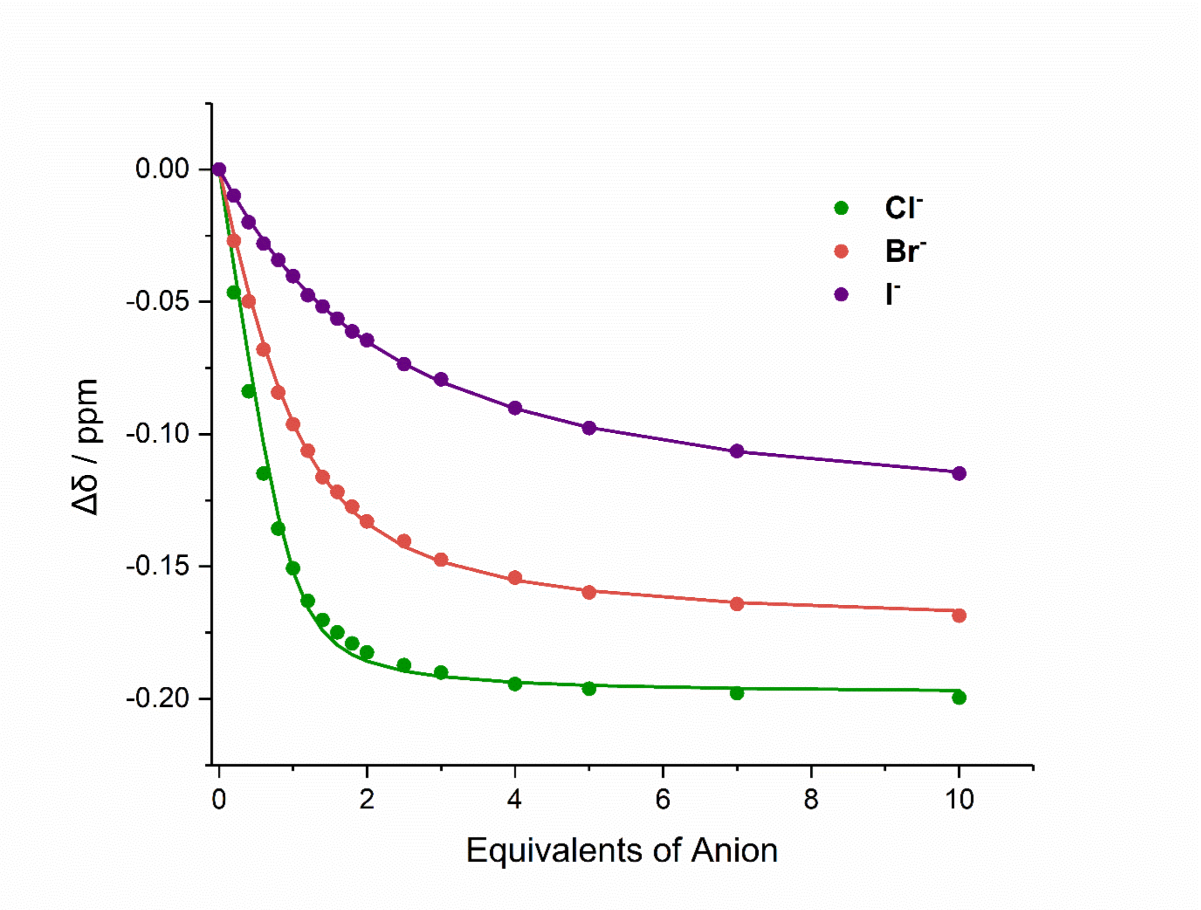

Figure S95. Anion titration binding curve for **3·ChB^{mCF3}**. Fitted binding isotherm is indicated by line, circles indicate experimental data.

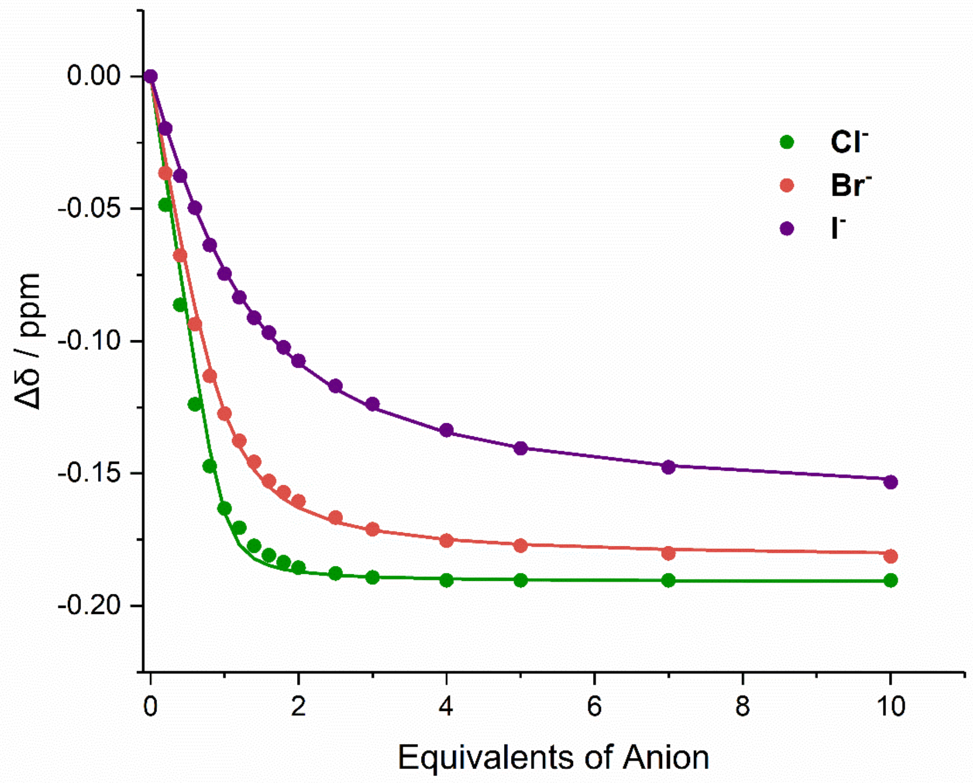

Figure S96. Anion titration binding curve for **3·ChB^{pCF3}**. Fitted binding isotherm is indicated by line, circles indicate experimental data.

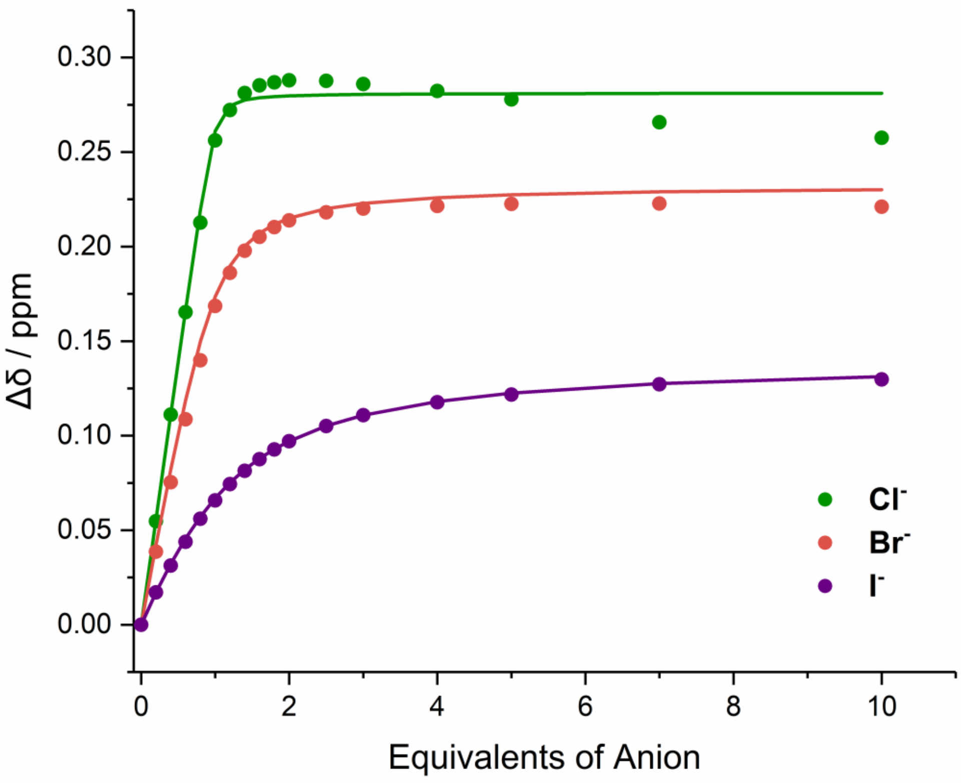

Figure S97. Anion titration binding curve for **3·ChB^{2F}**. Fitted binding isotherm is indicated by line, circles indicate experimental data.

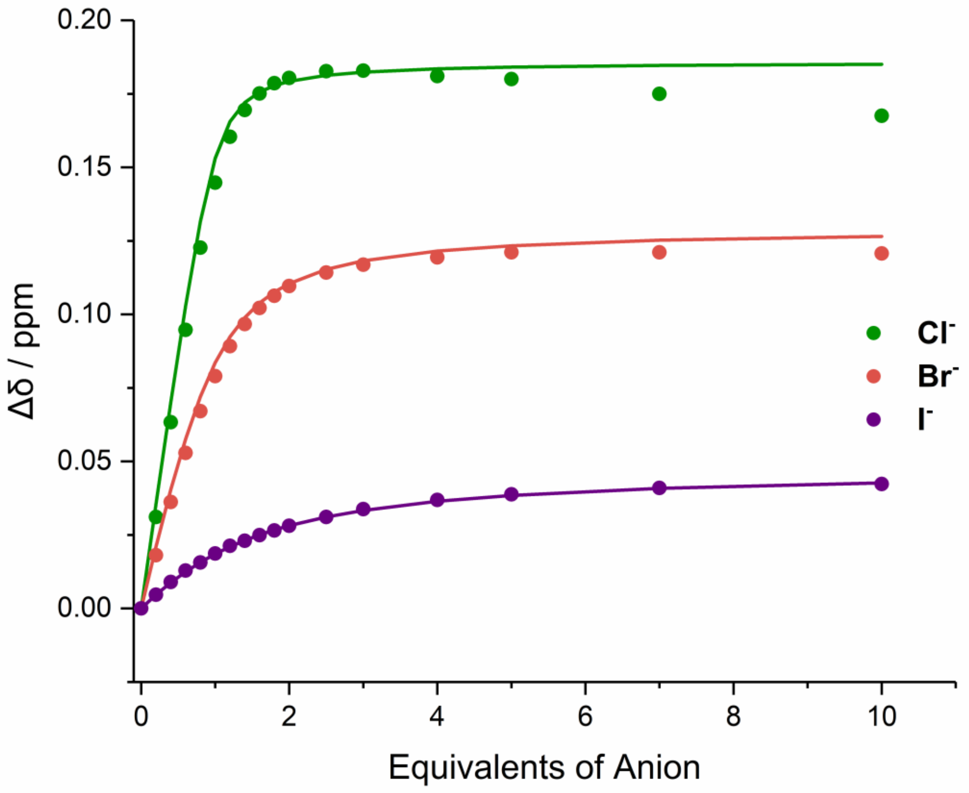

Figure S98. Anion titration binding curve for **3·ChB^{3F}**. Fitted binding isotherm is indicated by line, circles indicate experimental data.

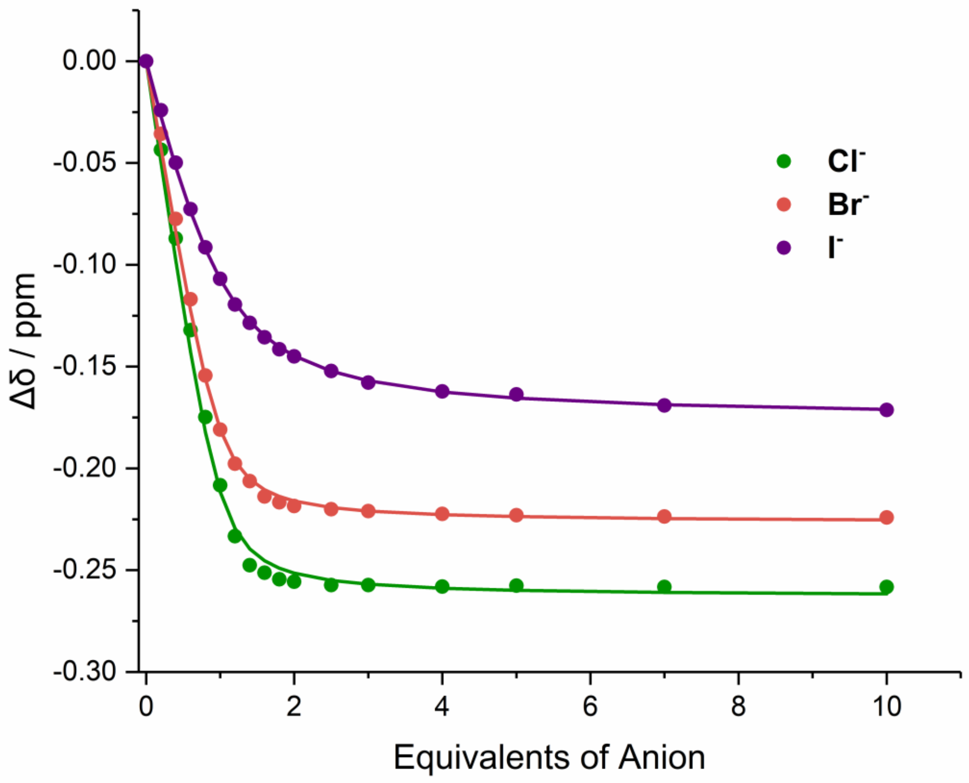

Figure S99. Anion titration binding curve for **3·ChB^{2CF3}**. Fitted binding isotherm is indicated by line, circles indicate experimental data.

Crystal Structure Determination

Single crystals of **1·ChB^{PFP}**, **1·XB^{PFP}** ,**1·HB^{PFP}**, **3·ChB^{pCl}**, **3·ChB^{2F}**, **3·ChB^{3F}** suitable for X-ray analysis were each coated with Paratone-N oil, suspended on a 200 μm MiTeGen loop, and placed in a cold gaseous nitrogen stream on an Oxford Diffraction Supernova X-ray diffractometer performing φ- and ω-scans at 150(2) K. Diffraction[5] intensities were measured using graphite monochromated Cu Kα radiation (1.54184 Å). Data collection, indexing, initial cell refinements, frame integration, final cell refinements and absorption corrections were accomplished using the program CrysAlisPro.† Scattering factors and anomalous dispersion corrections were taken from the International Tables for X-ray Crystallography. All structures were solved by direct methods and refined against F2. All hydrogen atoms were included into the model at geometrically calculated positions and refined using a riding model. Details of the data quality and a summary of the residual values for the refinements are listed in Tables S1 and S2. Figures of the crystal structures have been created using the open-source PyMOL Molecular Graphics System.

Deposition Number(s) 2081712 (for **1·ChB^{PFP}**) and 2081709 (for **1·XB^{PFP}**) and 2081707 (for **1·HB^{PFP}**) and 2081718 (for **3·ChB^{pCl}**) and 2081716 (for **3·ChB^{2F}**) and 2081717 (for **3·ChB^{3F}**) contains the supplementary crystallographic data for this paper. These data are provided free of charge by the joint Cambridge Crystallographic Data Centre and Fachinformationszentrum Karlsruhe Access Structures service www.ccdc.cam.ac.uk/structures.

† CrysAlisPRO, Oxford Diffraction /Agilent Technologies UK Ltd, Yarnton, England.

Compound	1·ChB^{PFP}	1·XB^{PFP}	1·HB^{PFP}
Formula	C23 H9 F10 N7 Te2	C21 H3 F10 I2 N7	C21 H5 F10 N7
Formula Weight	828.55	797.09	545.30
a (Å)	8.6071(1)	14.8642(1)	22.0691(3)
b (Å)	14.5248(1)	8.9212(1)	11.2961(1)
c (Å)	21.4109(1)	18.5412(2)	16.3807(2)
α (º)	90	90	90
β (º)	90	101.7937(10)	100.6364(13)
γ (º)	90	90	90
Unit cell volume (Å^3)	2676.71(4)	2406.78(4)	4012.46(8)
Crystal system	orthorhombic	monoclinic	monoclinic
Space group	P 21 21 21	P 21/n	C 2/c
Z	4	4	8
Temperature (K)	150	150	150
Radiation Type	copper	copper	copper
λ (Å)	1.54180	1.54180	1.54180
Reflections (all)	11151	17390	15395
Reflections (unique)	4156	4981	5537
R_{int}	0.020	0.047	0.034
R[I > 2σ(*I*)]	0.0236	0.0537	0.0311
wR(F^2) (all)	0.0599	0.1380	0.0865
S	0.948	1.032	0.979

Table S1. Selected Crystallographic and Refinement Data for X-ray Crystal Structures of **1·ChB^{PFP}, 1·XB^{PFP}** and **1·HB^{PFP}**.

**Compound**	**3·ChB^{2F}**	**3·ChB^{3F}**	**3·ChB^{pCl}**
**Formula**	C33 H9 F14 N7 Te2	C33 H7 F16 N7 Te2	C33 H11 Cl2 F10 N7 Te2
**Formula Weight**	1024.65	1060.63	1021.58
**a (Å)**	13.5278(1)	13.6915(2)	7.5401(3)
**b (Å)**	16.0201(2)	15.9855(2)	14.6361(4)
**c (Å)**	15.4818(2)	15.5790(2)	17.0146(5)
***α* (º)**	90	90	66.076(3)
***β* (º)**	101.5223(12)	102.3561(12)	79.912(3)
***γ* (º)**	90	90	86.479(3)
**Unit cell volume (Å^3)**	3287.55(7)	3330.72(8)	1689.74(10)
**Crystal system**	monoclinic	monoclinic	triclinic
**Space group**	P 21/c	P 21/c	P -1
**Z**	4	4	2
**Temperature (K)**	150	150	150
**Radiation Type**	copper	copper	copper
**λ (Å)**	1.54180	1.54180	1.54180
**Reflections (all)**	14900	13990	16591
**Reflections (unique)**	6883	6944	6985
**R_{int}**	0.032	0.027	0.037
**R[I > 2σ(*I*)]**	0.0458	0.0453	0.0473
**wR(*F*^{2}) (all)**	0.1225	0.1257	0.1169
**S**	1.014	1.017	0.952

Table S2. Selected Crystallographic and Refinement Data for X-ray Crystal Structures of **3·ChB^{2F}** , **3·ChB^{3F}** and **3·ChB^{pCl}**.

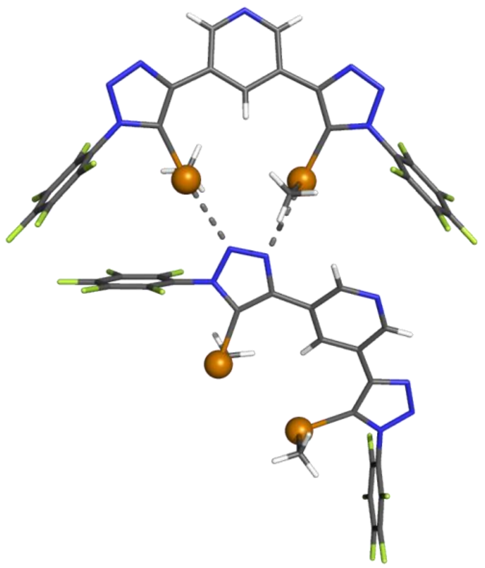

Figure S101. X-ray crystallographic structures of **1·ChB^{PFP},**,ChB interactions in the solid state, represented by dashed lines. Grey = carbon, blue = nitrogen, white hydrogen, green = fluorine, orange = tellurium.

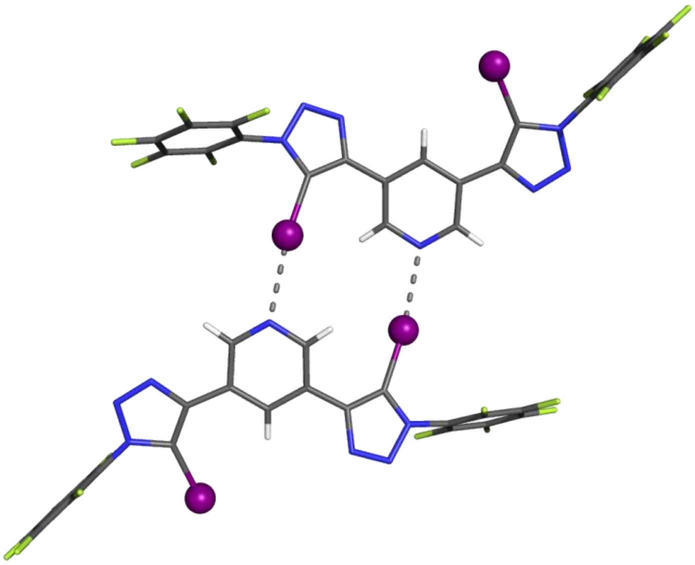

Figure S102. X-ray crystallographic structures of **1·XB^{PFP}** showing the formation of XB interactions in the solid state, represented by dashed lines. Grey = carbon, blue = nitrogen, white hydrogen, green = fluorine, purple = iodine.

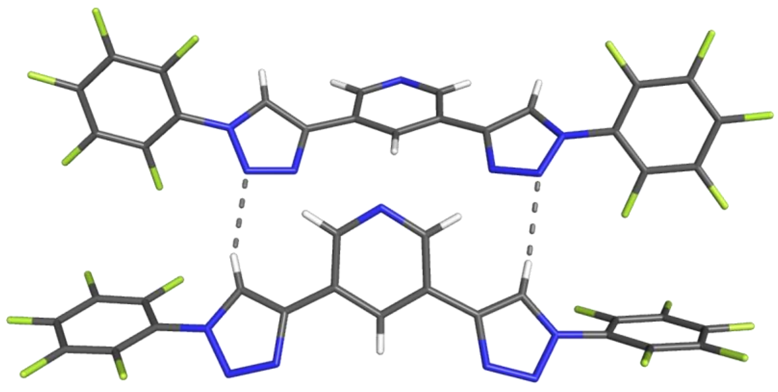

Figure S103. X-ray crystallographic structures of **1·HB**$^{\mathbf{PFP}}$, showing the formation of HB interactions in the solid state, represented by dashed lines. Grey = carbon, blue = nitrogen, white hydrogen, green = fluorine.

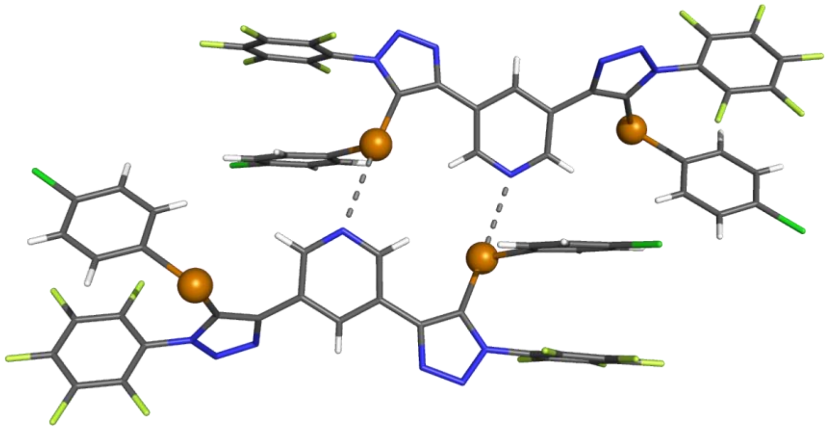

Figure S104. X-ray crystallographic structures of **3·ChB^{pCl}** ChB interactions in the solid state, represented by dashed lines. Grey = carbon, blue = nitrogen, white hydrogen, green = fluorine, dark green = chlorine, orange = tellurium.

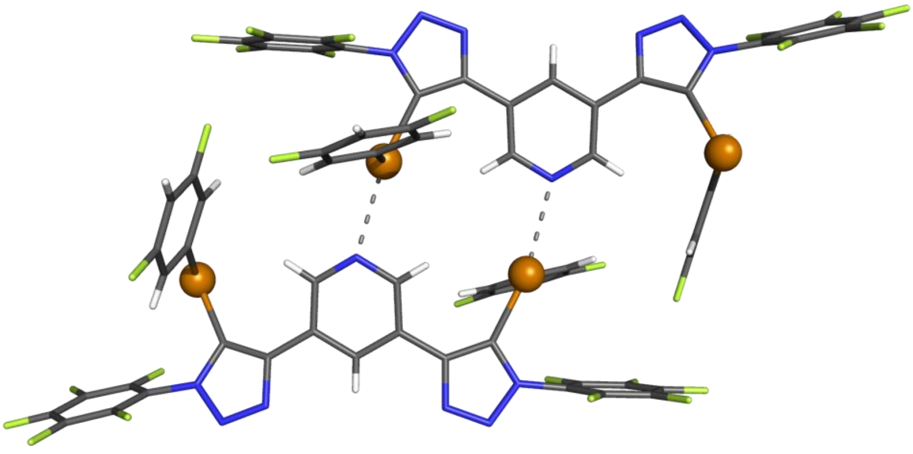

Figure S105. X-ray crystallographic structures of **3·ChB^{2F}** showing the formation of ChB interactions in the solid state, represented by dashed lines. Grey = carbon, blue = nitrogen, white hydrogen, green = fluorine, orange = tellurium.

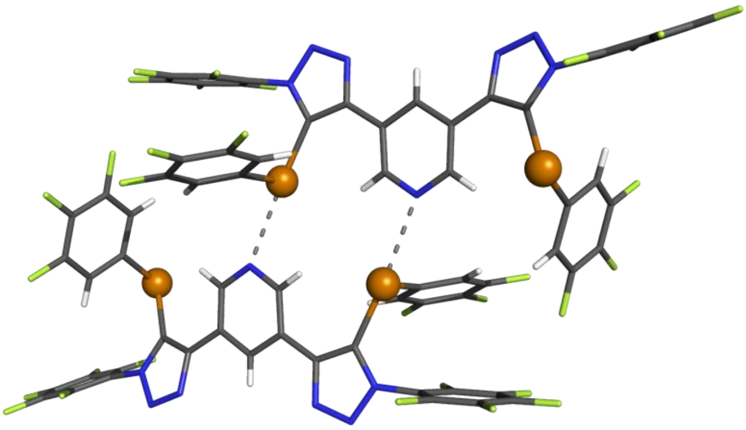

Figure S106. X-ray crystallographic structures of **3·ChB^{3F}** showing the formation of ChB interactions in the solid state, represented by dashed lines. Grey = carbon, blue = nitrogen, white hydrogen, green = fluorine, orange = tellurium.

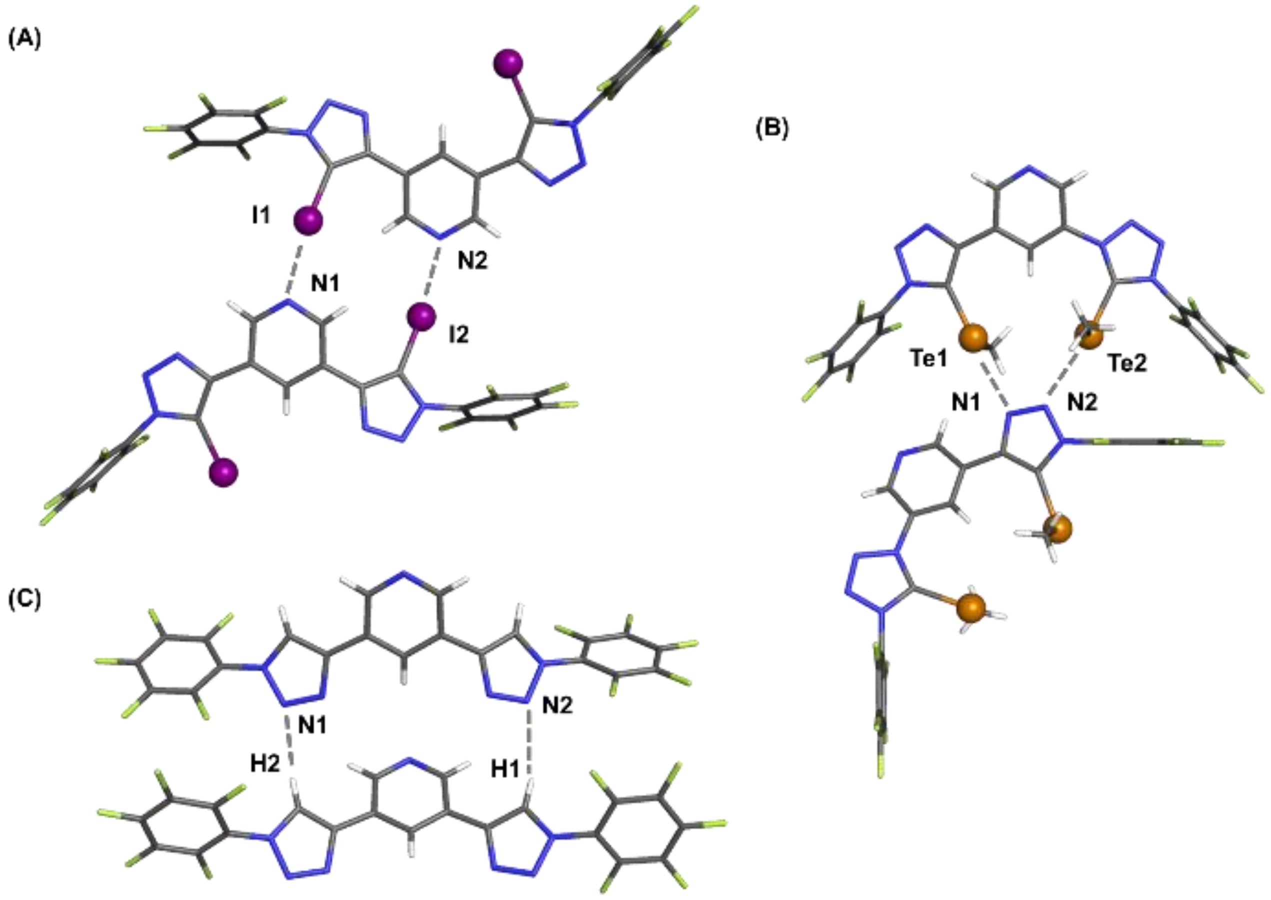

Figure S107. X-ray crystallographic structures of (A) **1·XB^{PFP}** (B) **1·ChB^{PFP}** and (C) **1·HB^{PFP}**. Dashed lines indicate XB, ChB and HB interactions respectively. Grey = carbon, blue = nitrogen, white hydrogen, green = fluorine, iodine = purple, orange = tellurium.

Table S3. Selected solid-state contacts for **1·XB^{PFP}**, **1·ChB^{PFP}** and **1·HB^{PFP}**.

	Atom 1	Atom 1	Distance / Å	%vdW	Contact Angle
**1·XB^{PFP}**	I1	N1	2.788	74%	169
	N2	I2	2.788	74%	169
**1·ChB^{PFP}**	Te1	N1	2.938	77%	172
	Te2	N2	3.019	80%	172
**1·HB^{PFP}**	H1	N2	2.614	95%	159
	H2	N1	2.536	92%	170

Representative examples and summarised data

a)

b)

c)

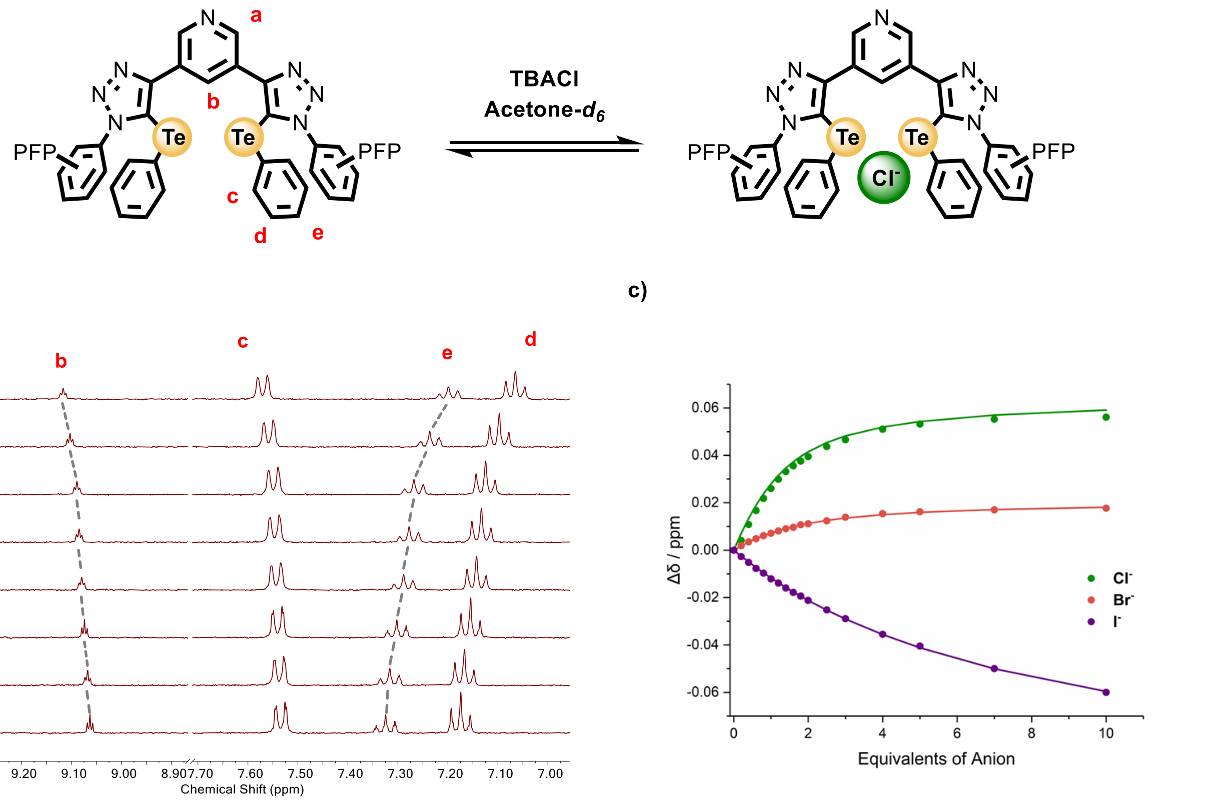

Figure S108. a) Chloride anion binding equilibria for **3·ChB^{Ph}**. b) Stacked ^{1}H NMR titration spectra for **3·ChB^{Ph}** with increasing equivalents of TBACl (acetone-d_6, 500 MHz, 298K). c) Anion Binding titration isotherm for **1·ChB^{PFP}** with chloride, bromide and iodide.

Table S4. Halide anion association constants and calculated binding enhancement factors for phenyl and pefluorophenyl appended ChB, XB and HB acyclic anion receptor systems.

	Anion association constants K_a / M^{-1} [a]									
Anion[b]	Acetone-d_6						2.5 %-D_2O-acetone-d_6		5 %-D_2O-acetone-d_6	
	**1·ChB^{PFP}**	**1·ChB^{Ph}**	**1·XB^{PFP}**	**1·XB^{Ph}**	**1·HB^{PFP}**	**1·HB^{Ph}**	**1·XB^{PFP}**	**1·XB^{Ph}**	**1·XB^{PFP}**	**1·XB^{Ph}**
Cl^-	18,600[e]	590	>10^5	15,800	386	394	2,920	416	548	69
Br^-	4,620	304	>10^5	7,800	116	191	4,600	1,060	2,020	263
I^-	1,230	234	24,600	2,710	37	49	5,230	1,450	2,650	537

[a] Association constants determined by Bindfit analysis of internal pyridine proton signal, errors ± < 7% unless otherwise specified. [b] Anions added as their tetrabutylammonium salts. [c] Conducted in acetone-d_6 [c] Conducted in acetone-d_6 [d] Conducted in 2.5 % D_2O-acetone-d_6 (v/v) [e] Error < 13%.[f] Errors indicated in parenthesis.

Table S5. Halide anion association constants and calculated binding enhancement factors for phenyl and pefluorophenyl appended **1·ChB** anion receptor systems in acetone-d_6.

	Anion association constants K_a / M^{-1} [a]		
Anion[b]	**1·ChB^{PFP}**	**1·ChB^{Ph}**	Binding enhancement factors α = (K_a^{PFP}/ K_a^{Ph})
Cl^-	18599 ± 2349	590 ± 5.59	31.55 ± 4.00
Br^-	4616 ± 139	304 ± 4.28	15.19 ± 0.50
I^-	1226 ± 32.4	234 ± 16.8	5.24 ± 0.40

[a] Association constants determined by Bindfit analysis of internal pyridine proton signal, conducted in acetone-d_6. [b] Anions added as their tetrabutylammonium salts.

Table S6. Halide anion association constants and calculated binding enhancement factors for phenyl and pefluorophenyl appended **1·XB** anion receptor systems in 2.5 % D_2O-acetone-d_6 (v/v).

	Anion association constants K_a / M^{-1} [a]		
Anion[b]	**1·XB^{PFP}**	**1·XB^{Ph}**	Binding enhancement factors α = (K_a^{PFP}/ K_a^{Ph})
Cl^-	2916 ± 75.4	416 ± 5.59	7.00 ± 0.20
Br^-	4603 ± 273	1056 ± 4.28	4.36 ± 0.50
I^-	5227 ± 456	1453 ± 23	3.60 ± 0.32

[a] Association constants determined by Bindfit analysis of internal pyridine proton signal, conducted in 2.5 % D_2O-acetone-d_6 (v/v). [b] Anions added as their tetrabutylammonium salts.

Table S7. Halide anion association constants and calculated binding enhancement factors for phenyl and pefluorophenyl appended **1·XB** anion receptor systems in 5 % D_2O-acetone-d_6 (v/v).

	Anion association constants K_a / M^{-1} [a]		
Anion[b]	**1·XB^{PFP}**	**1·XB^{Ph}**	Binding enhancement factors α = (K_a^{PFP}/ K_a^{Ph})
Cl^-	573 ± 14.6	77.1 ± 2.0	7.44 ± 0.27
Br^-	2023 ± 45.4	264 ± 7.9	7.68 ± 0.29
I^-	2689 ± 114	563 ± 10.5	4.78 ± 0.22

[a] Association constants determined by Bindfit analysis of internal pyridine proton signal, conducted in 2.5 % D_2O-acetone-d_6 (v/v). [b] Anions added as their tetrabutylammonium salts.

Table S8. Halide anion association constants and calculated binding enhancement factors for phenyl and pefluorophenyl appended **1·HB** anion receptor systems in acetone-d_6.

	Anion association constants K_a / M^{-1} [a]		
Anion[b]	**1·HB^{PFP}**	**1·HB^{Ph}**	Binding enhancement factors α = (K_a^{PFP}/ K_a^{Ph})
Cl^-	390 ± 14.6	454 ± 10.3	0.86 ± 0.057
Br^-	128 ± 4.06	168 ± 4.88	0.76 ± 0.033
I^-	48.4 ± 2.67	56.5 ± 1.71	0.86 ± 0.054

[a] Association constants determined by Bindfit analysis of internal pyridine proton signal, conducted in 2.5 % D_2O-acetone-d_6 (v/v). [b] Anions added as their tetrabutylammonium salts

Table S9. Halide anion association constants and calculated ρ values from Hammett plot analysis of the **2·ChB** receptor series.

	Anion association constants K_a / M^{-1} [a] for **2·ChB^R** series				
Anion[b]	**mF**	**2F**	**3F**	ρ[c]	r[d]
Cl^-	987[e]	2070	2950[e]	0.91 ± 0.11	0.99
Br^-	592	966	145	0.81 ± 0.11	0.98
I^-	217	547	497	0.53 ± 0.21	0.87

[a] Association constants determined by Bindfit analysis of internal pyridine proton signal, errors ± < 5% unless otherwise specified, conducted in acetone-d_6 [b] Anions added as their tetrabutylammonium salts. [c] ρ refers to the value from the Hammett equation, errors indicated in parenthesis. [d] Pearson's correlation coefficient.

Table S10. Halide anion association constants for the **3·ChB^R** receptor systems.

	Anion association constants K_a / M^{-1} for **3·ChB^R** series [a]												
Anion[b]	**pOMe**	**pMe**	**Ph[c]**	**pF**	**pCl**	**mF**	**mCF3**	**pCF3**	**2F[c]**	**3F[c]**	**2CF3**	ρ[g]	r[h]
Cl^-	450	1140	1,360	1,610	4,790	14,000[f]	$>10^5$[d]	$>10^5$[d]	$>10^5$[d]	$>10^5$[d]	$>10^5$[d]	2.20 ± 0.29	0.97
Br^-	230	518	822	863	2,540	2,740	7,540	7,270	5,180	11,800[f]	19,900[f]	1.59 ± 0.13	0.97
I^-	NB[e]	NB[e]	119	398	674	592	1450	1,620	1,040	1,750	3,980	1.28 ± 0.23	0.90

[a] Association constants determined by Bindfit analysis of Te-aryl chemical shift perturbations unless otherwise specified, errors ≤ 10% unless otherwise specified, conducted in acetone-d_6. [b] Anions added as their tetrabutylammonium salts. [c] Determined by monitoring of internal pyridine proton signal. [d] Too large to be accurately quantified by ^1H NMR titration. [e] No binding. [f] Error ≤ 26%. [g] ρ refers to the value from the Hammett equation [h] Pearson's correlation coefficient

References

[1] L.-M. Jin, X. Xu, H. Lu, X. Cui, L. Wojtas, X. P. Zhang, *Angew. Chem. Int. Ed.* **2013**, *52*, 5309–5313.
[2] T. Bunchuay, A. Docker, U. Eiamprasert, P. Surawatanawong, A. Brown, P. D. Beer, *Angew. Chem. Int. Ed.* **2020**, *59*, 12007–12012.
[3] D. Bornemann, C. R. Pitts, C. J. Ziegler, E. Pietrasiak, N. Trapp, S. Kueng, N. Santschi, A. Togni, *Angew. Chem. Int. Ed.* **2019**, *58*, 12604–12608.
[4] J. Liu, M. Qiu, X. Zhou, *Synth. Commun.* **1990**, *20*, 2759–2767.
[5] J. Cosier, A. M. Glazer, *J. Appl. Crystallogr.* **1986**, *19*, 105–107.
